# Supplementary material for: Mitochondrial fatty acid oxidation regulates adult muscle stem cell function through modulating metabolic flux and protein acetylation
Source: EMBO J. 2025 Mar 10;44(9):2566–95. doi: 10.1038/s44318-025-00397-1 (PMC12048568; doi:10.1038/s44318-025-00397-1)
Supplement: Supplementary file 1 — Appendix Table S1 [file 44318_2025_397_MOESM1_ESM.pdf]

Table of Contents

Appendix Table S1 2

Appendix Table S1

List of intermediates detected in this study

Multiple reaction monitoring (MRM) Table				
Compound Group	Compound Name	Precursor Ion	Product Ion	Collision Energy
TCA Cycle	a-ketoglutaric acid [M+0]	462.1	91.1	25
	a-ketoglutaric acid [M+1]	463.1	91.1	25
	a-ketoglutaric acid [M+2]	464.1	91.1	25
	a-ketoglutaric acid [M+3]	465.1	91.1	25
	a-ketoglutaric acid [M+4]	466.1	91.1	25
	a-ketoglutaric acid [M+5]	467.1	91.1	25
TCA Cycle	citric acid [M+0]	508.1	91.1	25
	citric acid [M+1]	509.1	91.1	25
	citric acid [M+2]	510.1	91.1	25
	citric acid [M+3]	511.1	91.1	25
	citric acid [M+4]	512.1	91.1	25
	citric acid [M+5]	513.1	91.1	25
	citric acid [M+6]	514.1	91.1	25
TCA Cycle	isocitric acid [M+0]	508.1	91.1	25
	isocitric acid [M+1]	509.1	91.1	25
	isocitric acid [M+2]	510.1	91.1	25
	isocitric acid [M+3]	511.1	91.1	25
	isocitric acid [M+4]	512.1	91.1	25
	isocitric acid [M+5]	513.1	91.1	25
	isocitric acid [M+6]	514.1	91.1	25
TCA Cycle	lactic acid [M+0]	196	91.1	25
	lactic acid [M+1]	197	91.1	25
	lactic acid [M+2]	198	91.1	25
	lactic acid [M+3]	199	91.1	25
TCA Cycle	malic acid [M+0]	345.1	91.1	25
	malic acid [M+1]	346.1	91.1	25
	malic acid [M+2]	347.1	91.1	25
	malic acid [M+3]	348.1	91.1	25
	malic acid [M+4]	349.1	91.1	25
TCA Cycle	oxaloacetic acid [M+0]	448.1	91.1	25
	oxaloacetic acid [M+1]	449.1	91.1	25
	oxaloacetic acid [M+2]	450.1	91.1	25
	oxaloacetic acid [M+3]	451.1	91.1	25
	oxaloacetic acid [M+4]	452.1	91.1	25
TCA Cycle	pyruvate [M+0]	299.1	91.1	25
	pyruvate [M+1]	300.1	91.1	25
	pyruvate [M+2]	301.1	91.1	25
	pyruvate [M+3]	302.1	91.1	25
TCA Cycle	succinic acid [M+0]	329.1	91.1	25
	succinic acid [M+1]	330.1	91.1	25
	succinic acid [M+2]	331.1	91.1	25
	succinic acid [M+3]	332.1	91.1	25
	succinic acid [M+4]	333.1	91.1	25
