# Supplementary figures and images for: Mitochondrial fatty acid oxidation regulates adult muscle stem cell function through modulating metabolic flux and protein acetylation
Source: EMBO J. 2025 Mar 10;44(9):2566–95. doi: 10.1038/s44318-025-00397-1 (PMC12048568; doi:10.1038/s44318-025-00397-1)

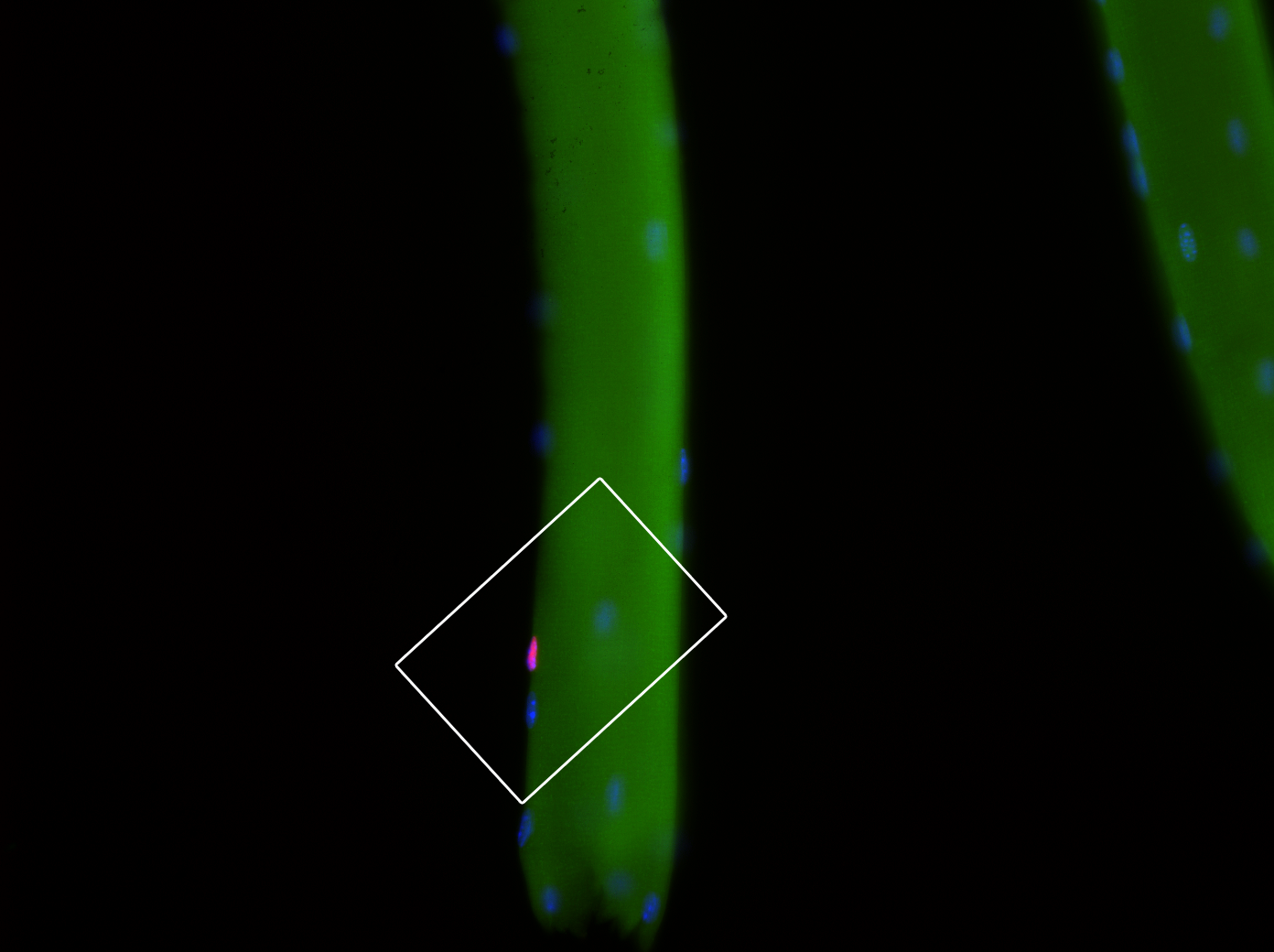

Supplement: Supplementary file 3 — Source data Fig. 1 [file 44318_2025_397_MOESM3_ESM.zip › Figure 1/Figure 1F/0h-Pax7-Cpt2-Overlay-200x.tif]

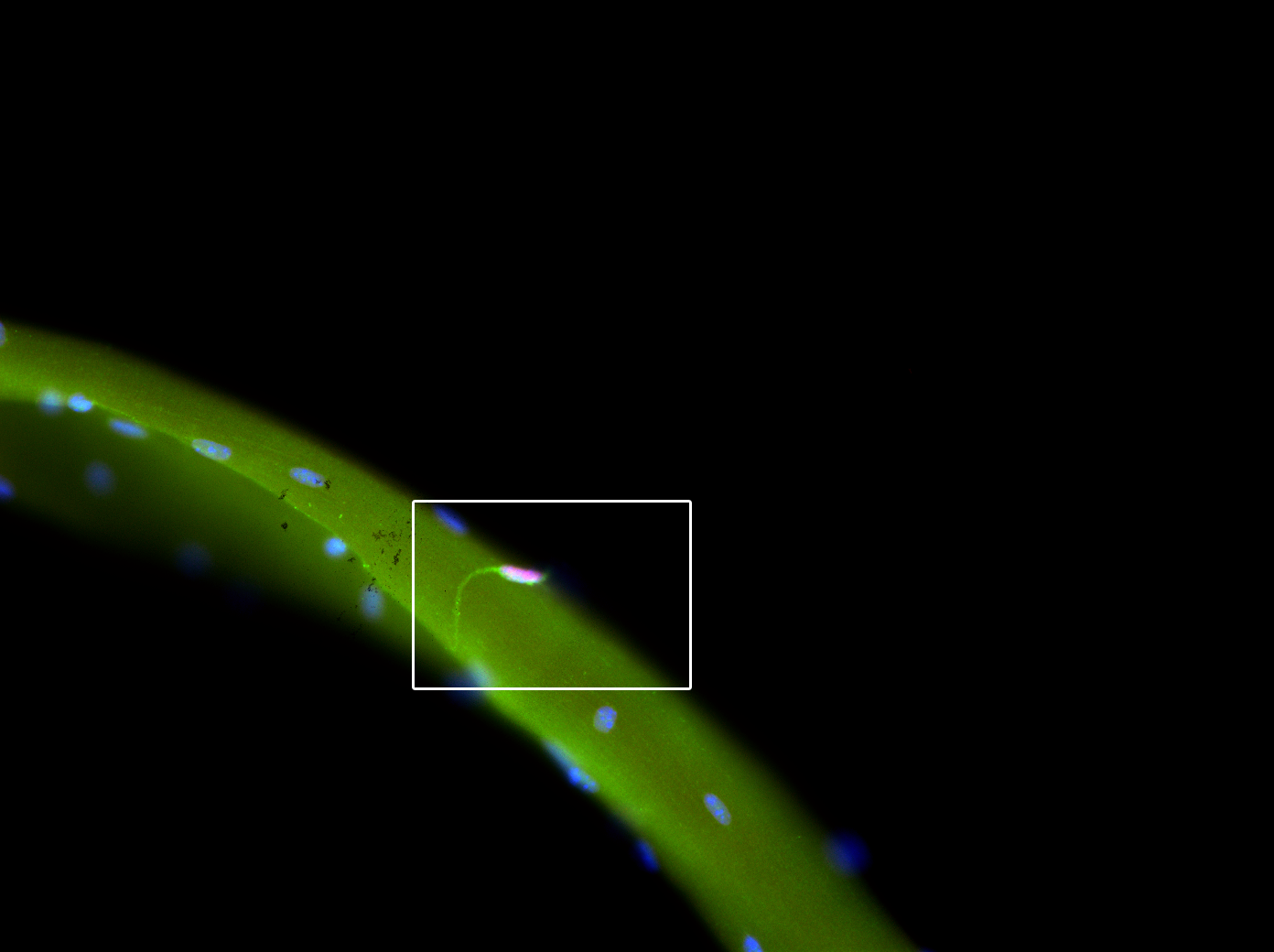

Supplement: Supplementary file 3 — Source data Fig. 1 [file 44318_2025_397_MOESM3_ESM.zip › Figure 1/Figure 1F/24h-Pax7-Cpt2-Overlay-200x.tif]

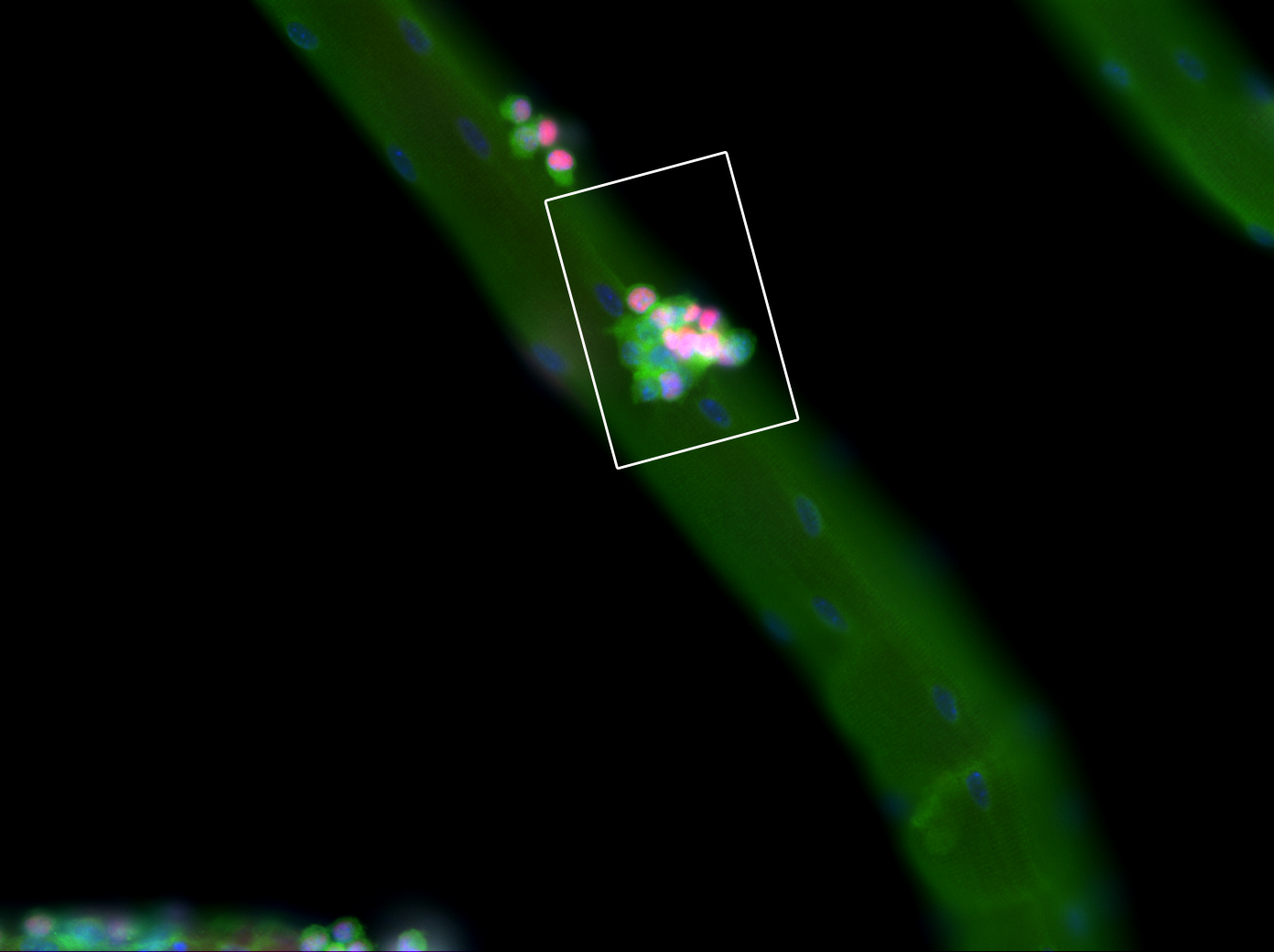

Supplement: Supplementary file 3 — Source data Fig. 1 [file 44318_2025_397_MOESM3_ESM.zip › Figure 1/Figure 1F/72h-Pax7-Cpt2-Overlay-200x.tif]

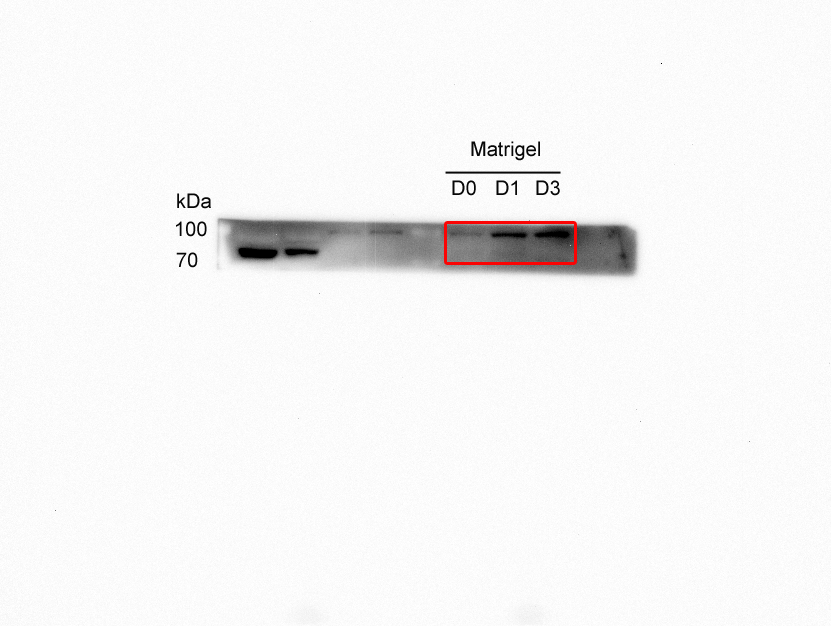

Supplement: Supplementary file 3 — Source data Fig. 1 [file 44318_2025_397_MOESM3_ESM.zip › Figure 1/Figure 1H/Immunoblot-Cpt2/CPT2_MB_Diff_013.tif]

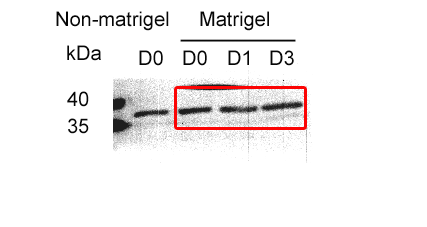

Supplement: Supplementary file 3 — Source data Fig. 1 [file 44318_2025_397_MOESM3_ESM.zip › Figure 1/Figure 1H/Immunoblot-Cpt2/GAPDH_MB_Diff_013.tif]

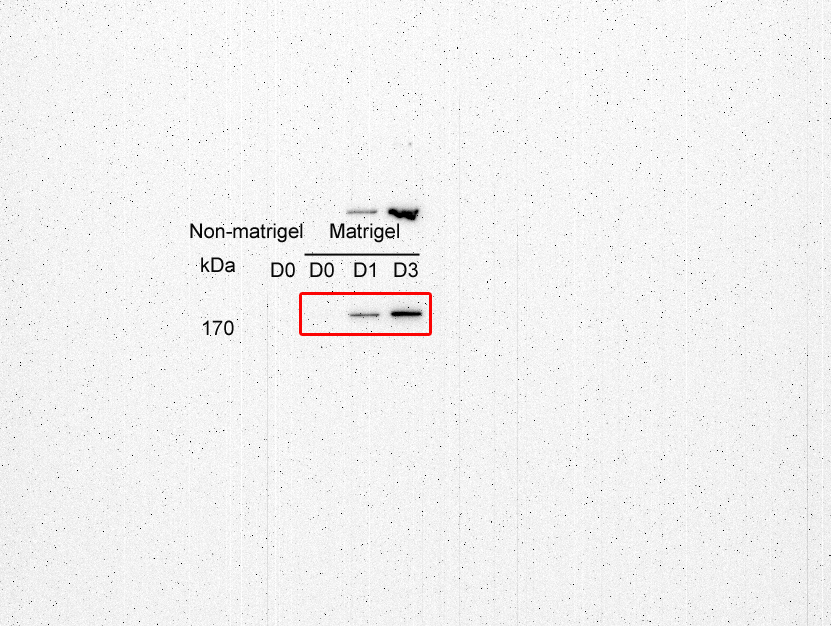

Supplement: Supplementary file 3 — Source data Fig. 1 [file 44318_2025_397_MOESM3_ESM.zip › Figure 1/Figure 1H/Immunoblot-Cpt2/MF20_MB_Diff_013.tif]

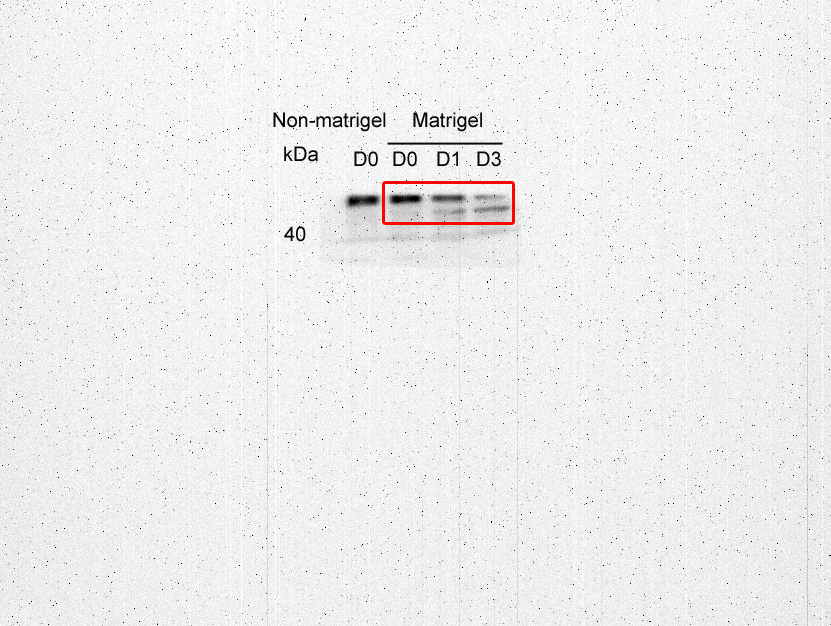

Supplement: Supplementary file 3 — Source data Fig. 1 [file 44318_2025_397_MOESM3_ESM.zip › Figure 1/Figure 1H/Immunoblot-Cpt2/MyoD_MB_Diff_013.tif.tif]

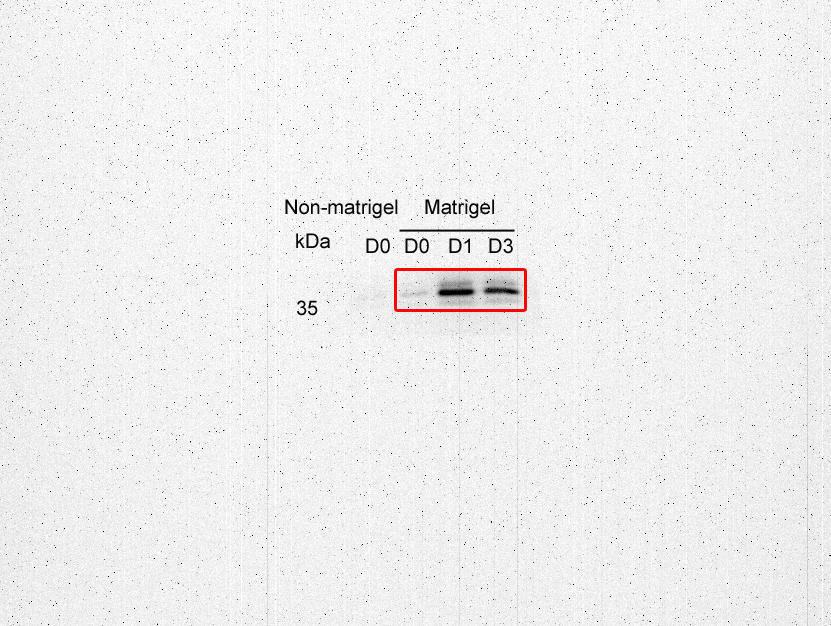

Supplement: Supplementary file 3 — Source data Fig. 1 [file 44318_2025_397_MOESM3_ESM.zip › Figure 1/Figure 1H/Immunoblot-Cpt2/MyoG_MB_Diff_013.tif]

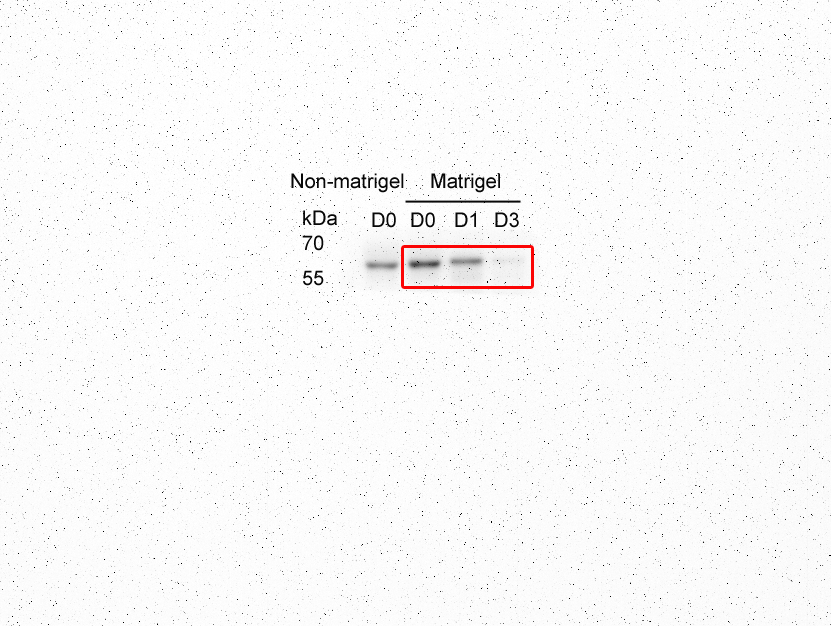

Supplement: Supplementary file 3 — Source data Fig. 1 [file 44318_2025_397_MOESM3_ESM.zip › Figure 1/Figure 1H/Immunoblot-Cpt2/Pax7_MB_Diff_013.tif]

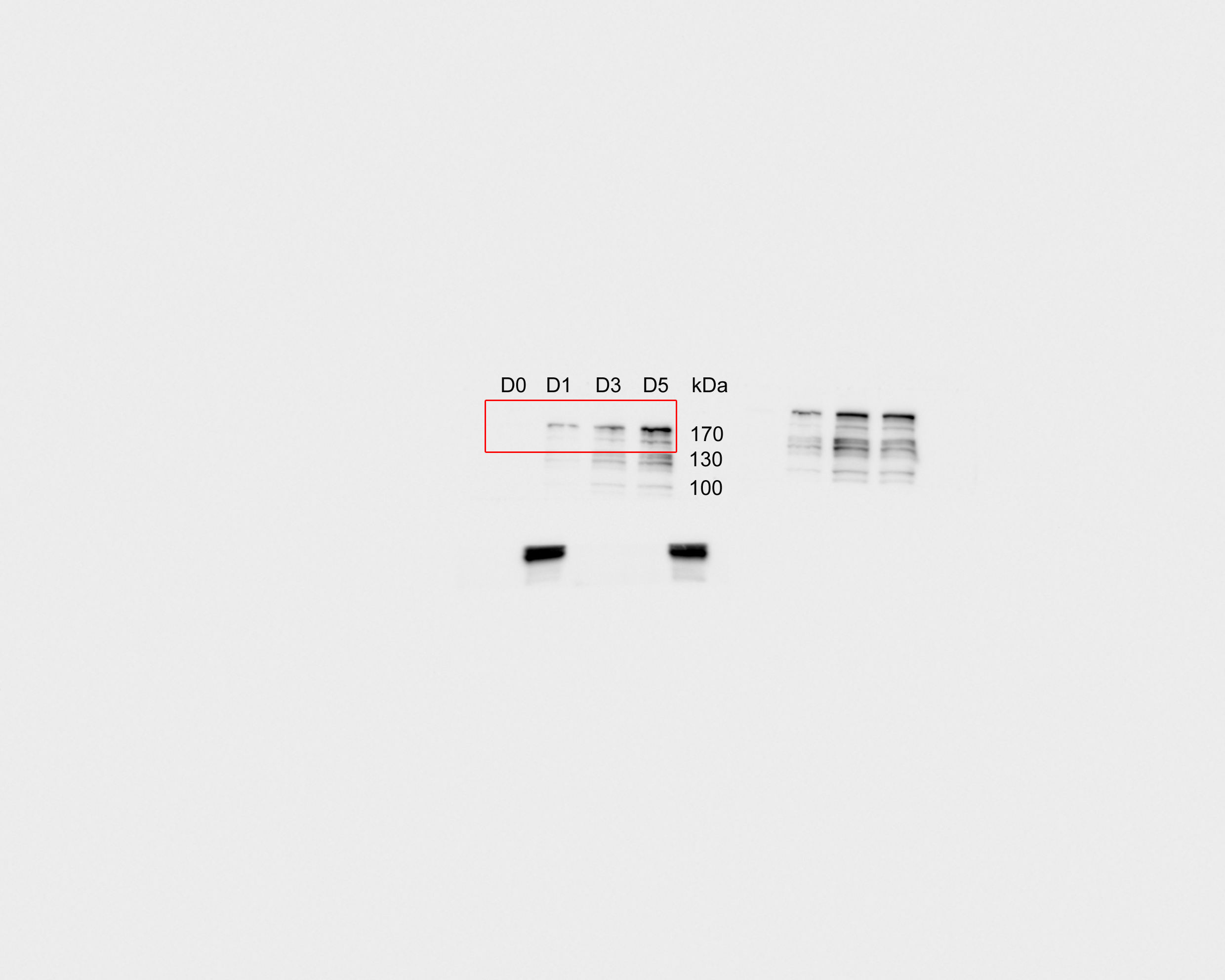

Supplement: Supplementary file 3 — Source data Fig. 1 [file 44318_2025_397_MOESM3_ESM.zip › Figure 1/Figure 1H/Immunoblot-OxPhos/MF20_MB_Diff_0135.tif]

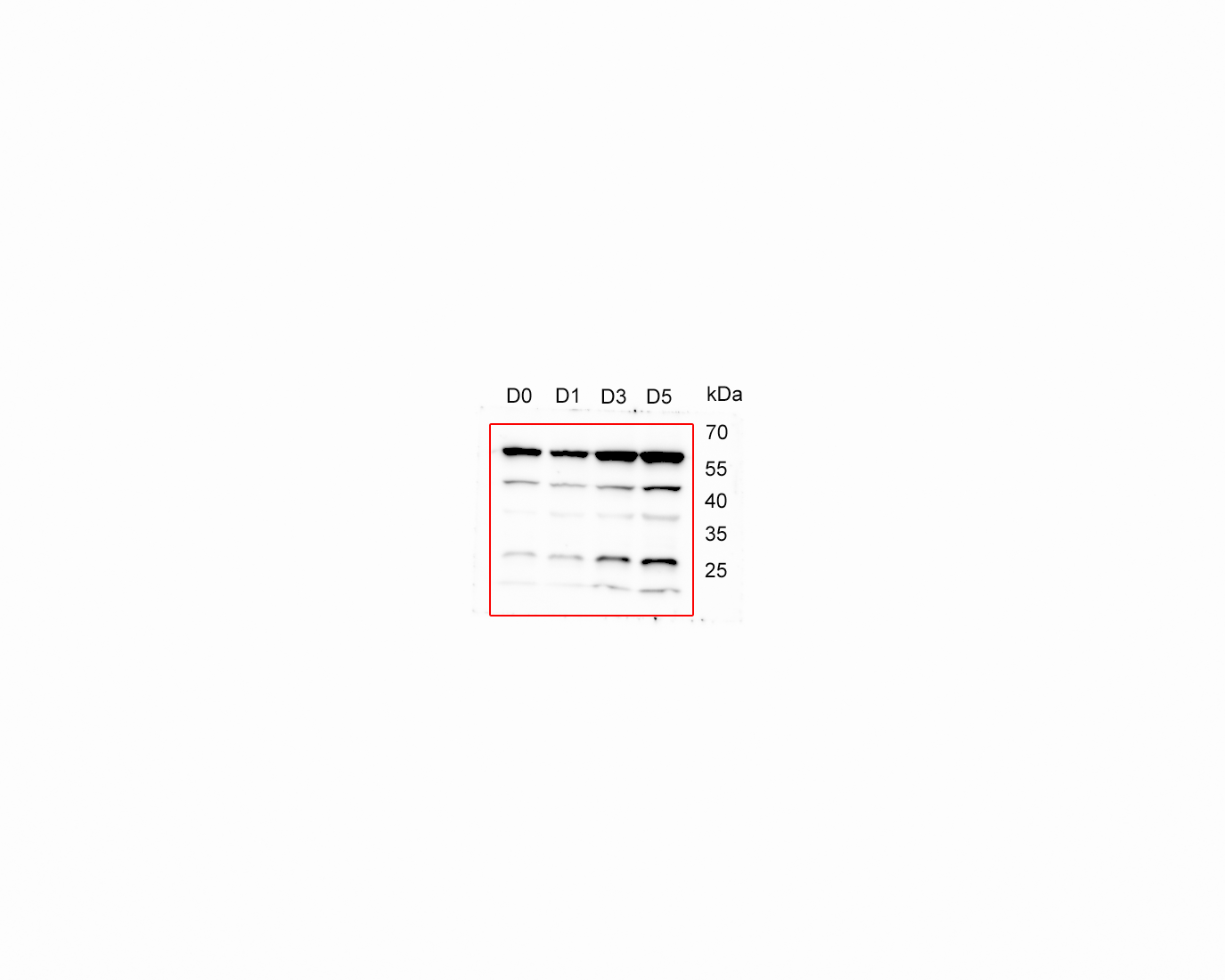

Supplement: Supplementary file 3 — Source data Fig. 1 [file 44318_2025_397_MOESM3_ESM.zip › Figure 1/Figure 1H/Immunoblot-OxPhos/OxPho_MB_Diff_0135.tif]

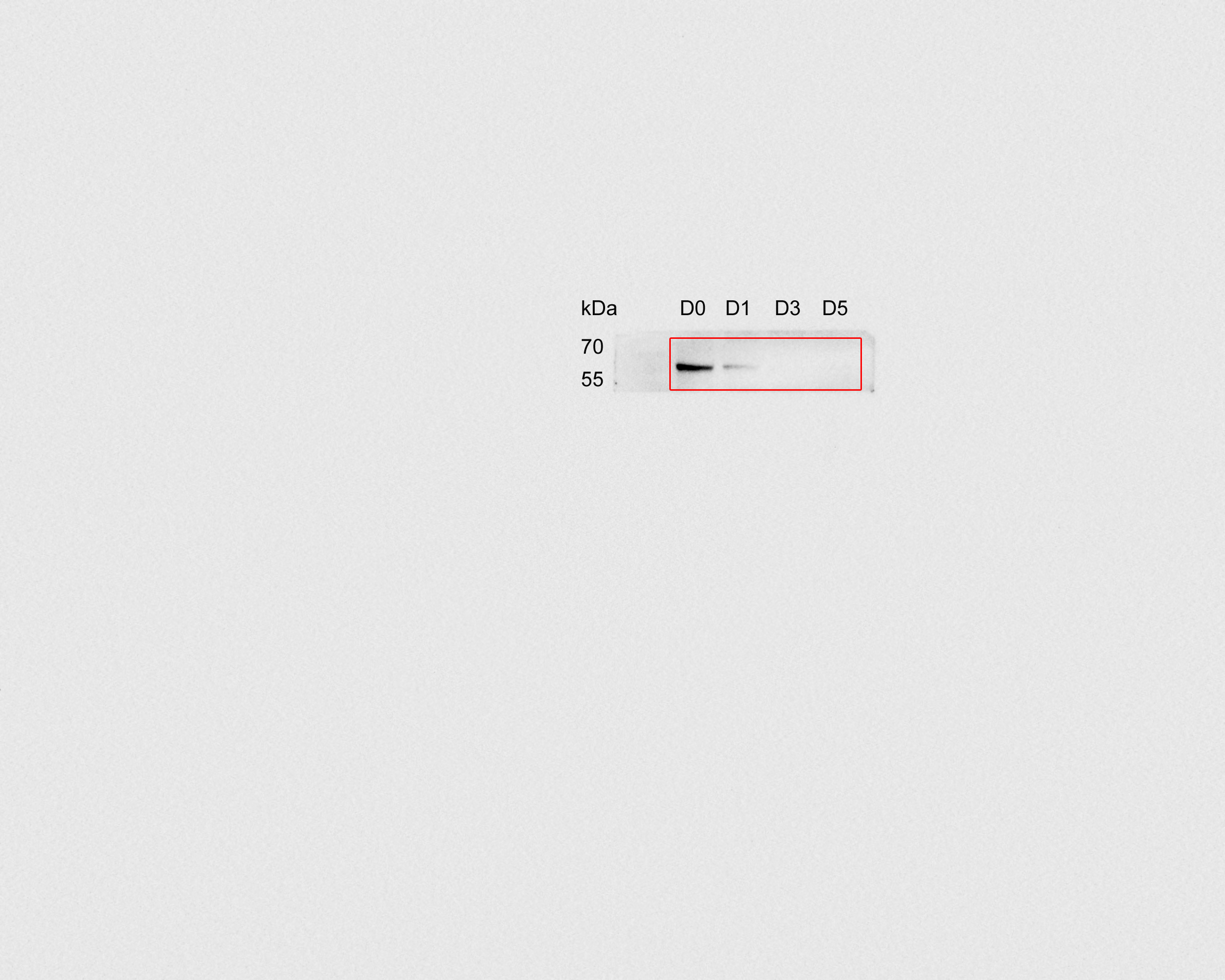

Supplement: Supplementary file 3 — Source data Fig. 1 [file 44318_2025_397_MOESM3_ESM.zip › Figure 1/Figure 1H/Immunoblot-OxPhos/Pax7_MB_Diff_0135.tif]

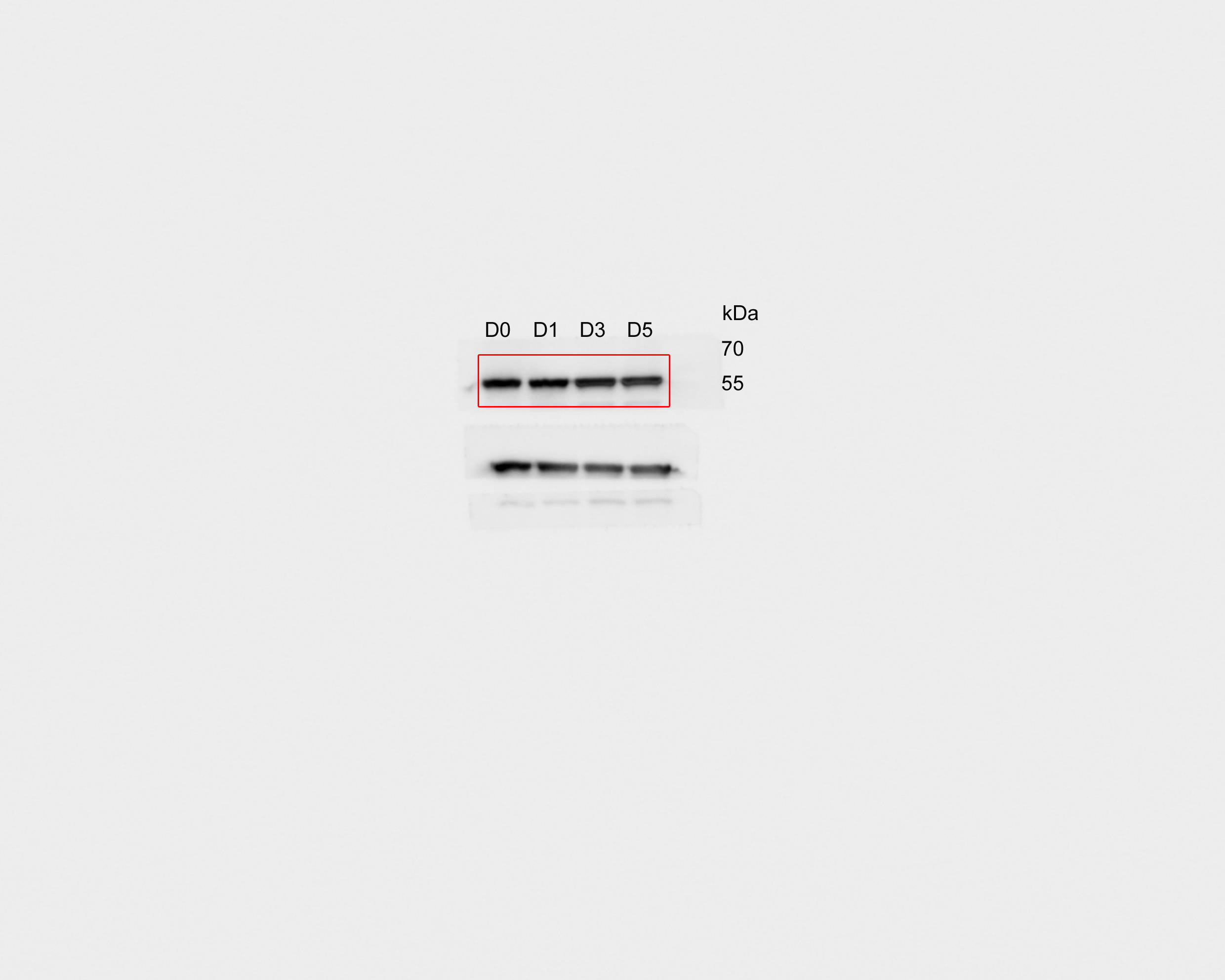

Supplement: Supplementary file 3 — Source data Fig. 1 [file 44318_2025_397_MOESM3_ESM.zip › Figure 1/Figure 1H/Immunoblot-OxPhos/Tubulin_MB_Diff_0135.tif]

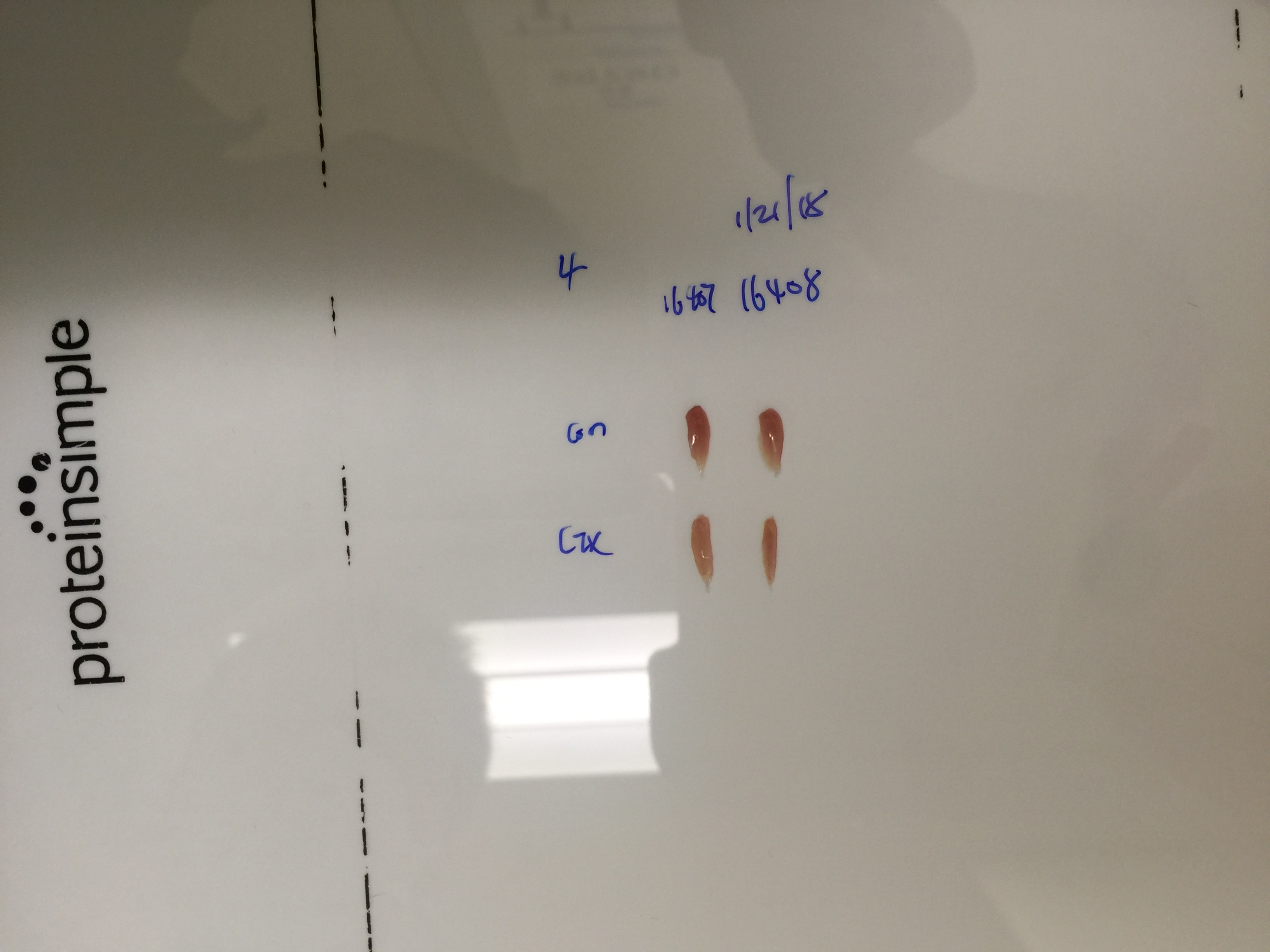

Supplement: Supplementary file 4 — Source data Fig. 2 [file 44318_2025_397_MOESM4_ESM.zip › Figure 2/Figure 2B/TA muscle from WT and Cpt2PKO mice at 5.5 dpi.JPG]

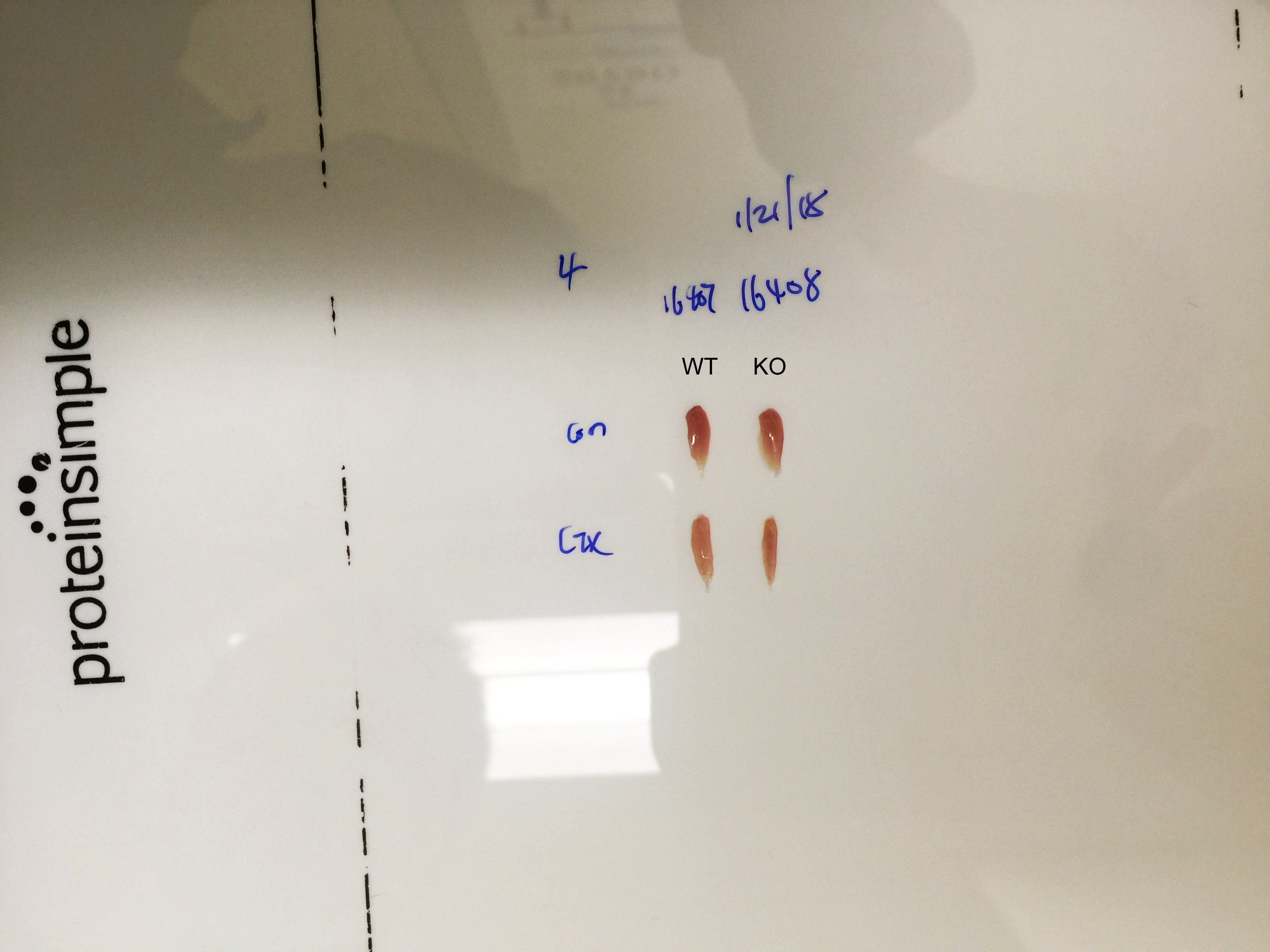

Supplement: Supplementary file 4 — Source data Fig. 2 [file 44318_2025_397_MOESM4_ESM.zip › Figure 2/Figure 2B/TA muscle from WT and Cpt2PKO mice at 5.5 dpi.tif]

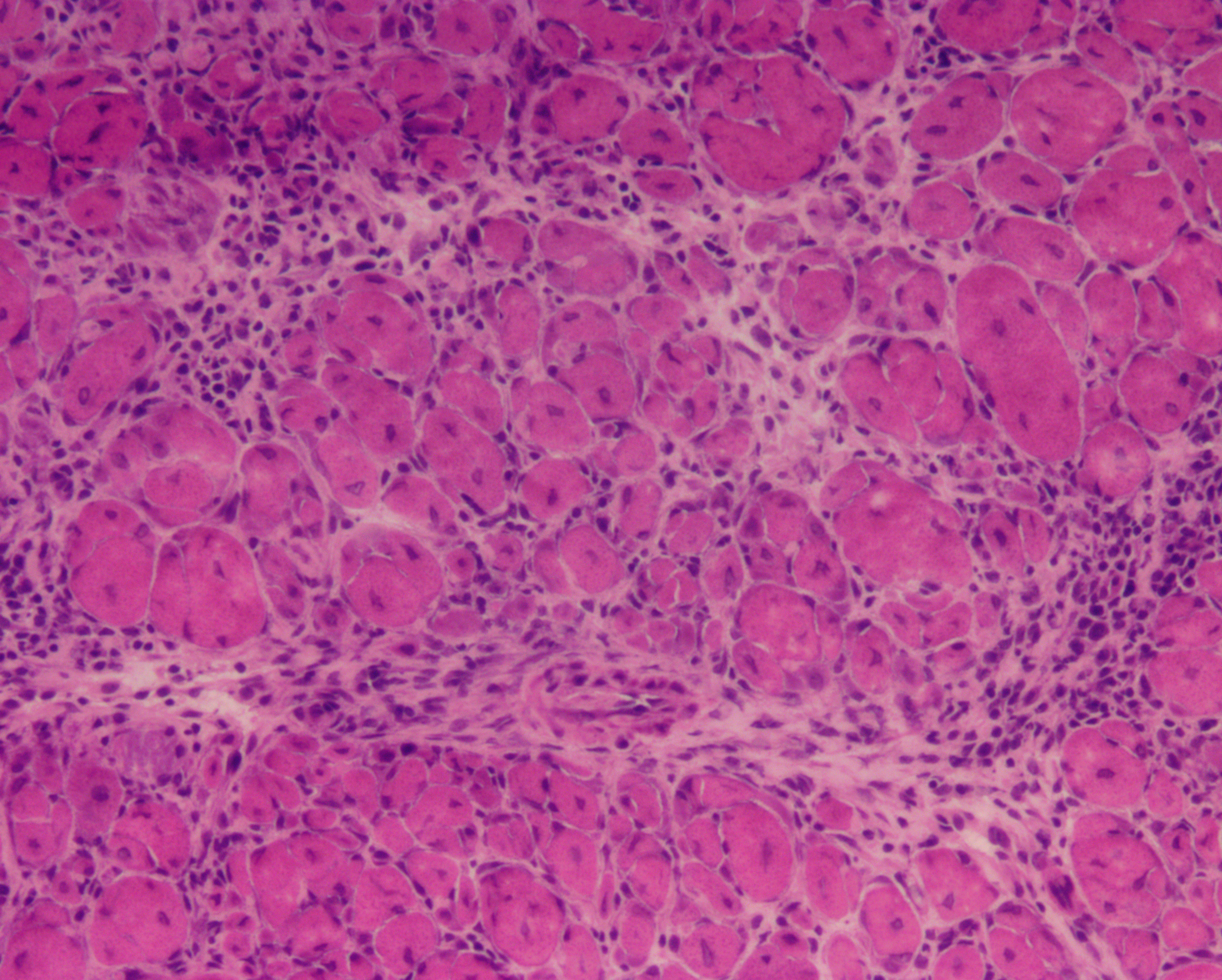

Supplement: Supplementary file 4 — Source data Fig. 2 [file 44318_2025_397_MOESM4_ESM.zip › Figure 2/Figure 2D/HE Staining_D10_KO_TA.tif]

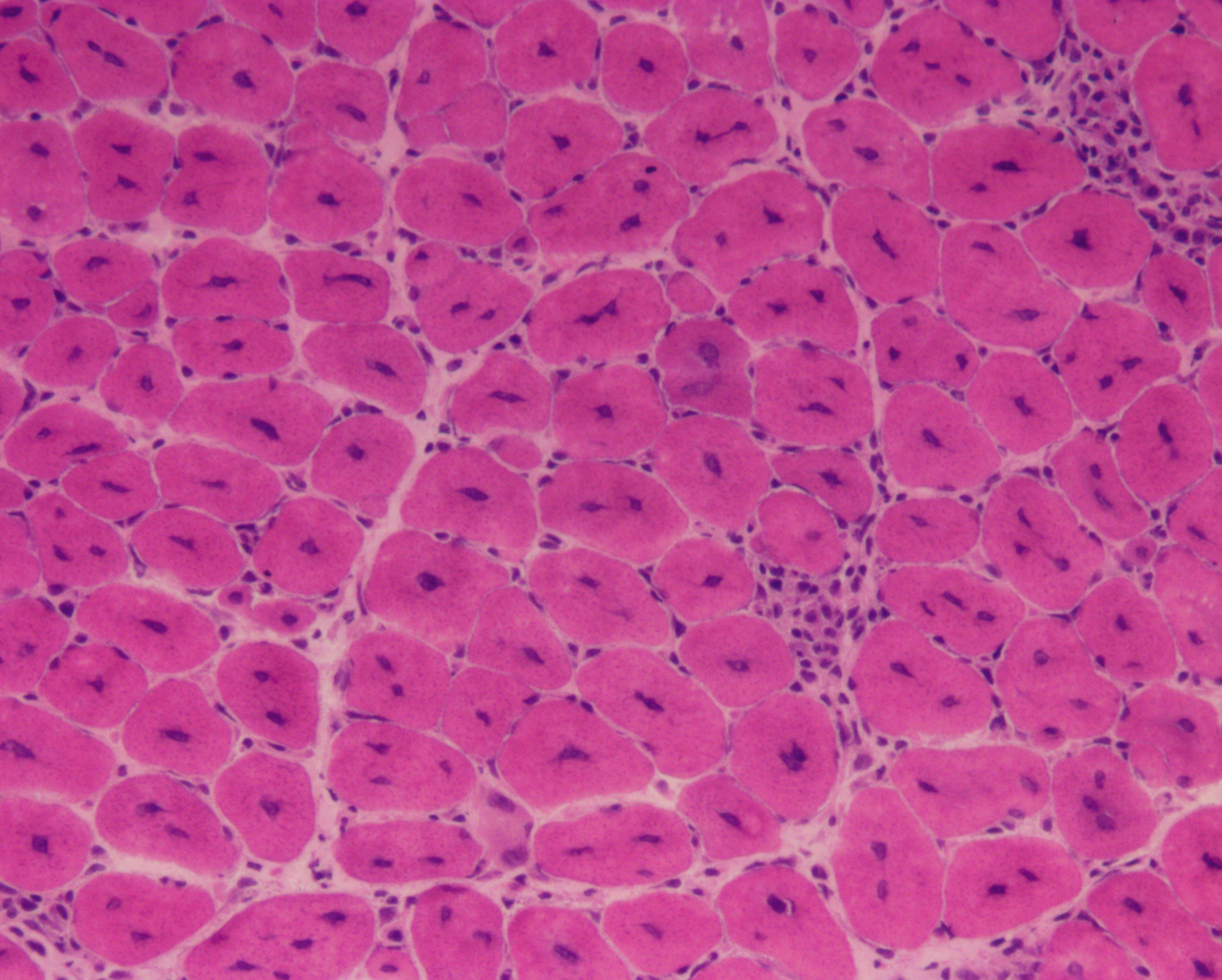

Supplement: Supplementary file 4 — Source data Fig. 2 [file 44318_2025_397_MOESM4_ESM.zip › Figure 2/Figure 2D/HE Staining_D10_WT_TA.tif]

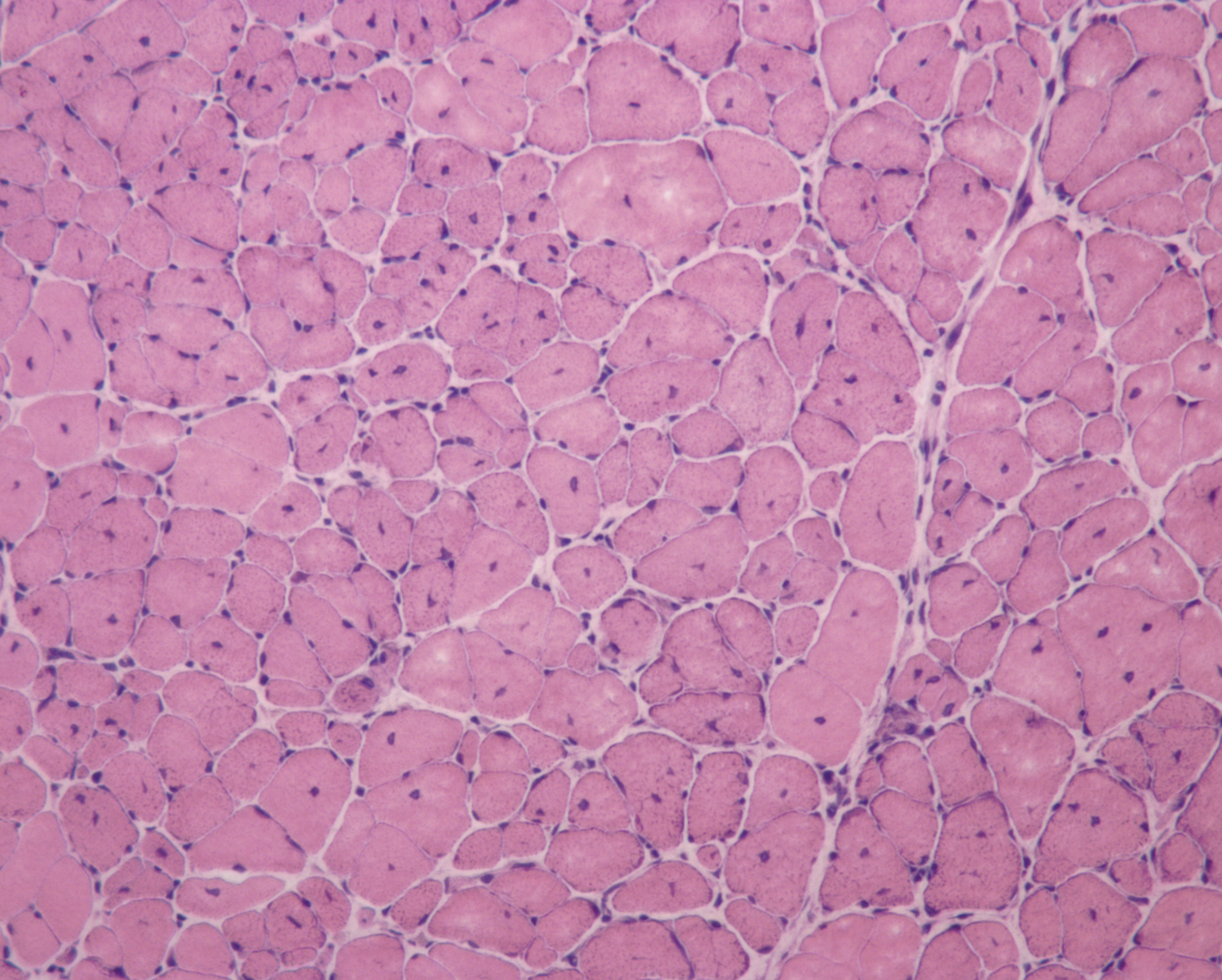

Supplement: Supplementary file 4 — Source data Fig. 2 [file 44318_2025_397_MOESM4_ESM.zip › Figure 2/Figure 2D/HE Staining_D21_KO_TA.tif]

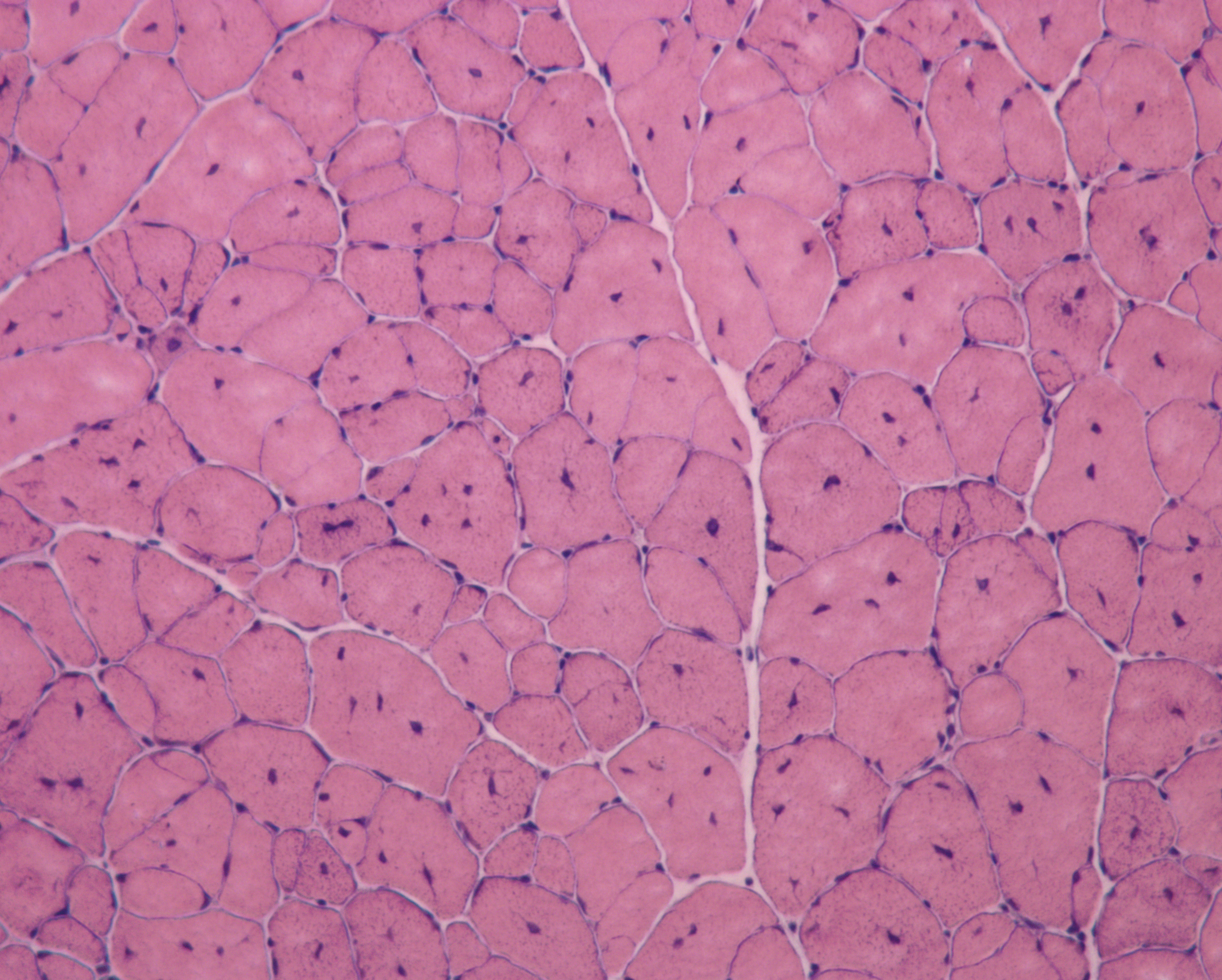

Supplement: Supplementary file 4 — Source data Fig. 2 [file 44318_2025_397_MOESM4_ESM.zip › Figure 2/Figure 2D/HE Staining_D21_WT_TA.tif]

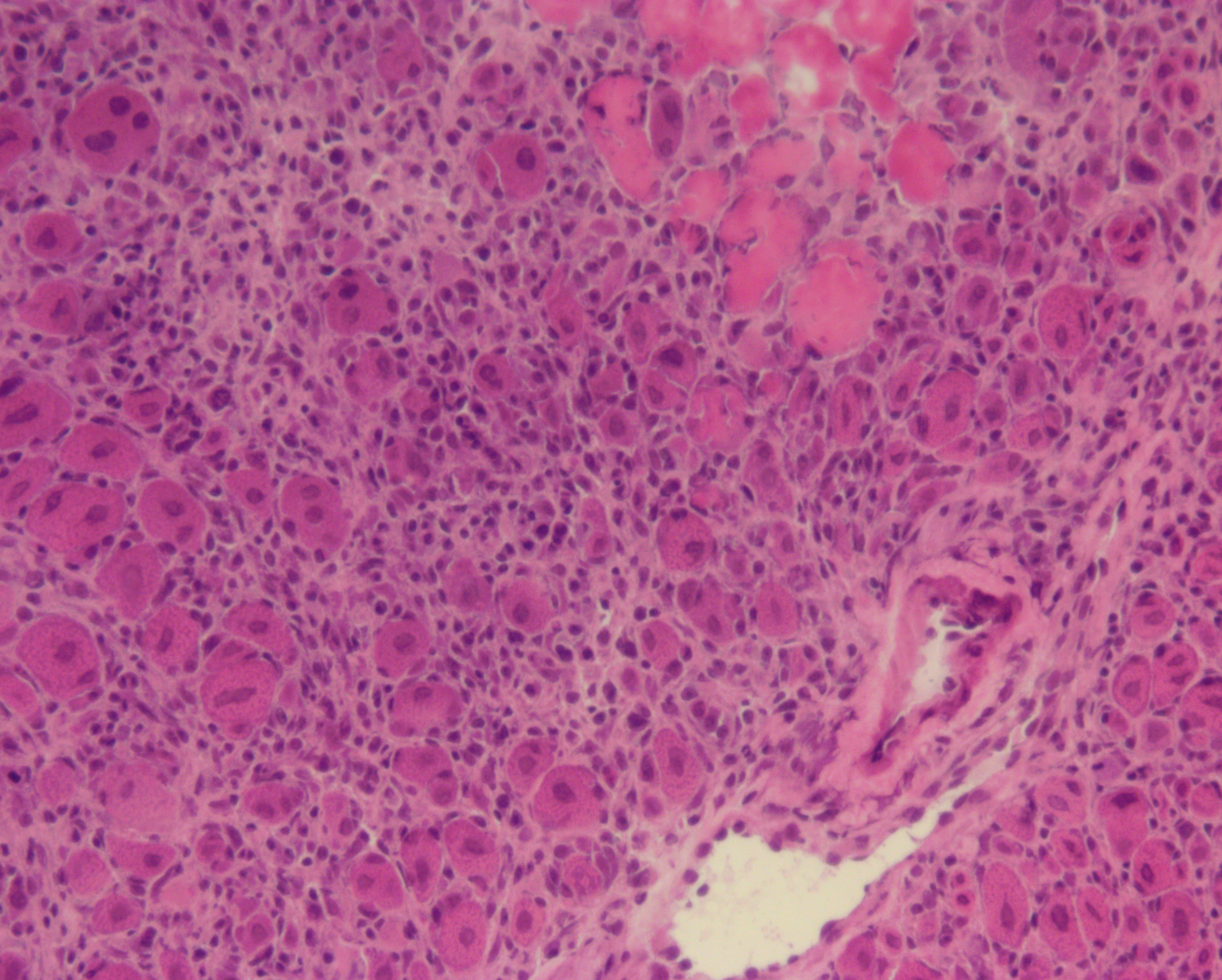

Supplement: Supplementary file 4 — Source data Fig. 2 [file 44318_2025_397_MOESM4_ESM.zip › Figure 2/Figure 2D/HE Staining_D5.5_KO_TA.tif]

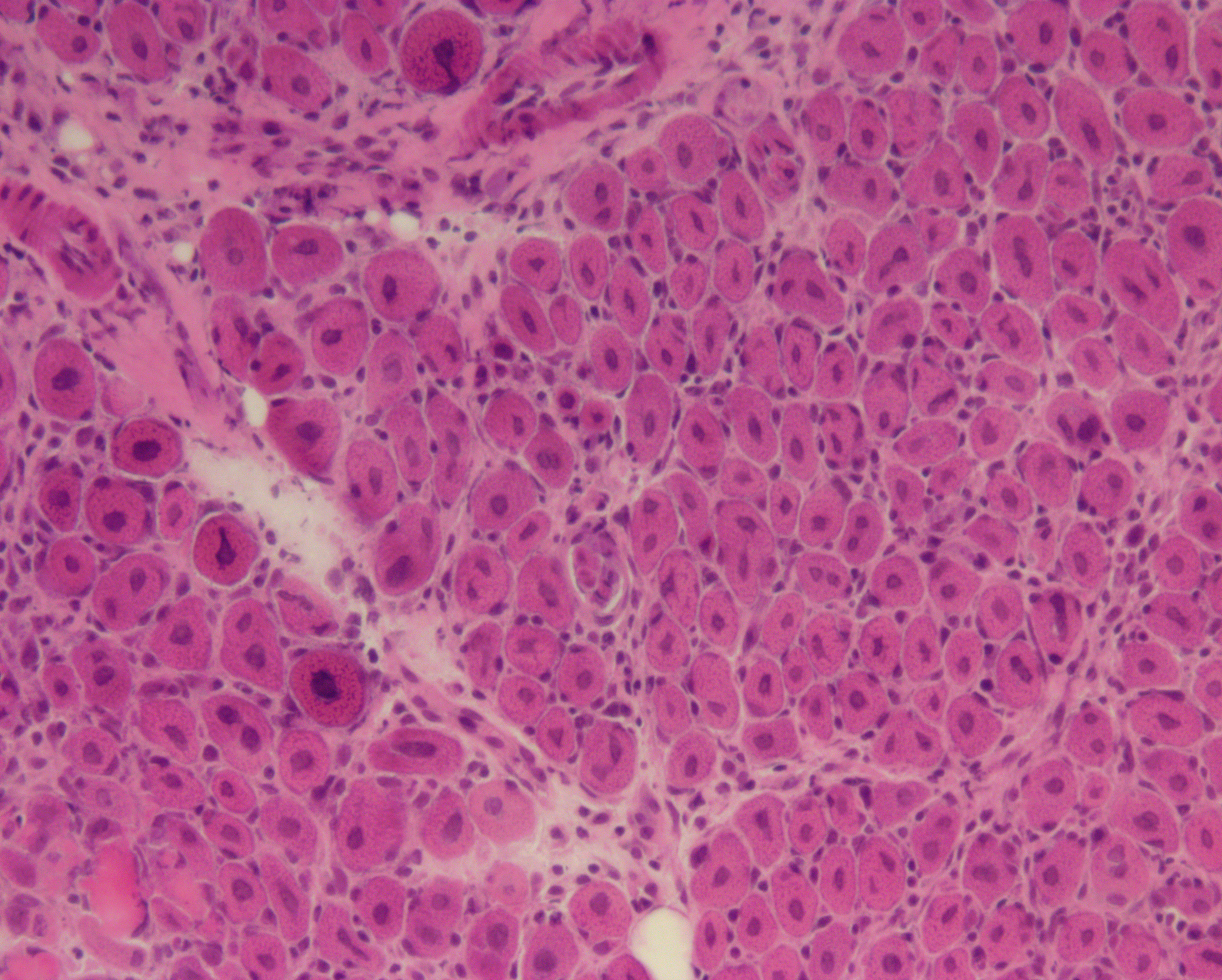

Supplement: Supplementary file 4 — Source data Fig. 2 [file 44318_2025_397_MOESM4_ESM.zip › Figure 2/Figure 2D/HE Staining_D5.5_WT_TA.tif]

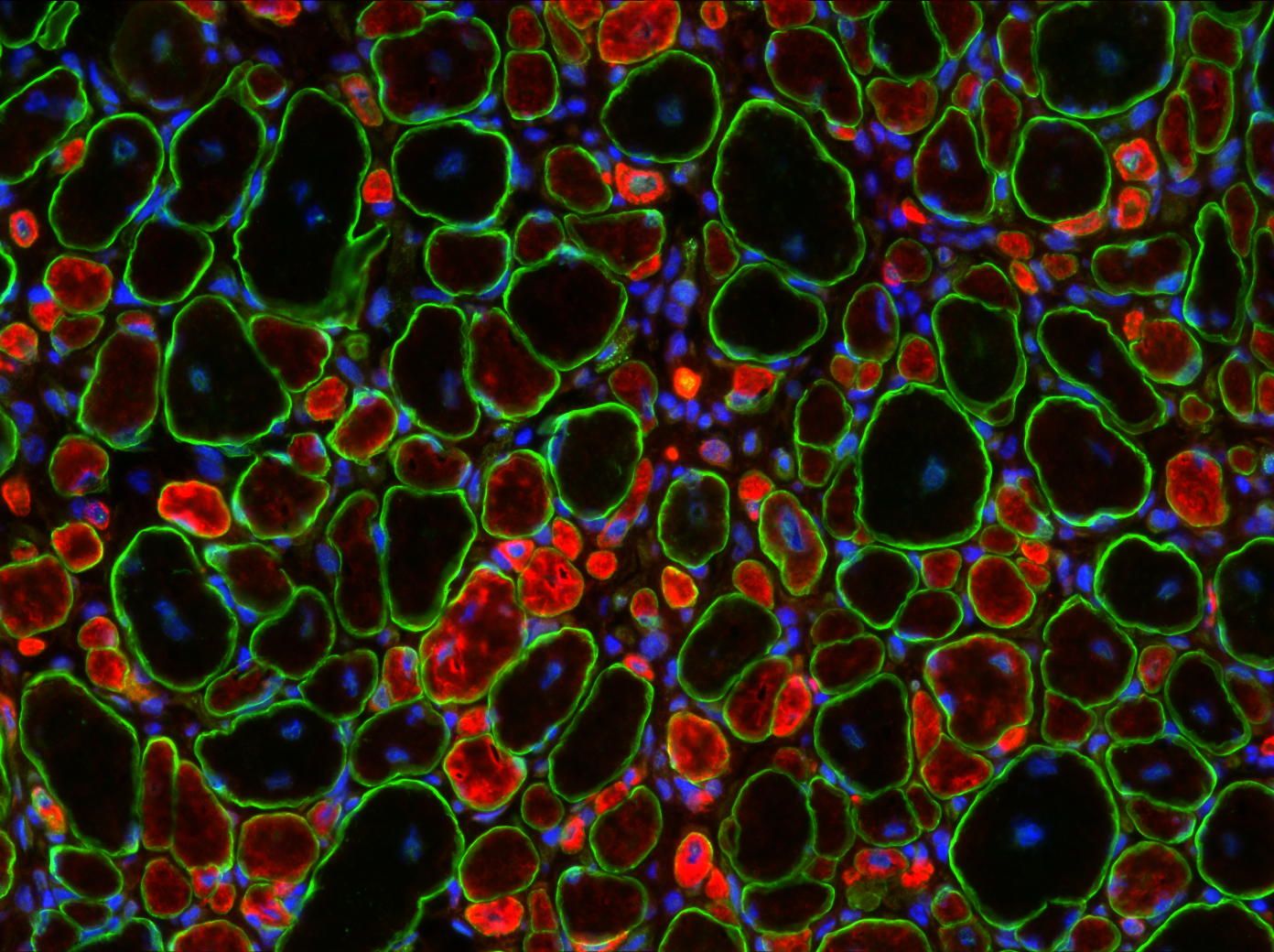

Supplement: Supplementary file 4 — Source data Fig. 2 [file 44318_2025_397_MOESM4_ESM.zip › Figure 2/Figure 2E/IF_eMyHC+Dystrophin_D10_KO_TA.tif]

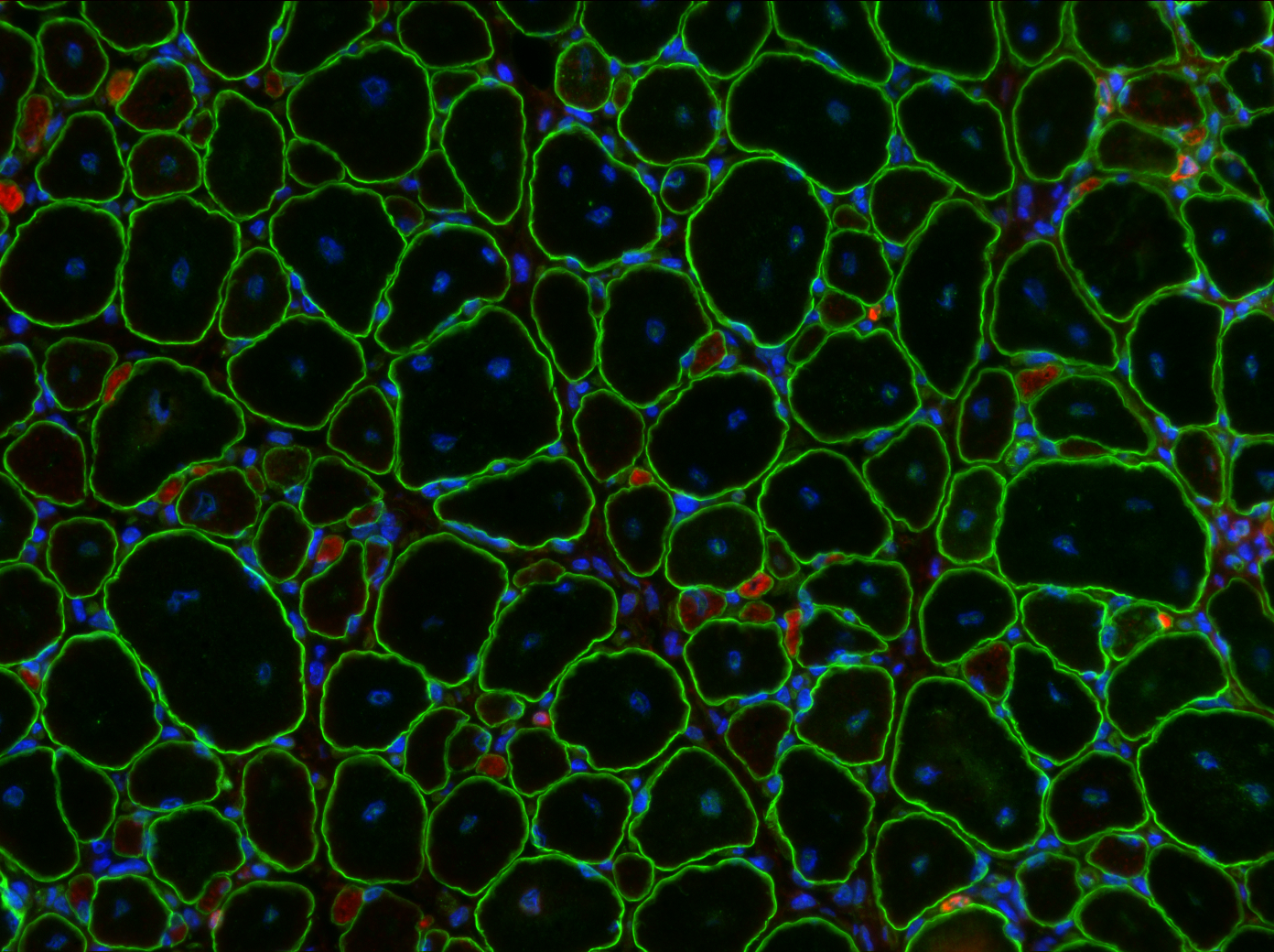

Supplement: Supplementary file 4 — Source data Fig. 2 [file 44318_2025_397_MOESM4_ESM.zip › Figure 2/Figure 2E/IF_eMyHC+Dystrophin_D10_WT_TA.tif]

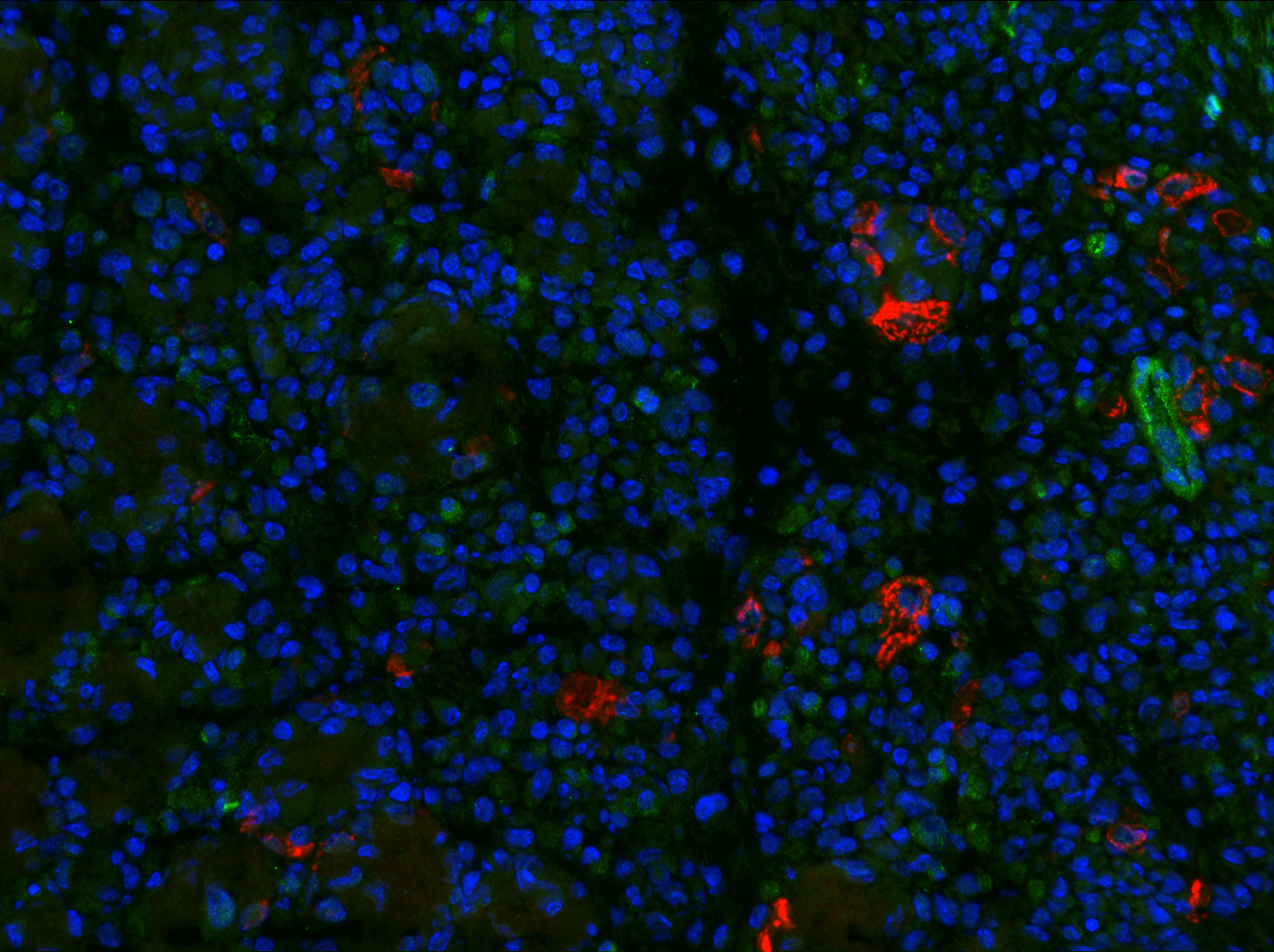

Supplement: Supplementary file 4 — Source data Fig. 2 [file 44318_2025_397_MOESM4_ESM.zip › Figure 2/Figure 2E/IF_eMyHC+Dystrophin_D3.5_KO_TA.tif]

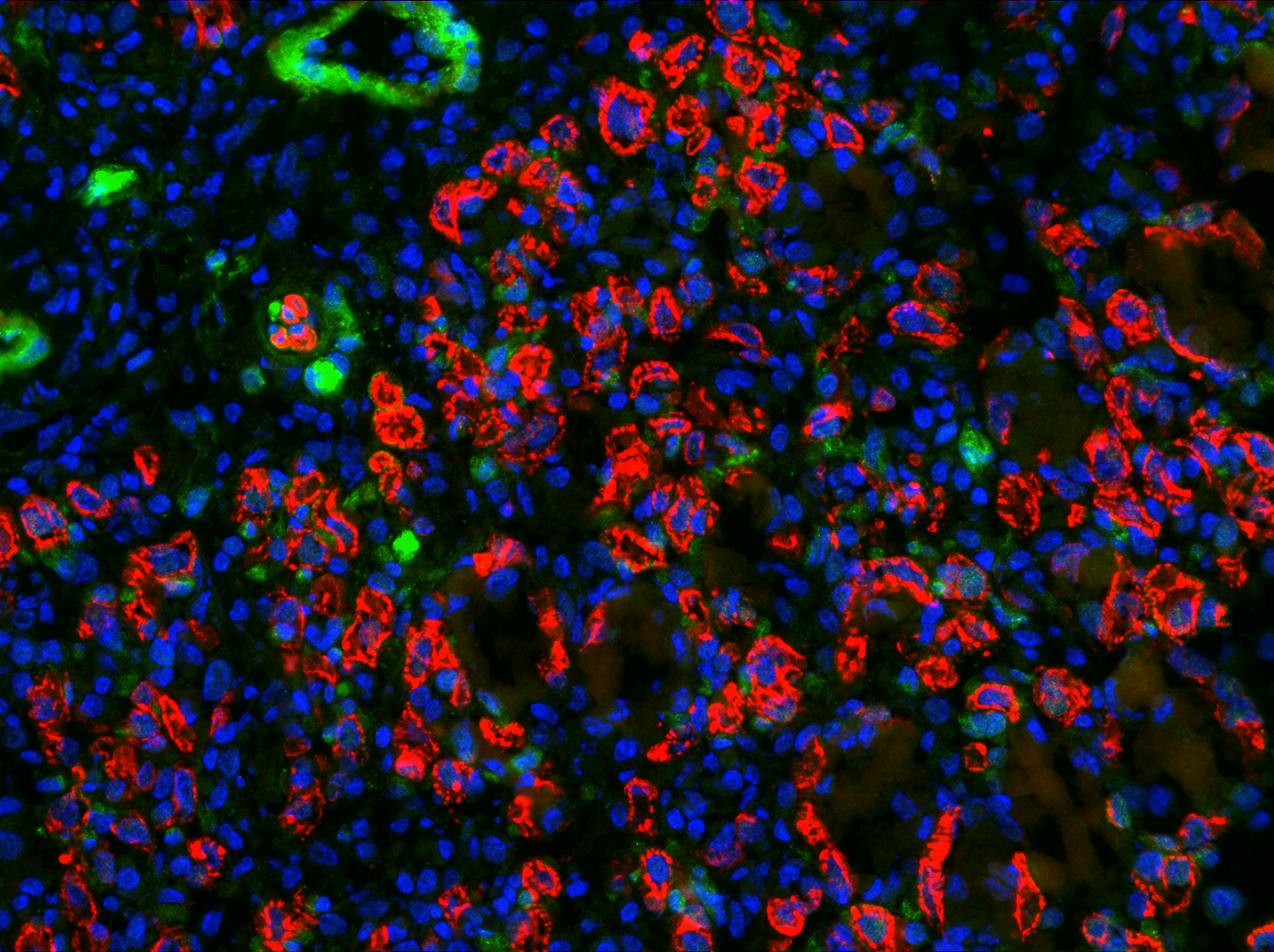

Supplement: Supplementary file 4 — Source data Fig. 2 [file 44318_2025_397_MOESM4_ESM.zip › Figure 2/Figure 2E/IF_eMyHC+Dystrophin_D3.5_WT_TA.tif]

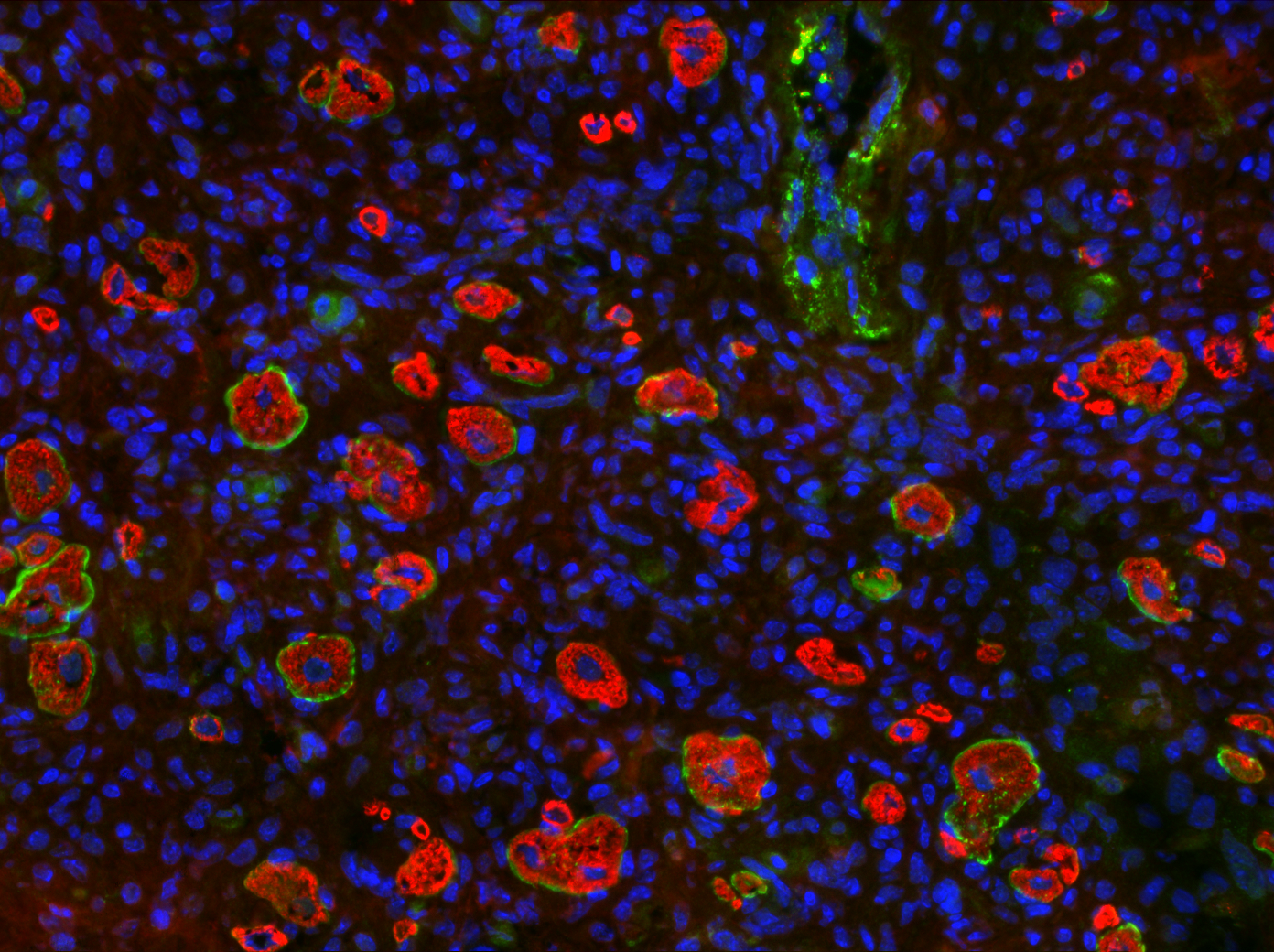

Supplement: Supplementary file 4 — Source data Fig. 2 [file 44318_2025_397_MOESM4_ESM.zip › Figure 2/Figure 2E/IF_eMyHC+Dystrophin_D5.5_KO_TA.tif]

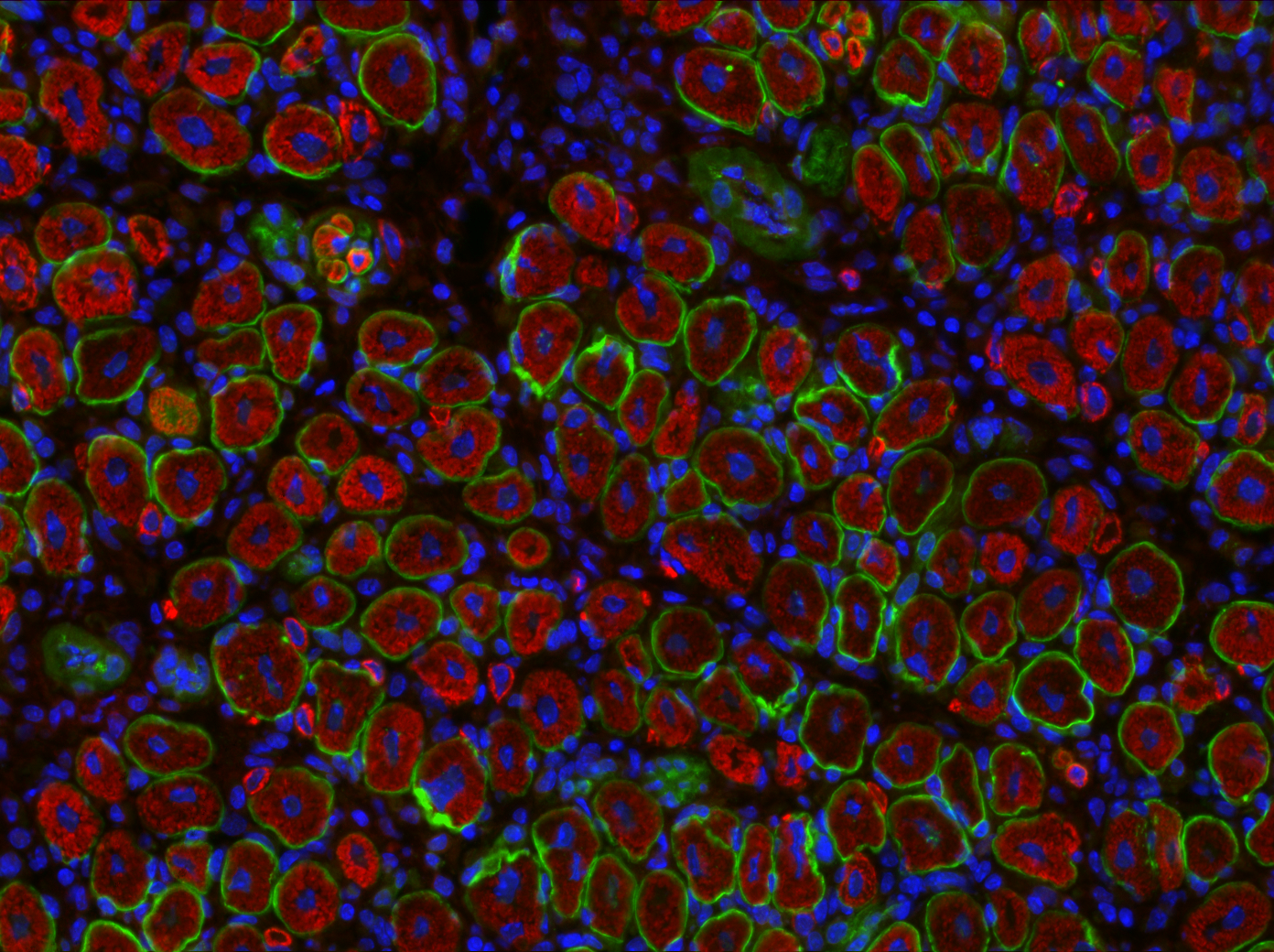

Supplement: Supplementary file 4 — Source data Fig. 2 [file 44318_2025_397_MOESM4_ESM.zip › Figure 2/Figure 2E/IF_eMyHC+Dystrophin_D5.5_WT_TA.tif]

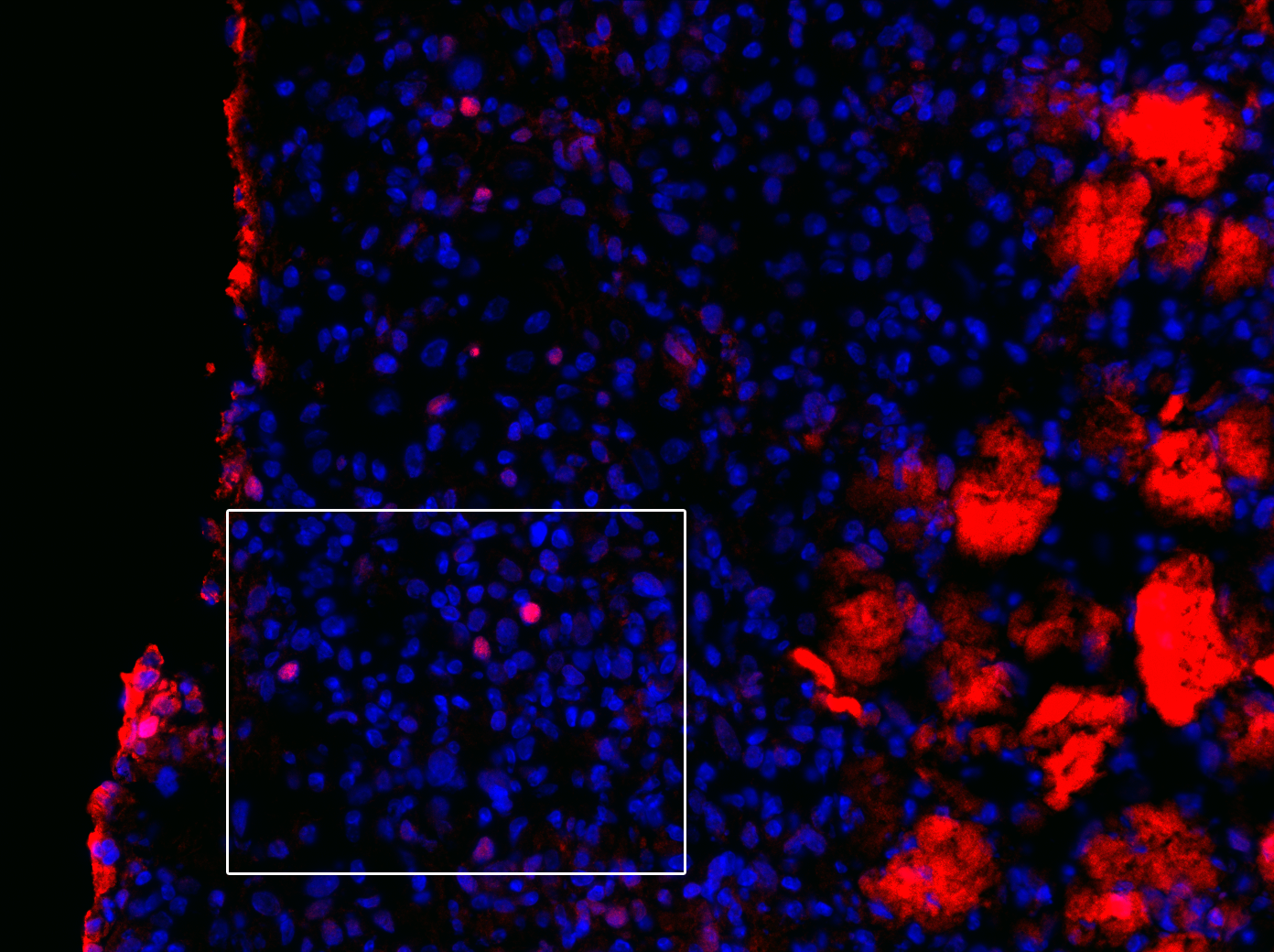

Supplement: Supplementary file 5 — Source data Fig. 3 [file 44318_2025_397_MOESM5_ESM.zip › Figure 3/Figure 3A/IF_Pax7_D3.5_KO_TA.tif]

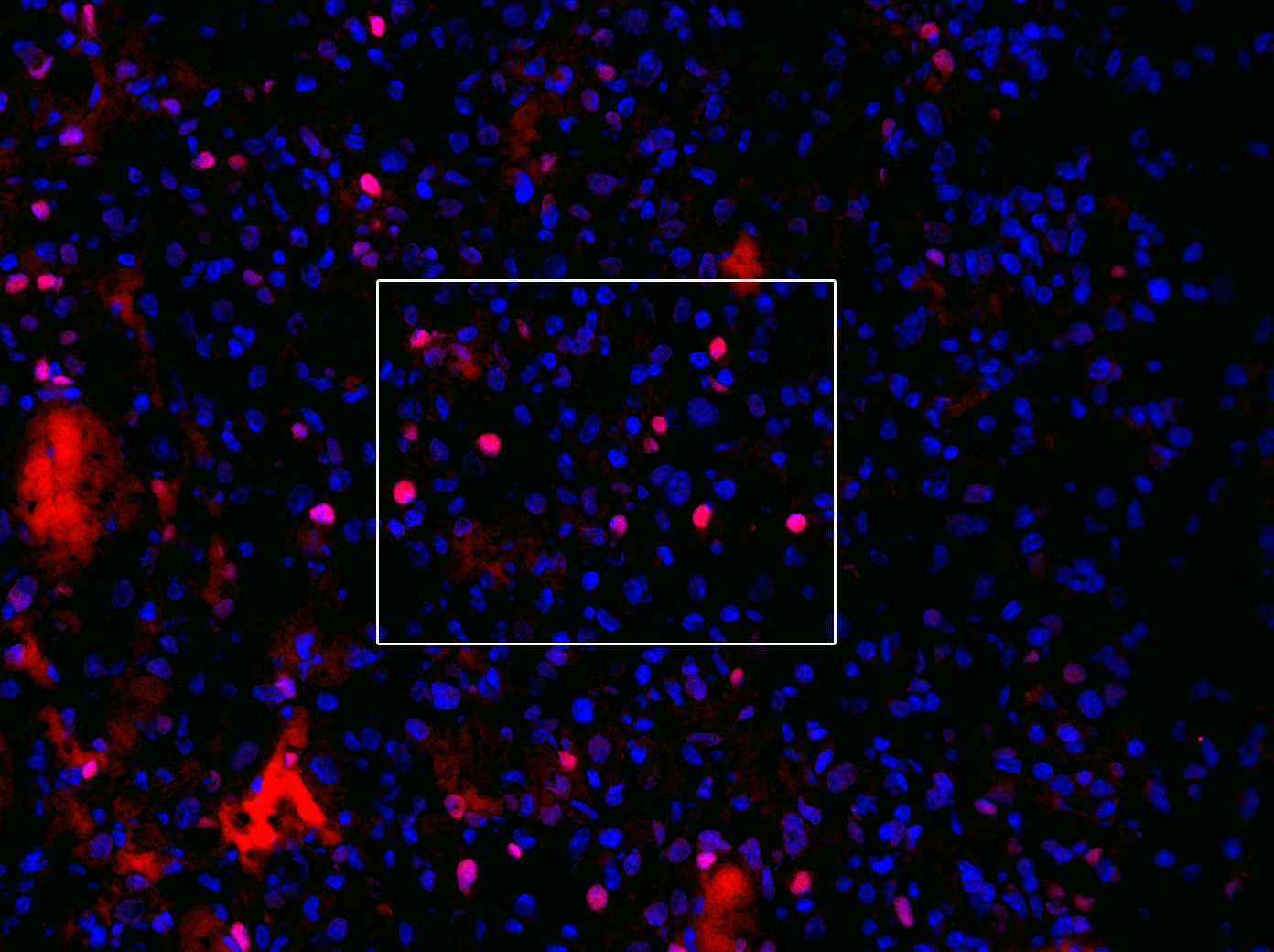

Supplement: Supplementary file 5 — Source data Fig. 3 [file 44318_2025_397_MOESM5_ESM.zip › Figure 3/Figure 3A/IF_Pax7_D3.5_WT_TA.tif]

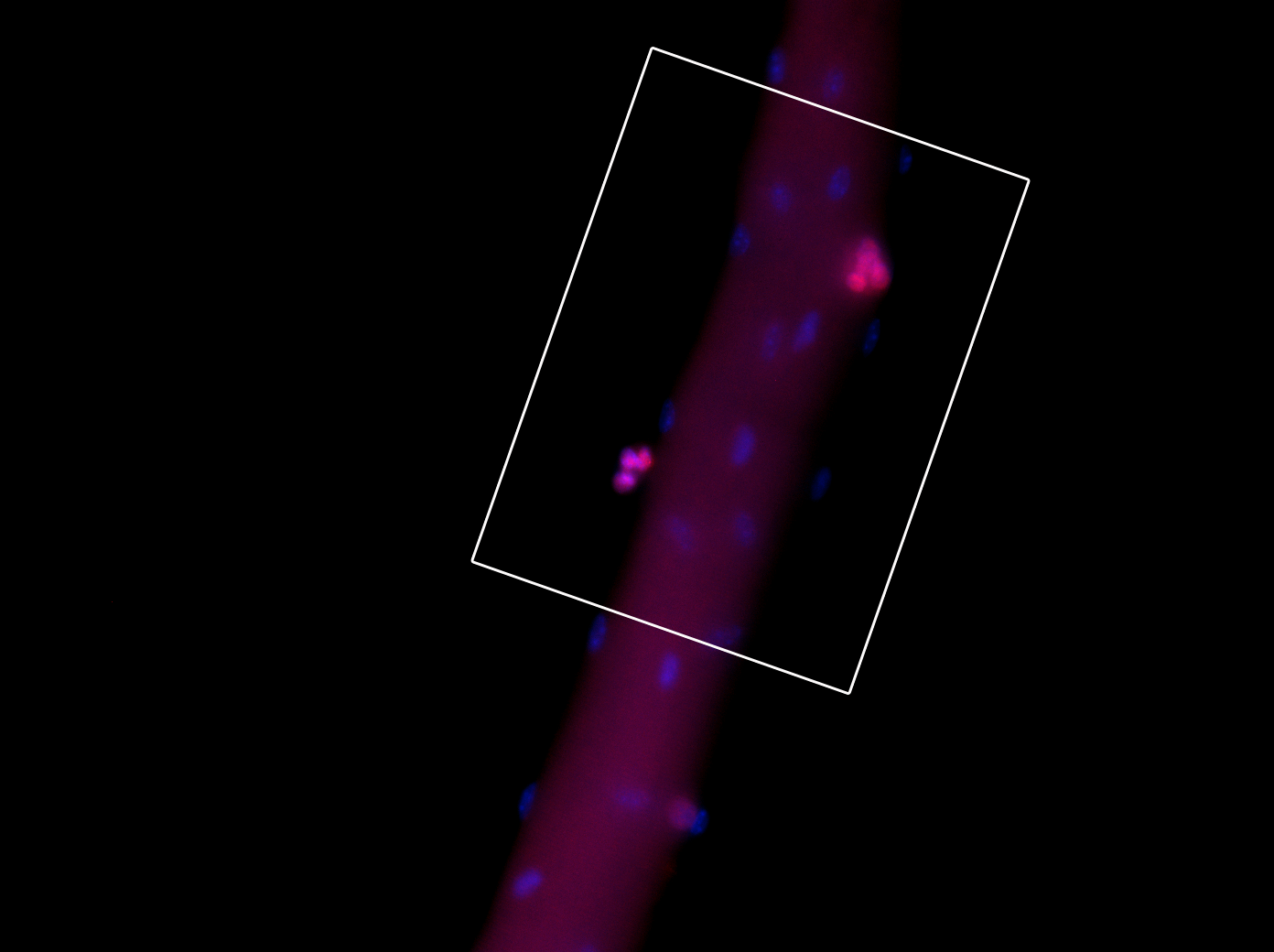

Supplement: Supplementary file 5 — Source data Fig. 3 [file 44318_2025_397_MOESM5_ESM.zip › Figure 3/Figure 3C/IF_Pax7_Fiber72h_KO.tif]

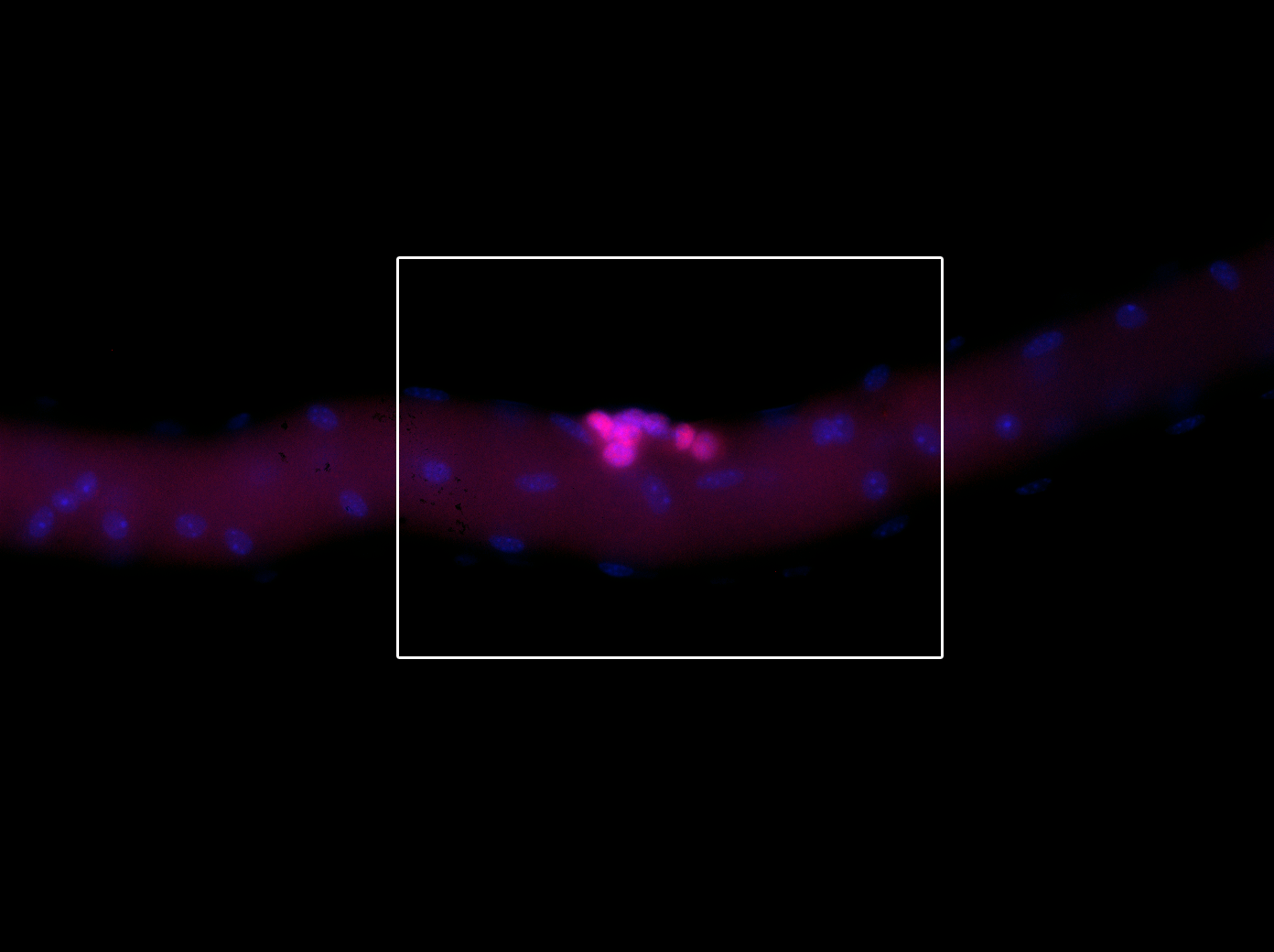

Supplement: Supplementary file 5 — Source data Fig. 3 [file 44318_2025_397_MOESM5_ESM.zip › Figure 3/Figure 3C/IF_Pax7_Fiber72h_WT.tif]

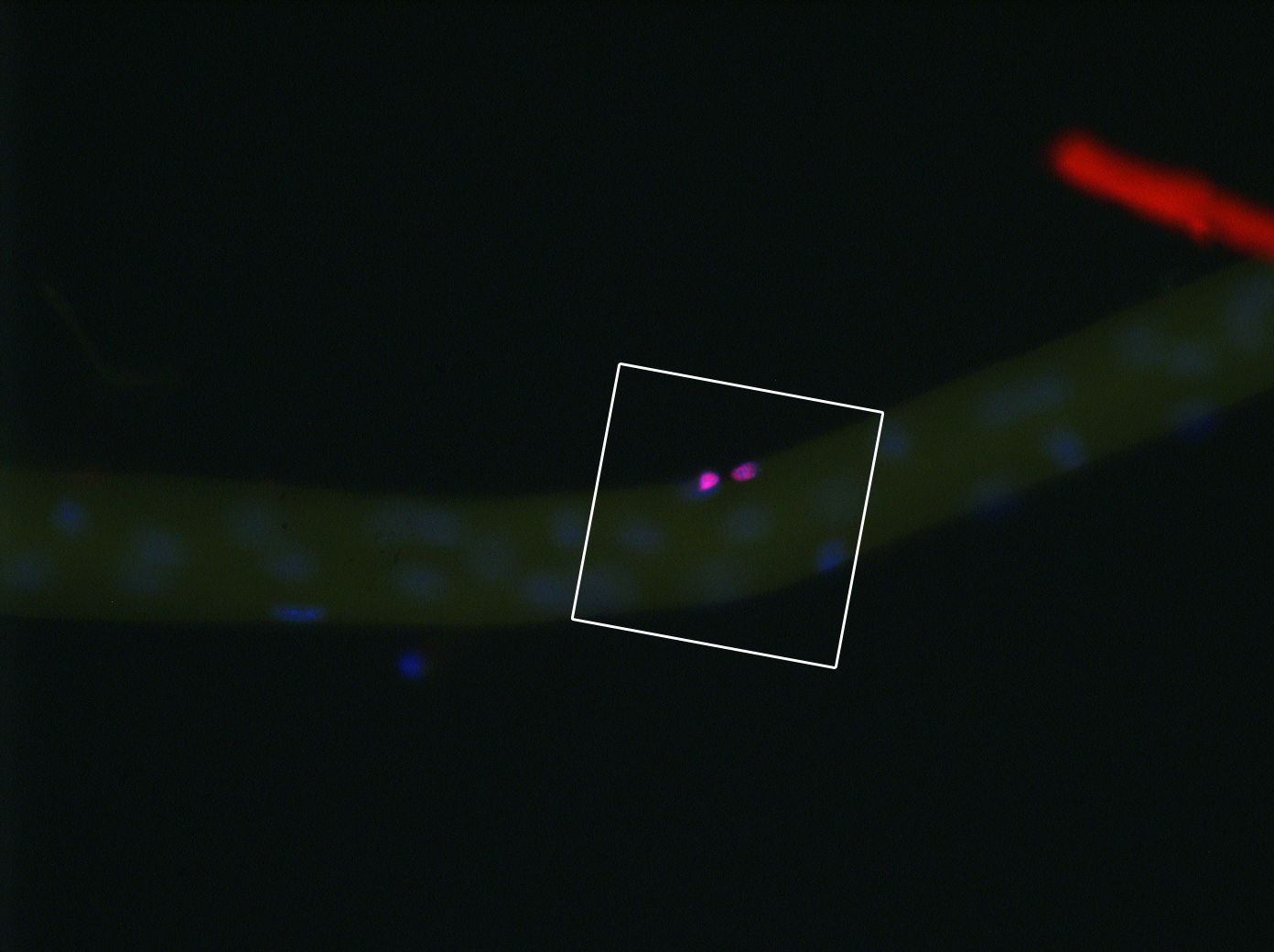

Supplement: Supplementary file 5 — Source data Fig. 3 [file 44318_2025_397_MOESM5_ESM.zip › Figure 3/Figure 3E/IF_Pax7+TUNEL_Fiber_40h_KO.tif]

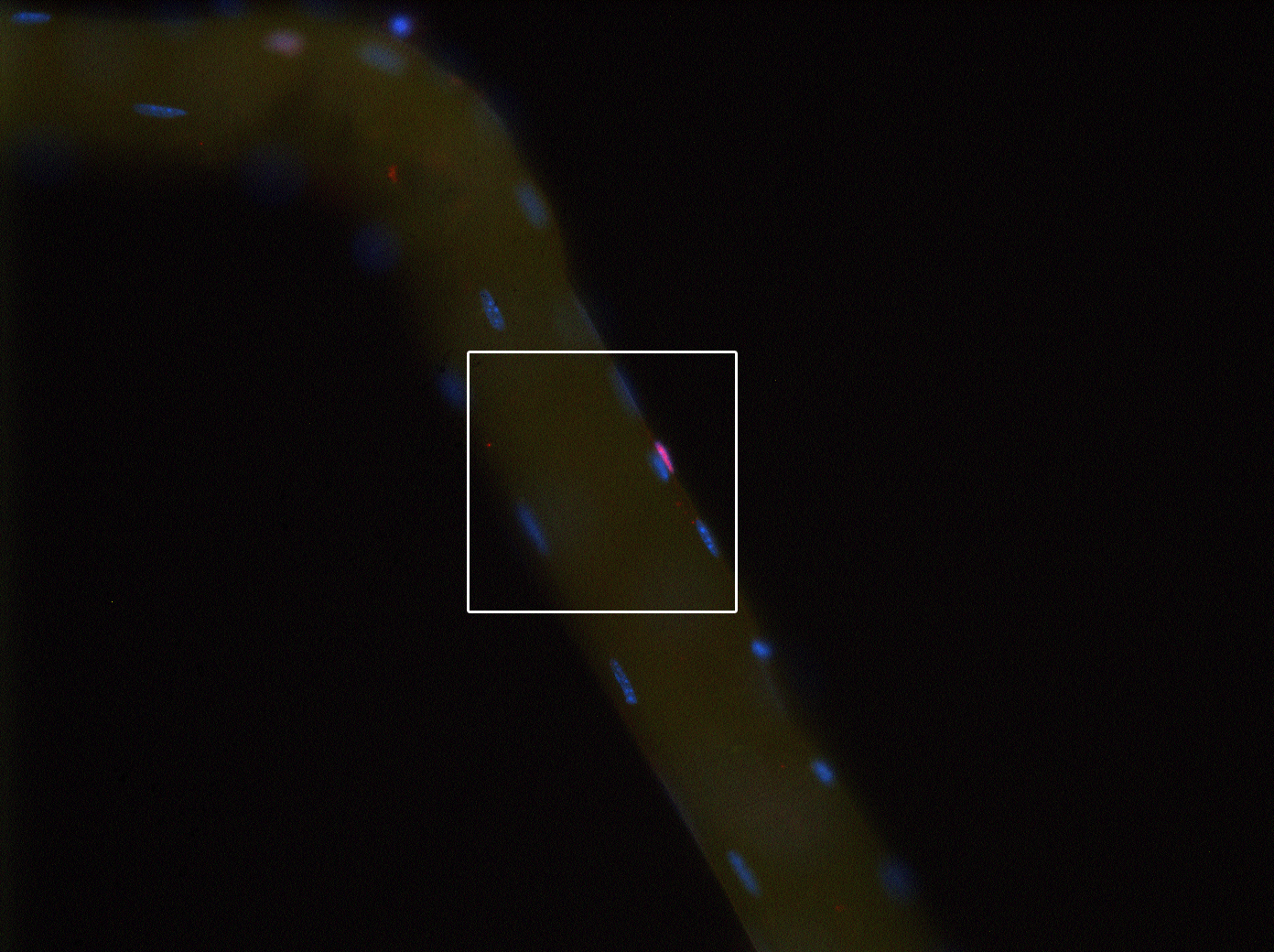

Supplement: Supplementary file 5 — Source data Fig. 3 [file 44318_2025_397_MOESM5_ESM.zip › Figure 3/Figure 3E/IF_Pax7+TUNEL_Fiber_40h_NC.tif]

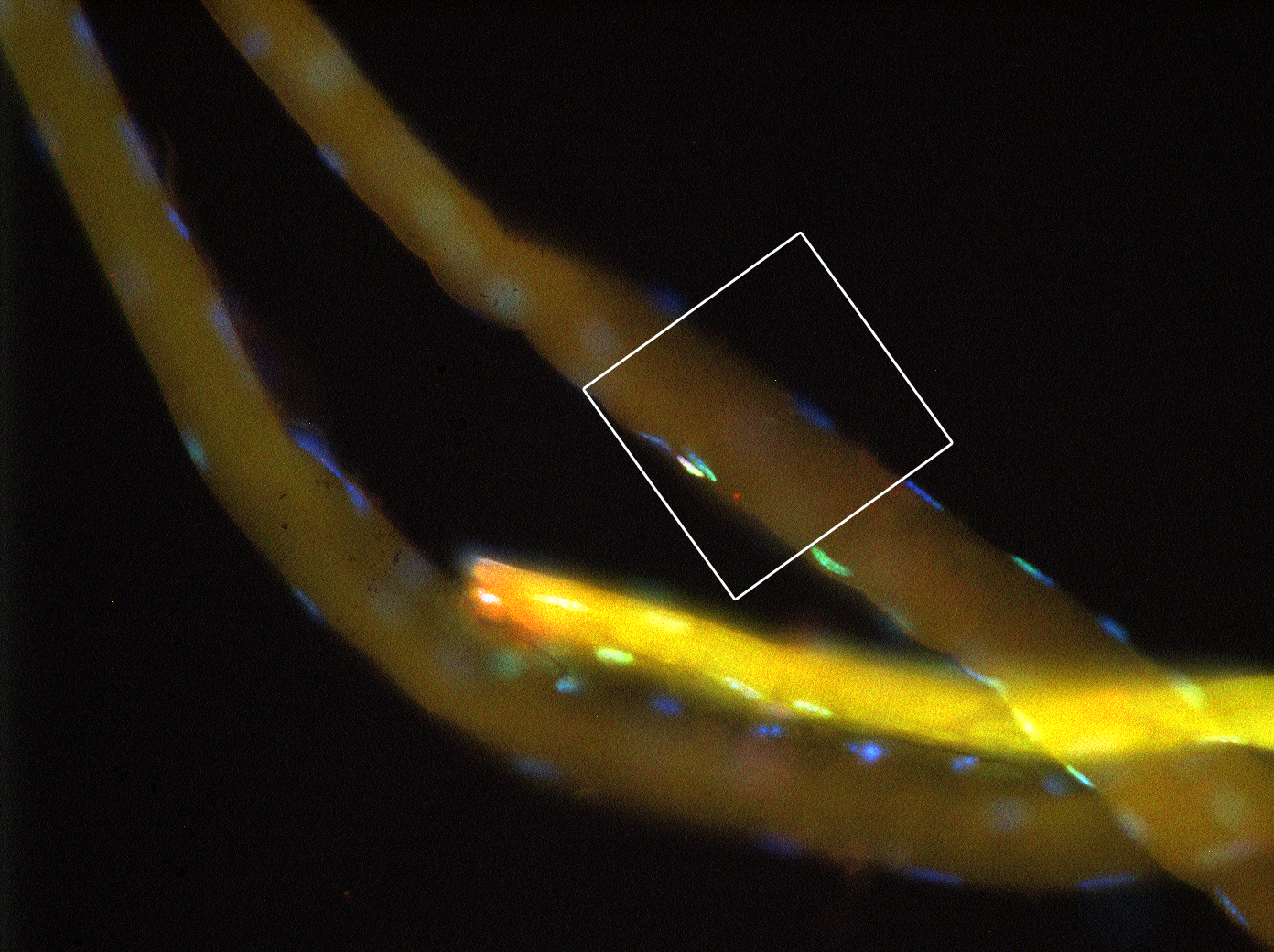

Supplement: Supplementary file 5 — Source data Fig. 3 [file 44318_2025_397_MOESM5_ESM.zip › Figure 3/Figure 3E/IF_Pax7+TUNEL_Fiber_40h_PC.tif]

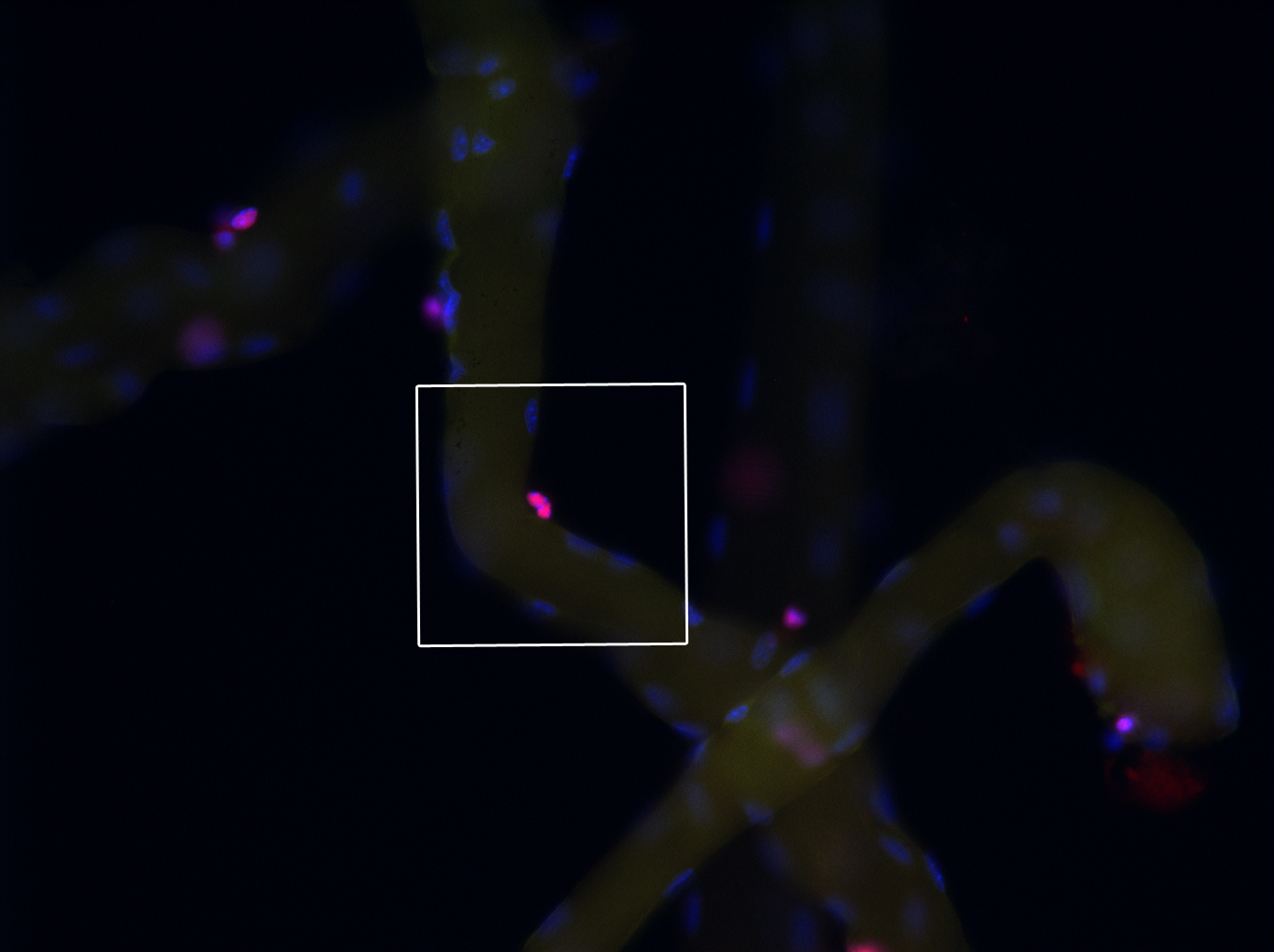

Supplement: Supplementary file 5 — Source data Fig. 3 [file 44318_2025_397_MOESM5_ESM.zip › Figure 3/Figure 3E/IF_Pax7+TUNEL_Fiber_40h_WT.tif]

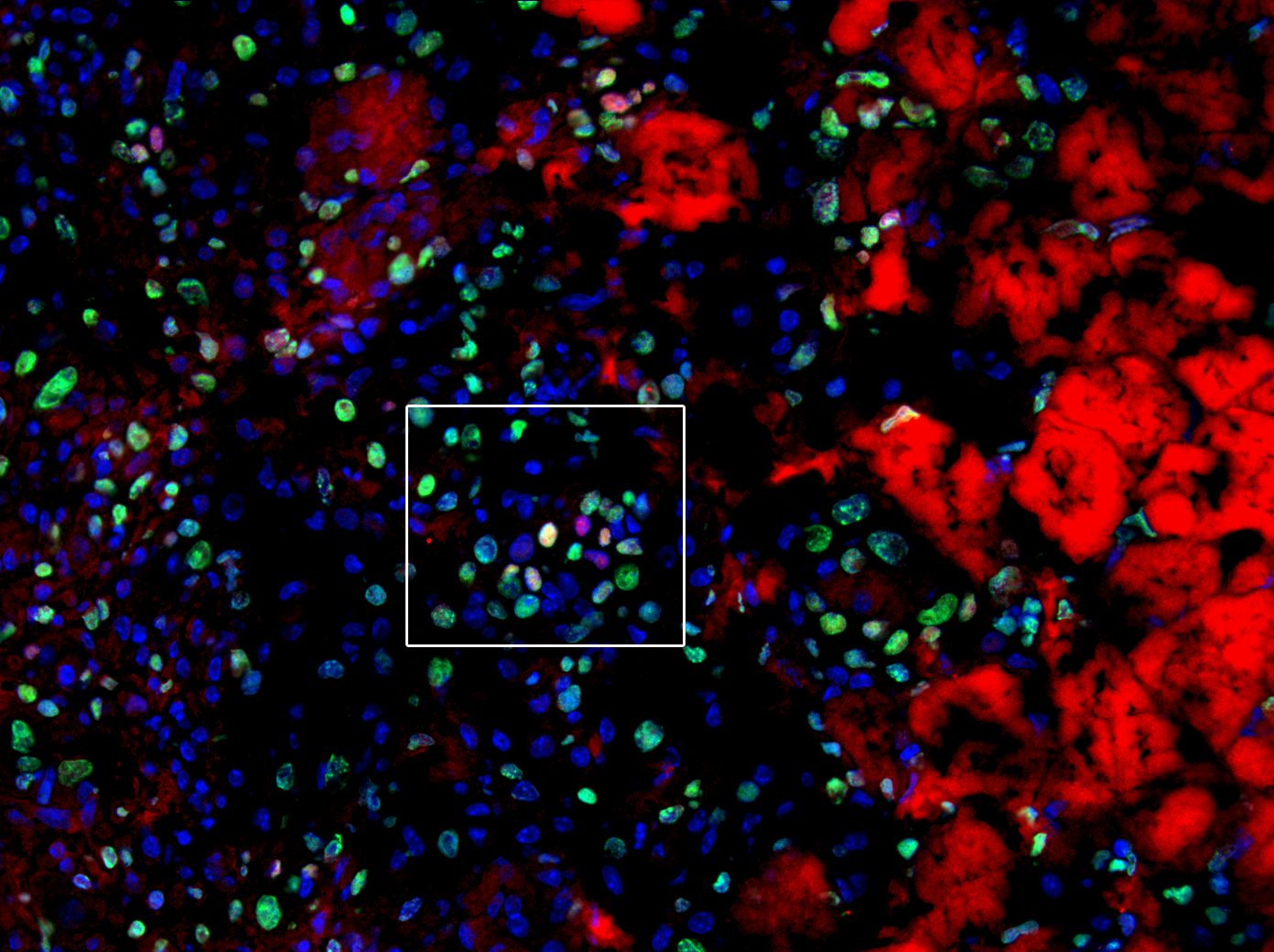

Supplement: Supplementary file 5 — Source data Fig. 3 [file 44318_2025_397_MOESM5_ESM.zip › Figure 3/Figure 3G/IF_Pax7+EdU_D3.5_KO_TA.tif]

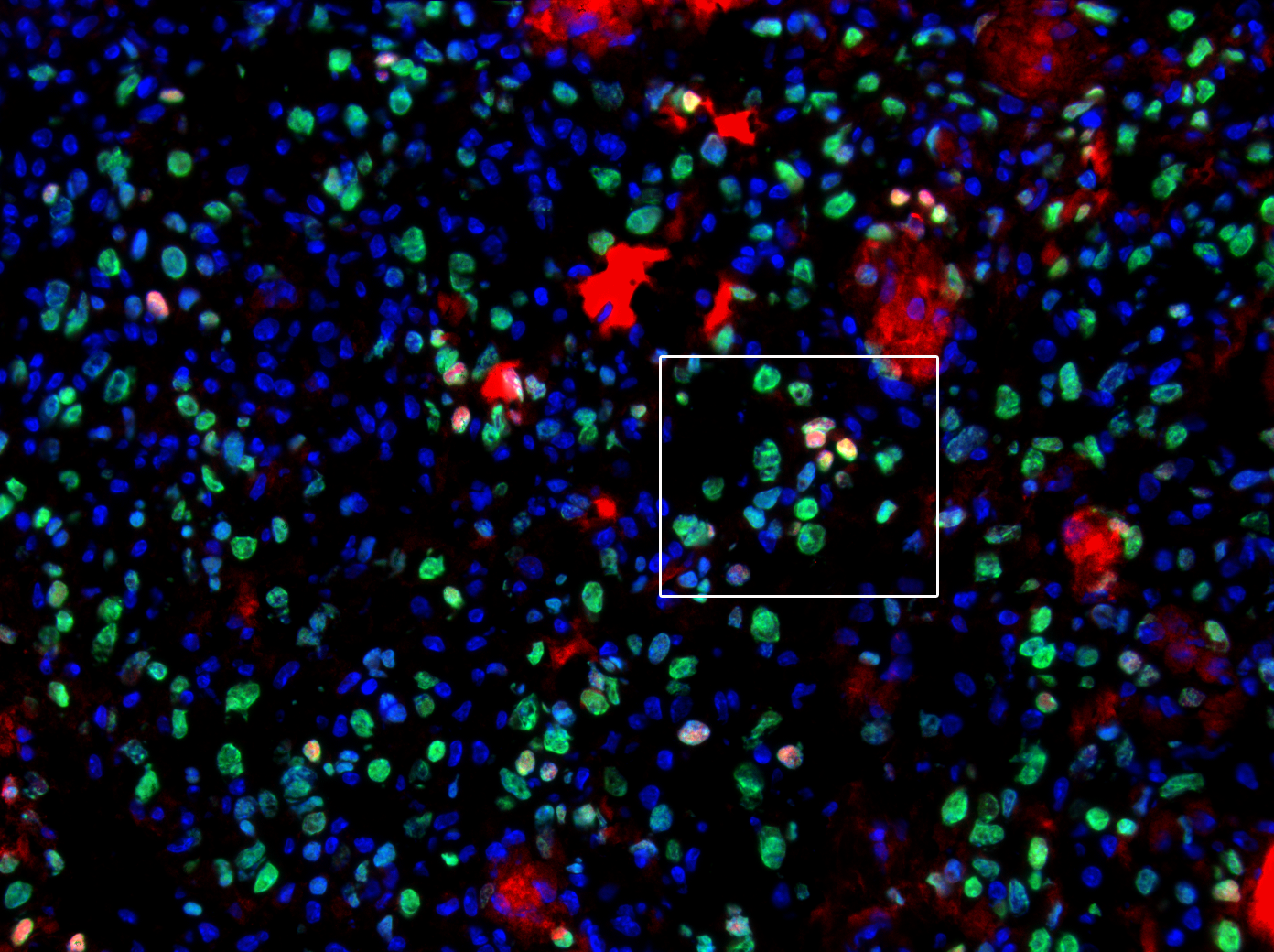

Supplement: Supplementary file 5 — Source data Fig. 3 [file 44318_2025_397_MOESM5_ESM.zip › Figure 3/Figure 3G/IF_Pax7+EdU_D3.5_WT_TA.tif]

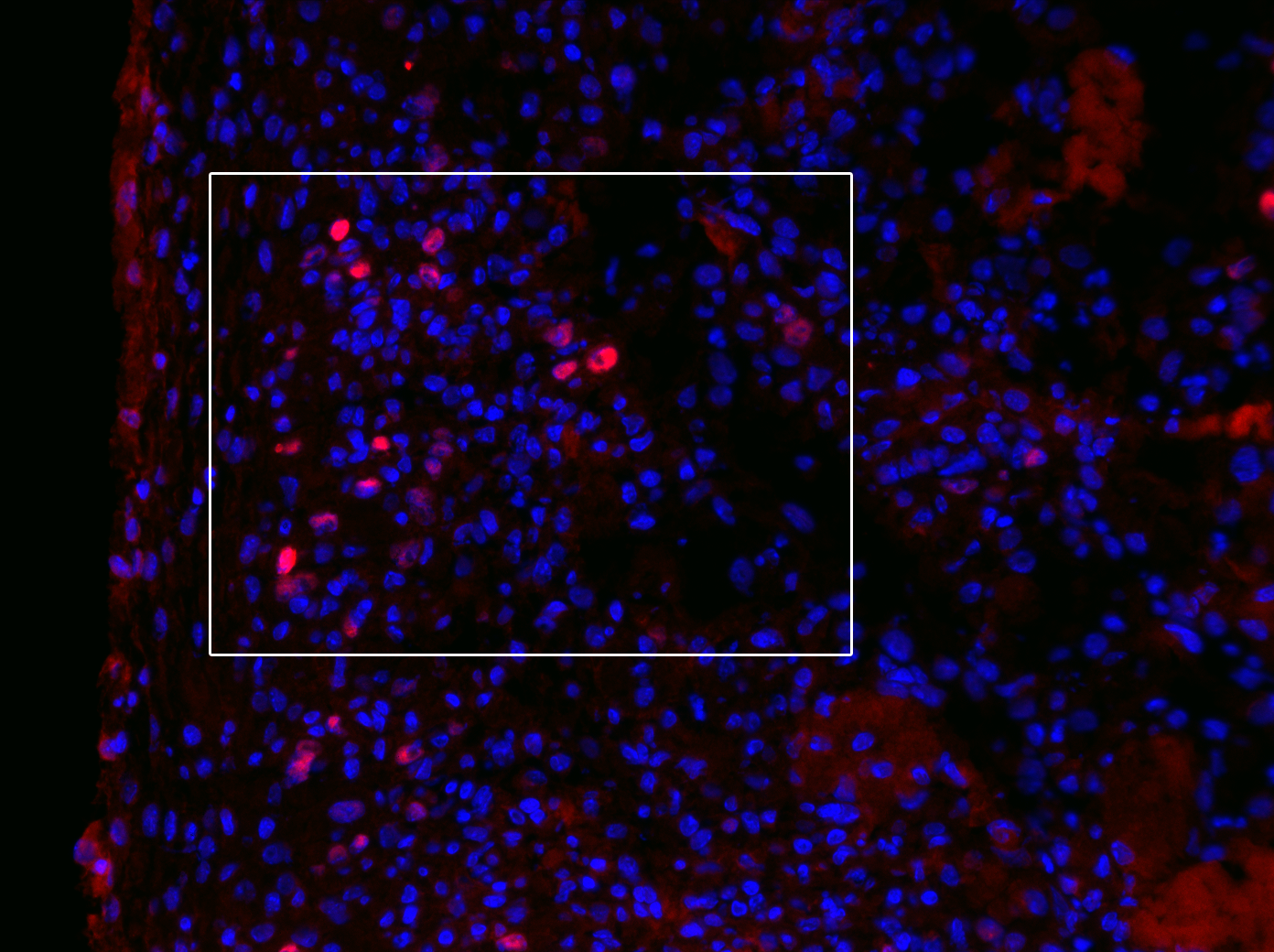

Supplement: Supplementary file 5 — Source data Fig. 3 [file 44318_2025_397_MOESM5_ESM.zip › Figure 3/Figure 3I/IF_MyoG_D3.5_KO_TA.tif]

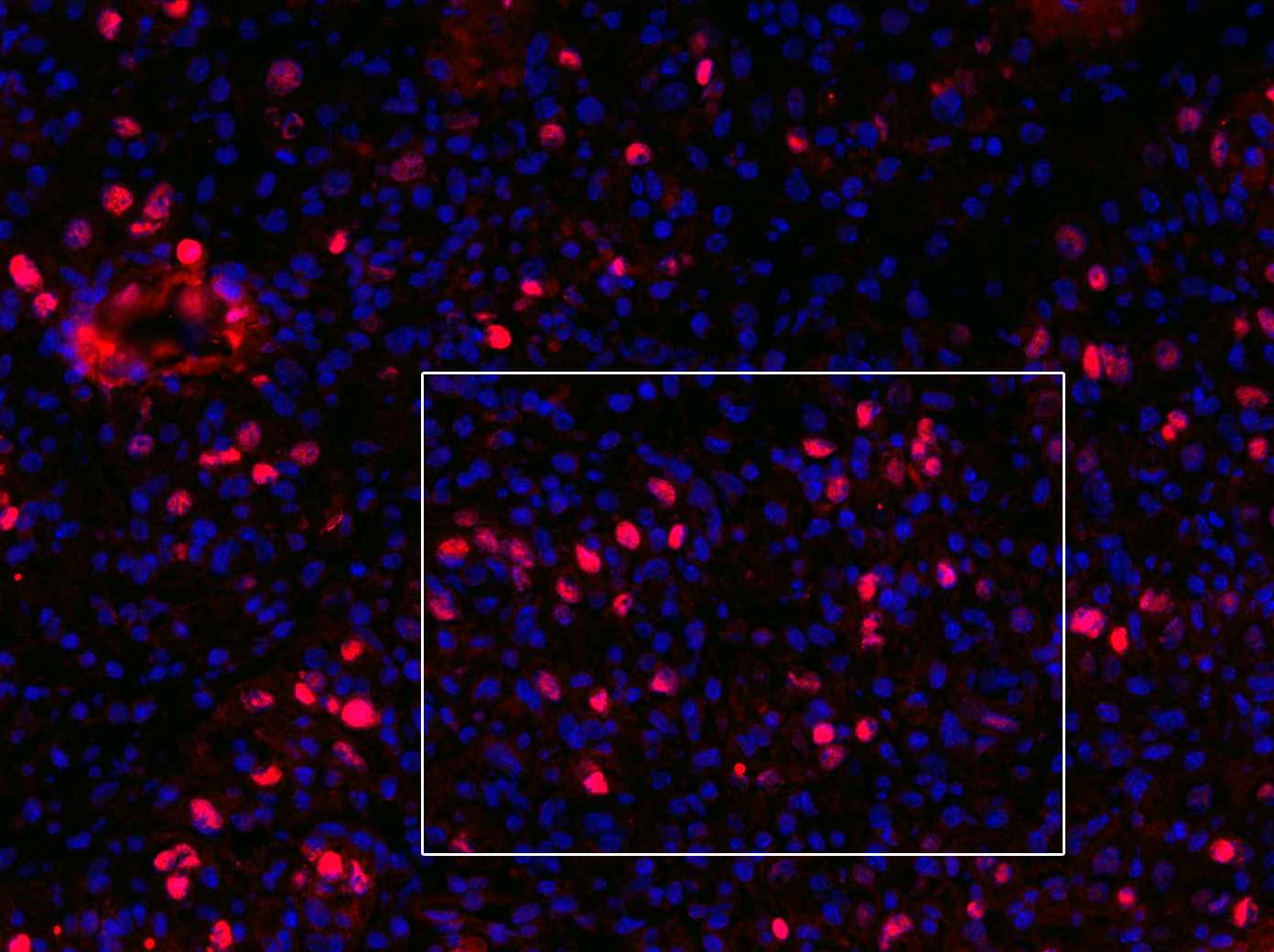

Supplement: Supplementary file 5 — Source data Fig. 3 [file 44318_2025_397_MOESM5_ESM.zip › Figure 3/Figure 3I/IF_MyoG_D3.5_WT_TA.tif]

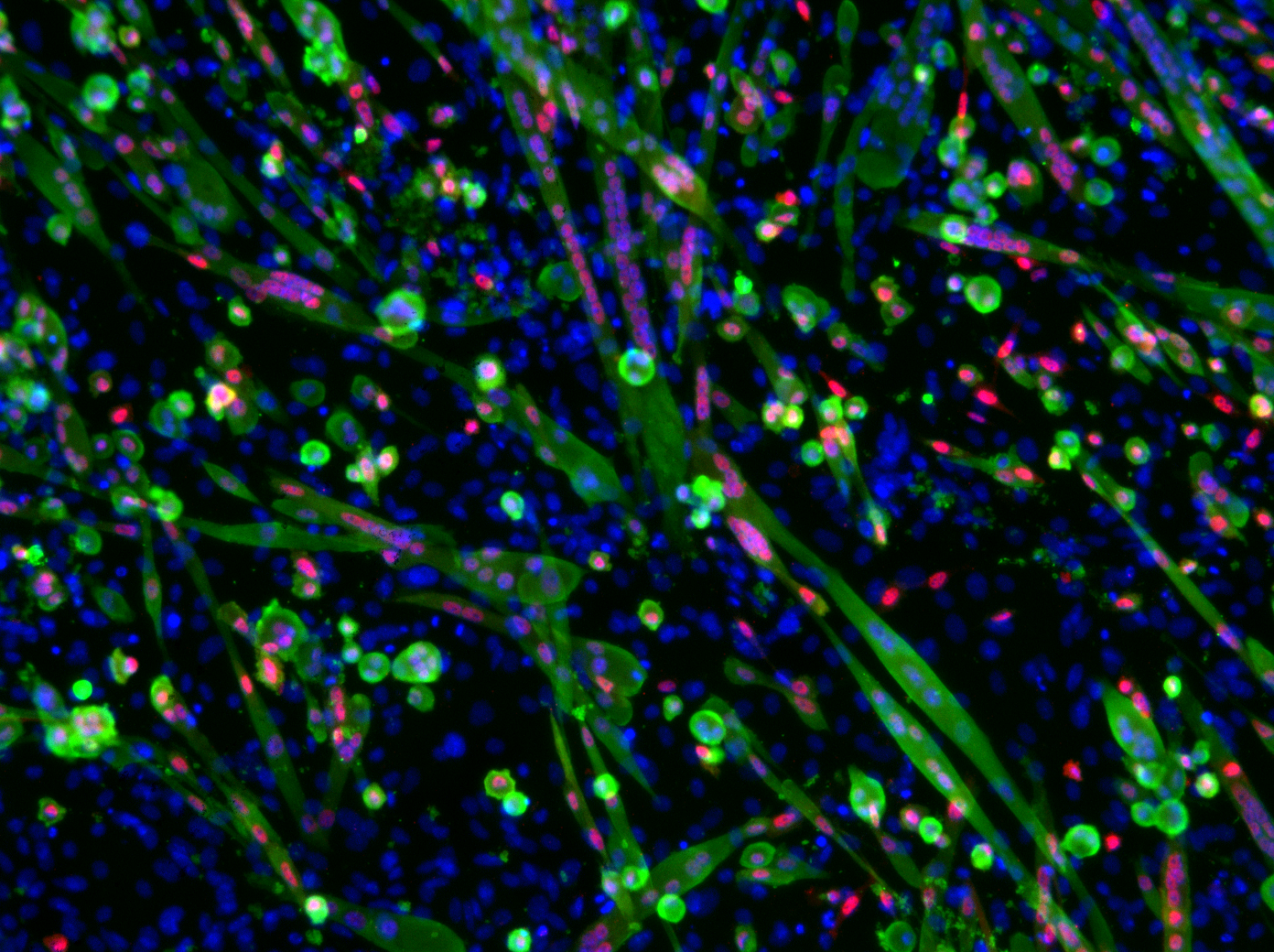

Supplement: Supplementary file 5 — Source data Fig. 3 [file 44318_2025_397_MOESM5_ESM.zip › Figure 3/Figure 3K/IF_MyoG+MF20_MyoblastDifferentiation_2d_KO.tif]

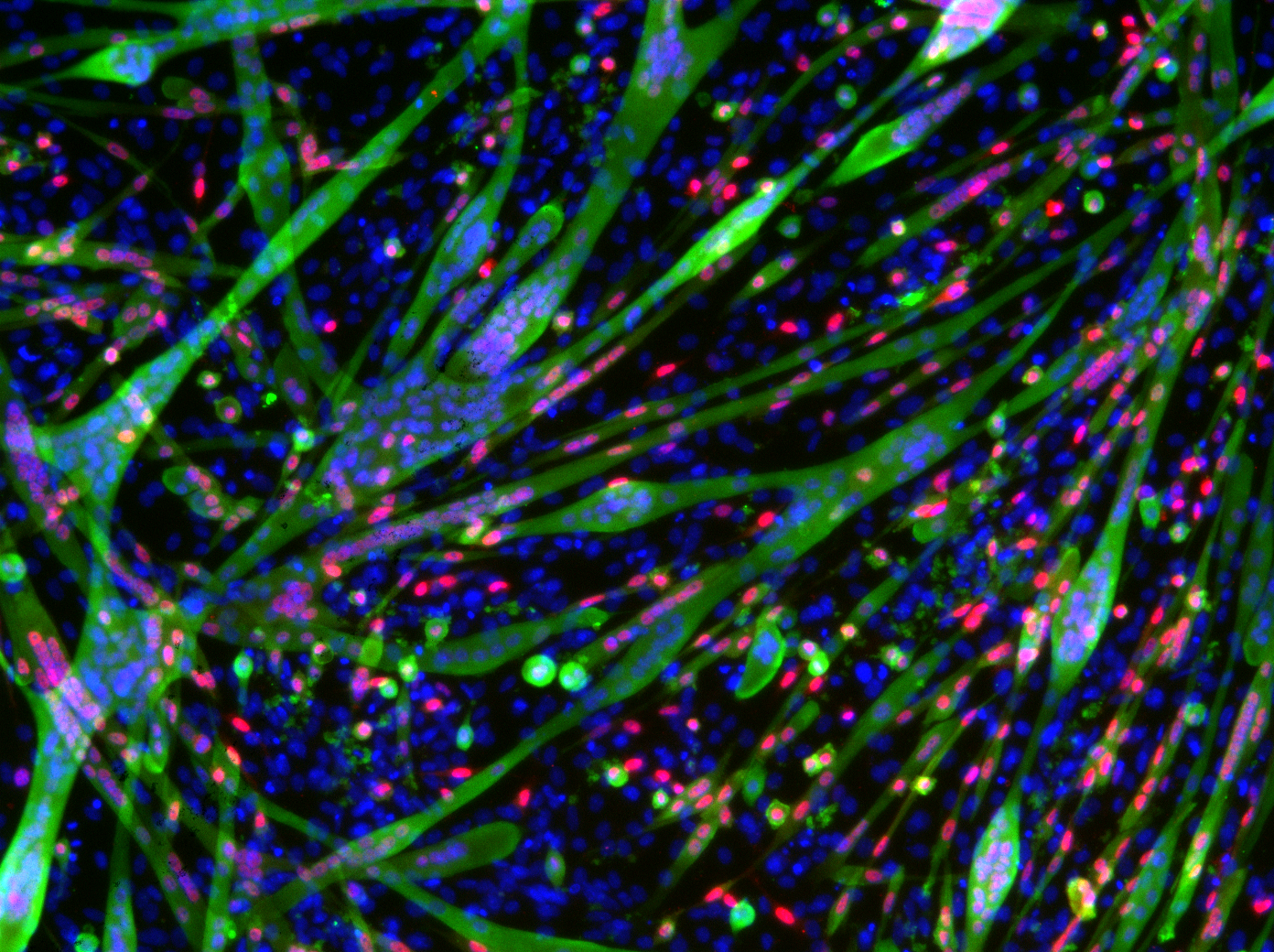

Supplement: Supplementary file 5 — Source data Fig. 3 [file 44318_2025_397_MOESM5_ESM.zip › Figure 3/Figure 3K/IF_MyoG+MF20_MyoblastDifferentiation_2d_WT.tif]

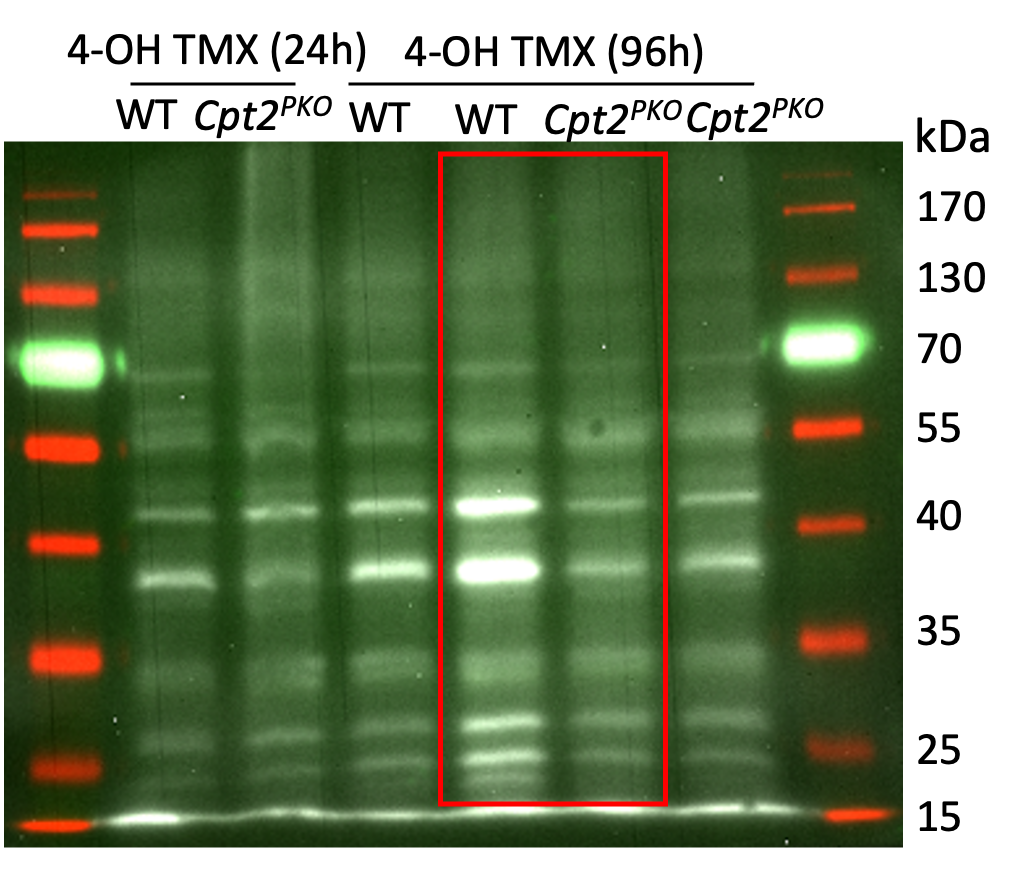

Supplement: Supplementary file 7 — Source data Fig. 5 [file 44318_2025_397_MOESM7_ESM.zip › Figure 5/Figure 5B/5B. Acetylated-lysine.tiff]

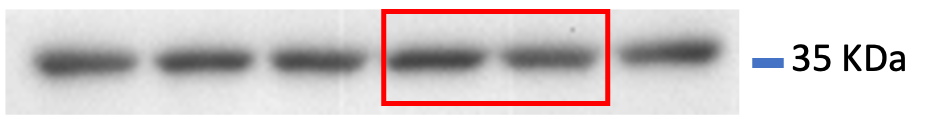

Supplement: Supplementary file 7 — Source data Fig. 5 [file 44318_2025_397_MOESM7_ESM.zip › Figure 5/Figure 5B/5B. GAPDH.tiff]

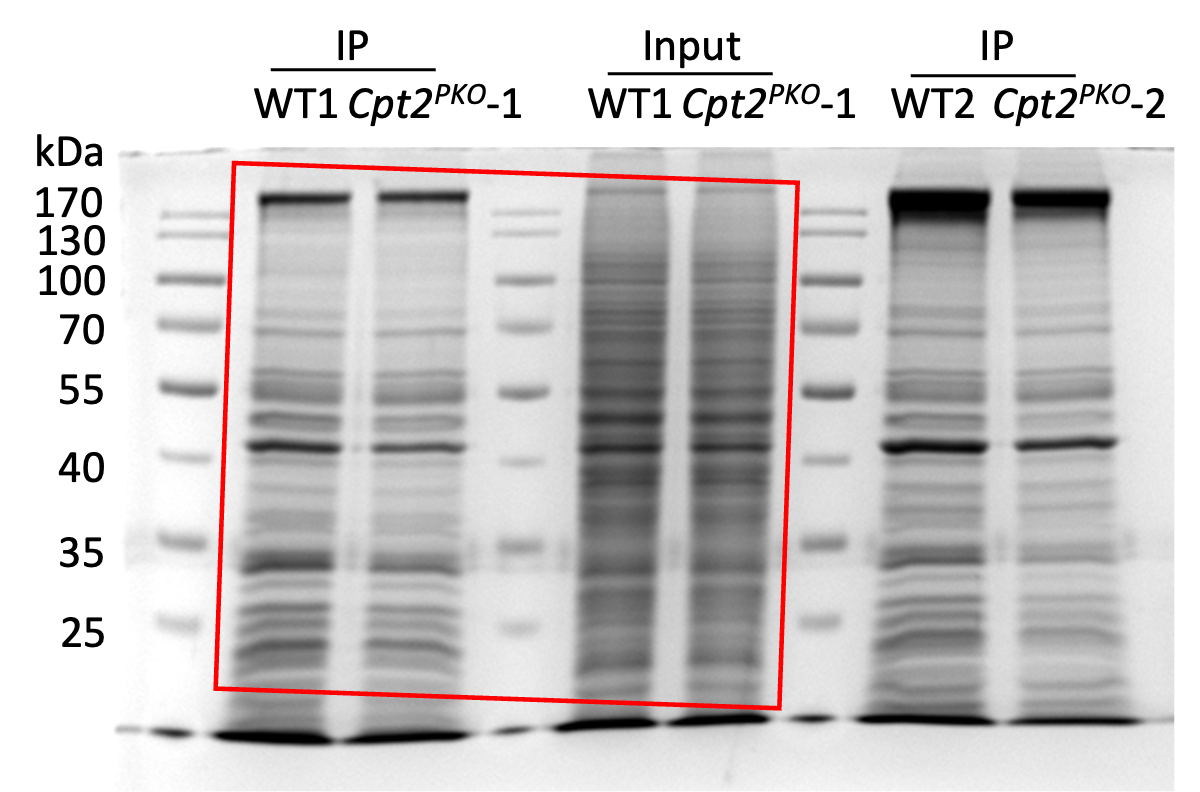

Supplement: Supplementary file 7 — Source data Fig. 5 [file 44318_2025_397_MOESM7_ESM.zip › Figure 5/Figure 5C/5C. Coomssie Blue.tiff]

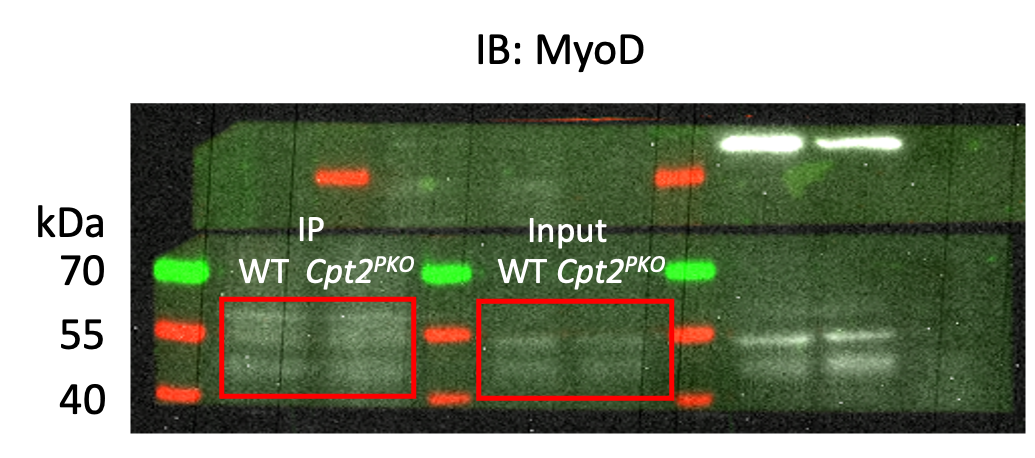

Supplement: Supplementary file 7 — Source data Fig. 5 [file 44318_2025_397_MOESM7_ESM.zip › Figure 5/Figure 5D/5D. MyoD.tiff]

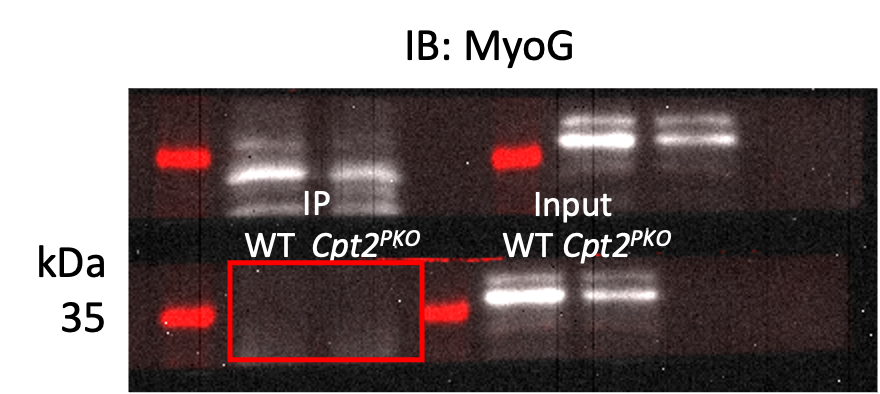

Supplement: Supplementary file 7 — Source data Fig. 5 [file 44318_2025_397_MOESM7_ESM.zip › Figure 5/Figure 5D/5D. MyoG.tiff]

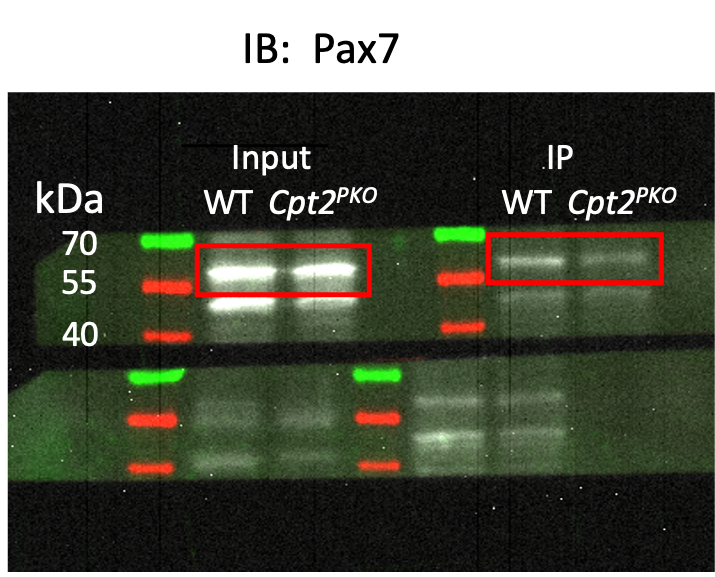

Supplement: Supplementary file 7 — Source data Fig. 5 [file 44318_2025_397_MOESM7_ESM.zip › Figure 5/Figure 5D/5D. Pax7.tiff]

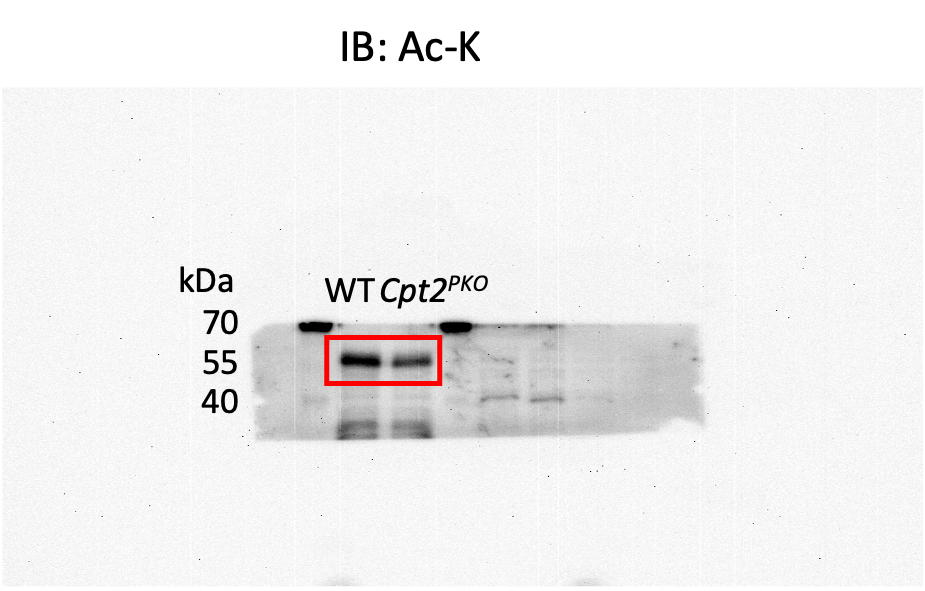

Supplement: Supplementary file 7 — Source data Fig. 5 [file 44318_2025_397_MOESM7_ESM.zip › Figure 5/Figure 5E/5E. Acetylated-Lysine.tiff]

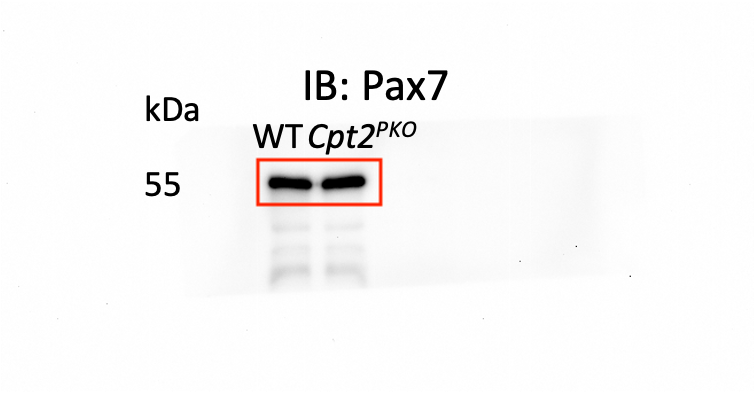

Supplement: Supplementary file 7 — Source data Fig. 5 [file 44318_2025_397_MOESM7_ESM.zip › Figure 5/Figure 5E/5E. Pax7.tiff]

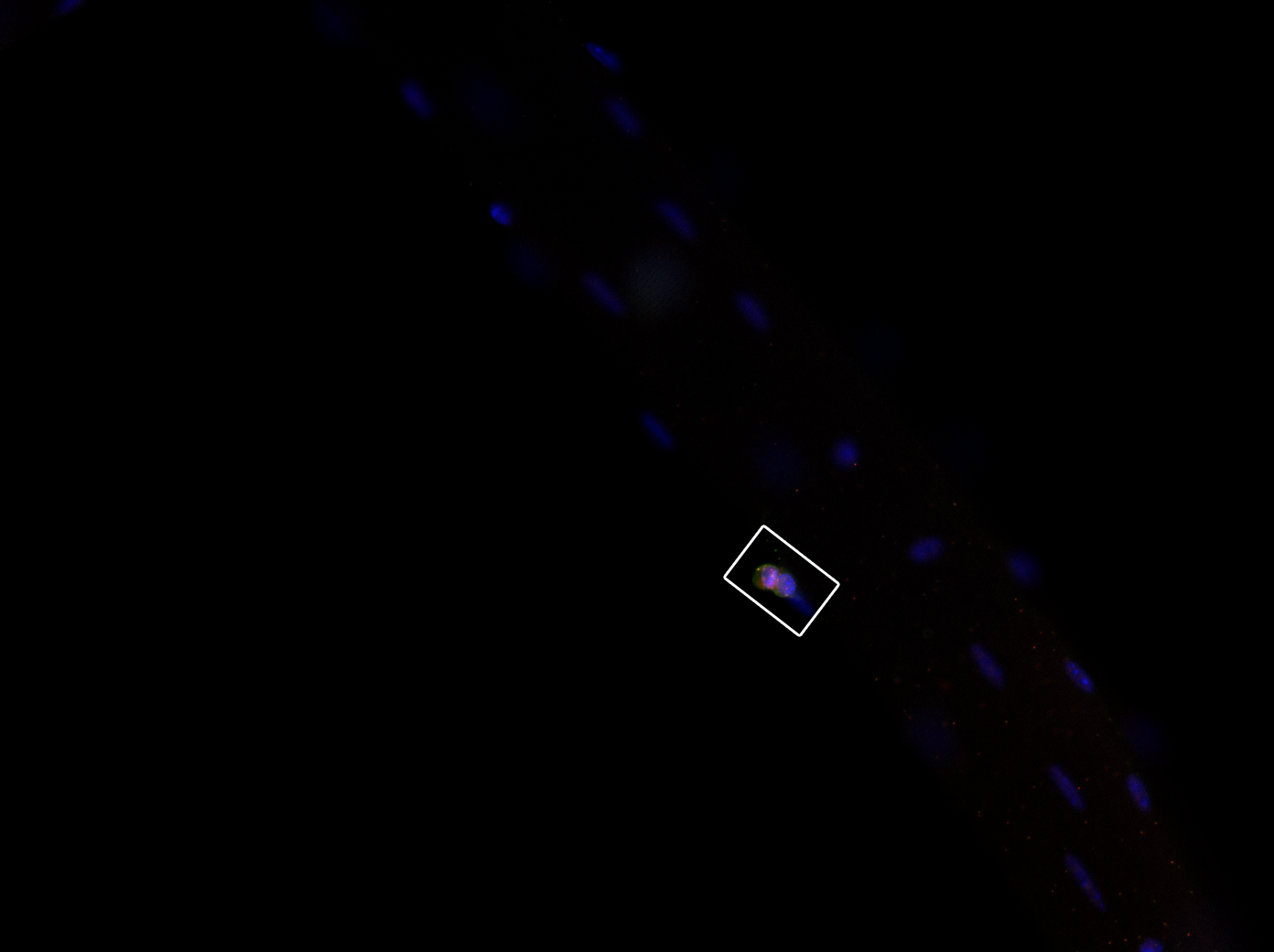

Supplement: Supplementary file 7 — Source data Fig. 5 [file 44318_2025_397_MOESM7_ESM.zip › Figure 5/Figure 5F/PLA_Pax7+Acetylated-lysine_Fiber_48h_KO.tif]

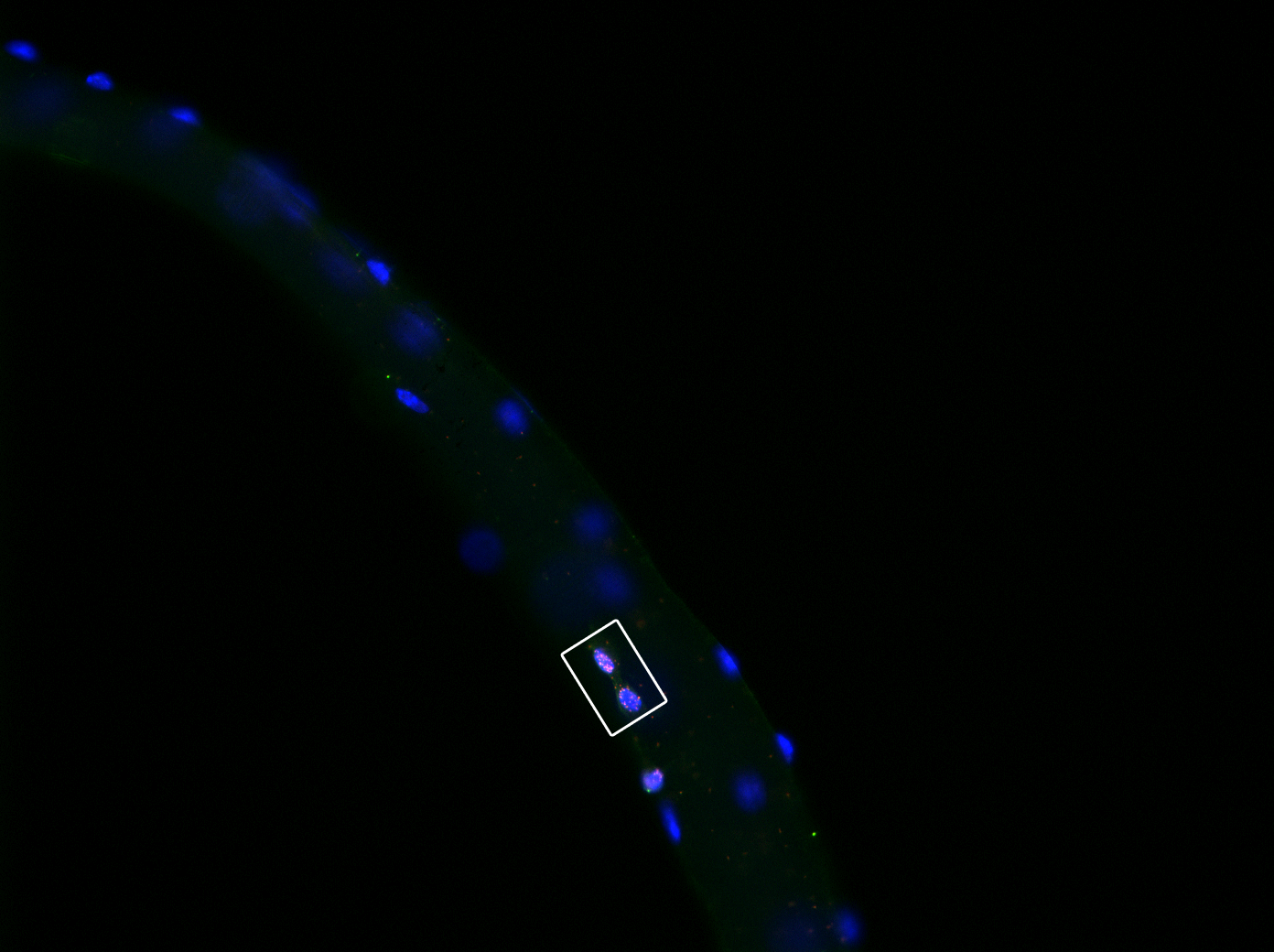

Supplement: Supplementary file 7 — Source data Fig. 5 [file 44318_2025_397_MOESM7_ESM.zip › Figure 5/Figure 5F/PLA_Pax7+Acetylated-lysine_Fiber_48h_WT.tif]

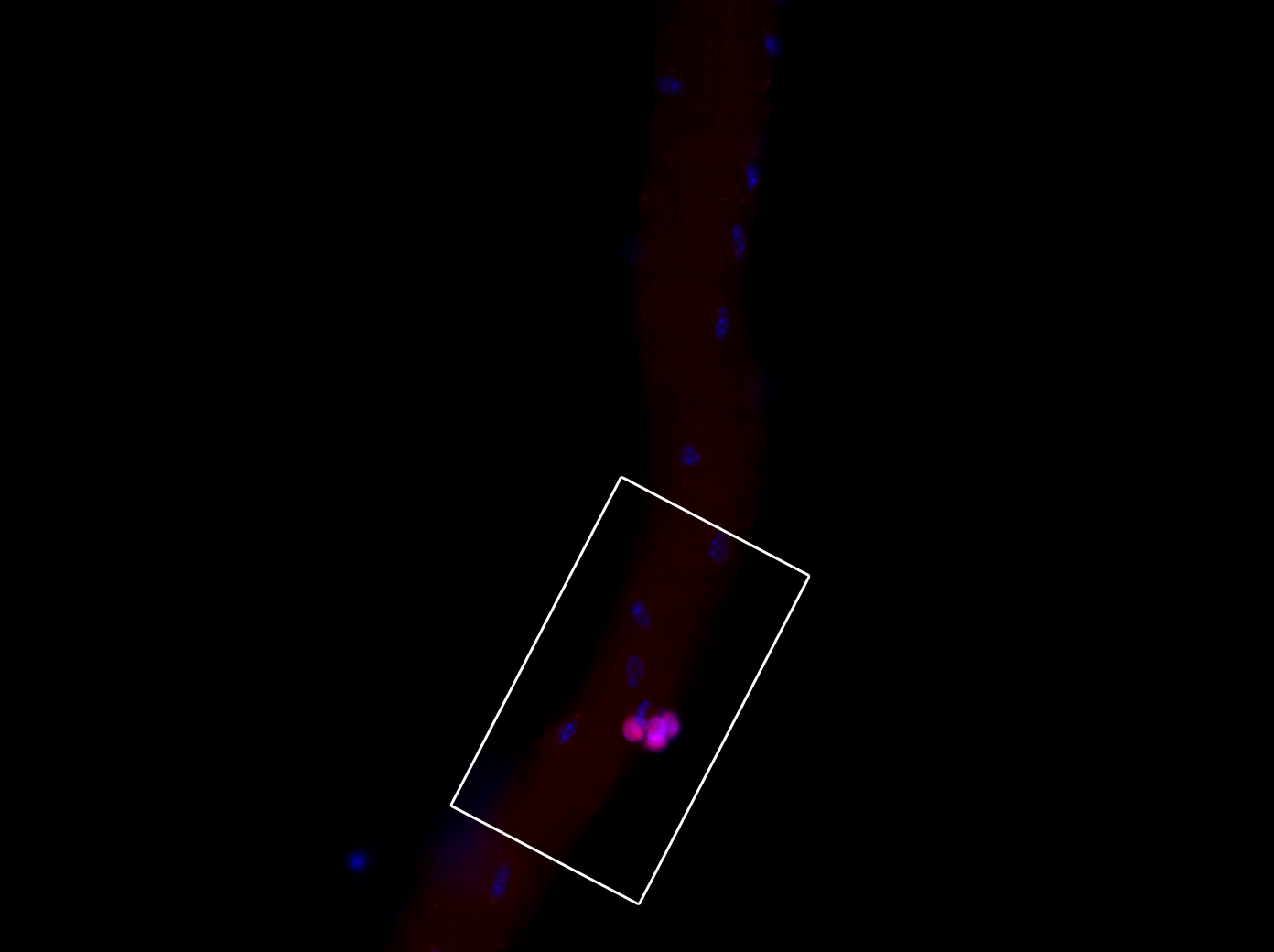

Supplement: Supplementary file 8 — Source data Fig. 6 [file 44318_2025_397_MOESM8_ESM.zip › Figure 6/Figure 6E/IF_Pax7_Fiber 60h_KO+Acetate.tif]

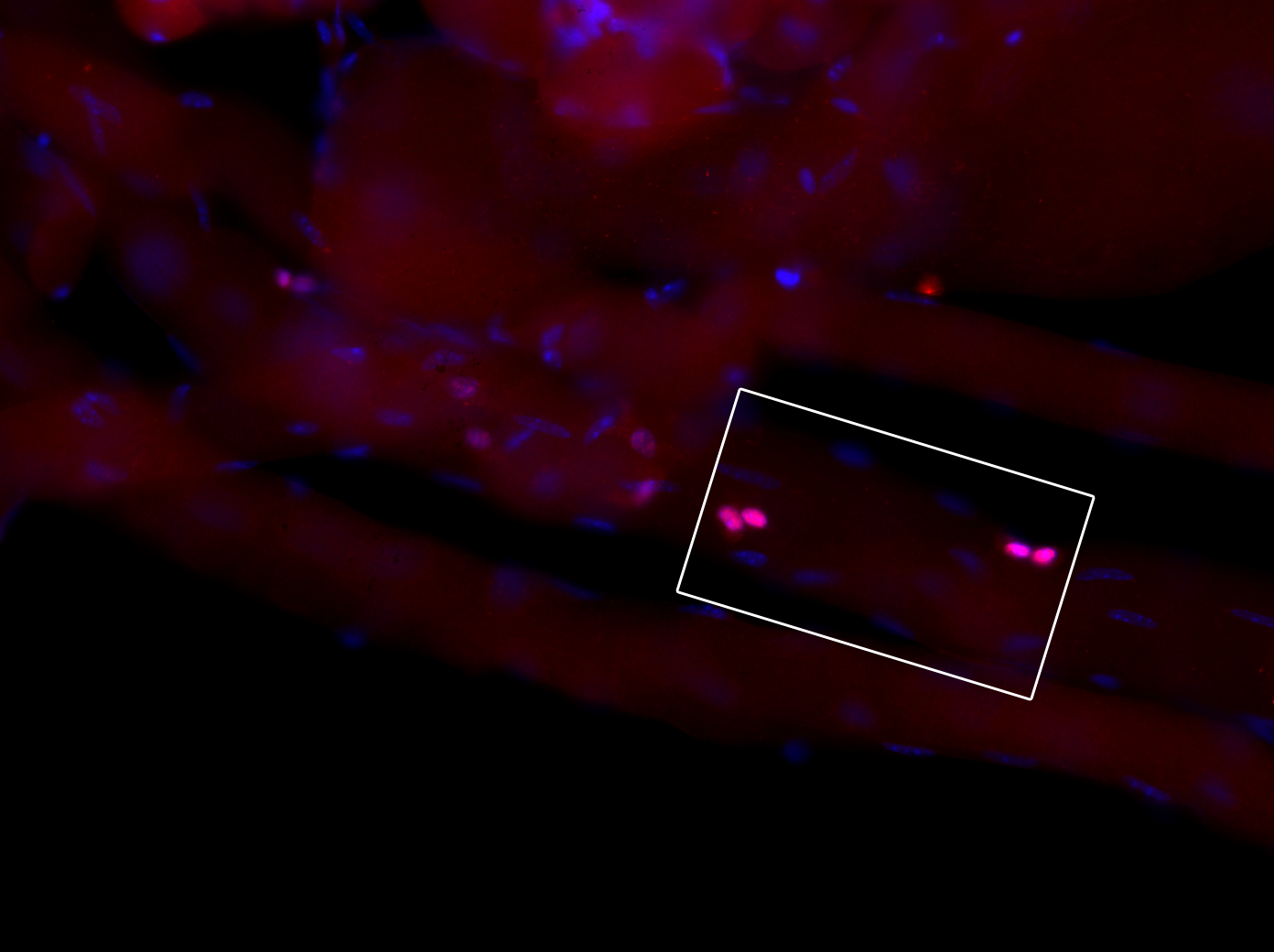

Supplement: Supplementary file 8 — Source data Fig. 6 [file 44318_2025_397_MOESM8_ESM.zip › Figure 6/Figure 6E/IF_Pax7_Fiber 60h_KO+Saline.tif]

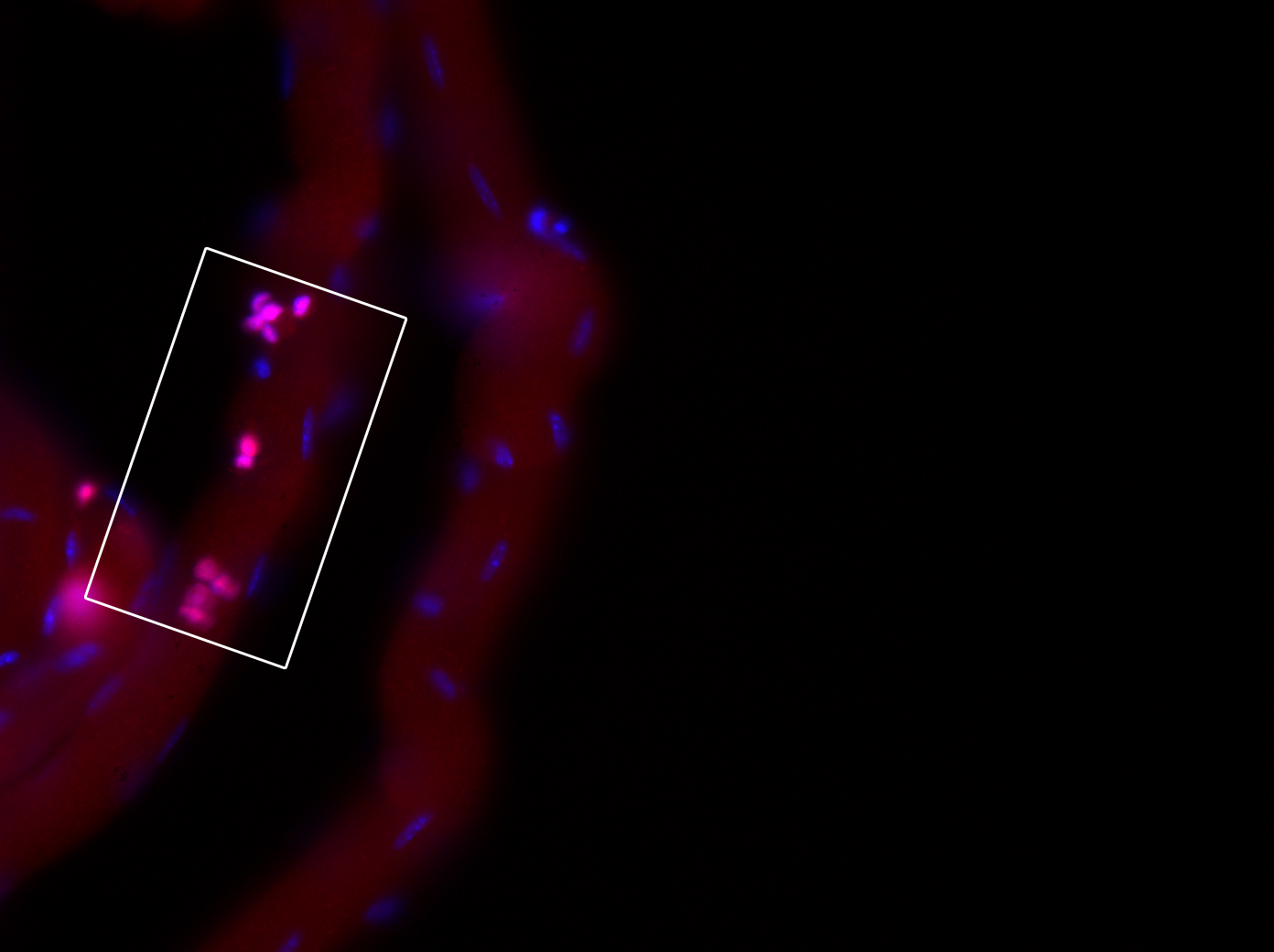

Supplement: Supplementary file 8 — Source data Fig. 6 [file 44318_2025_397_MOESM8_ESM.zip › Figure 6/Figure 6E/IF_Pax7_Fiber 60h_WT+Acetate.tif]

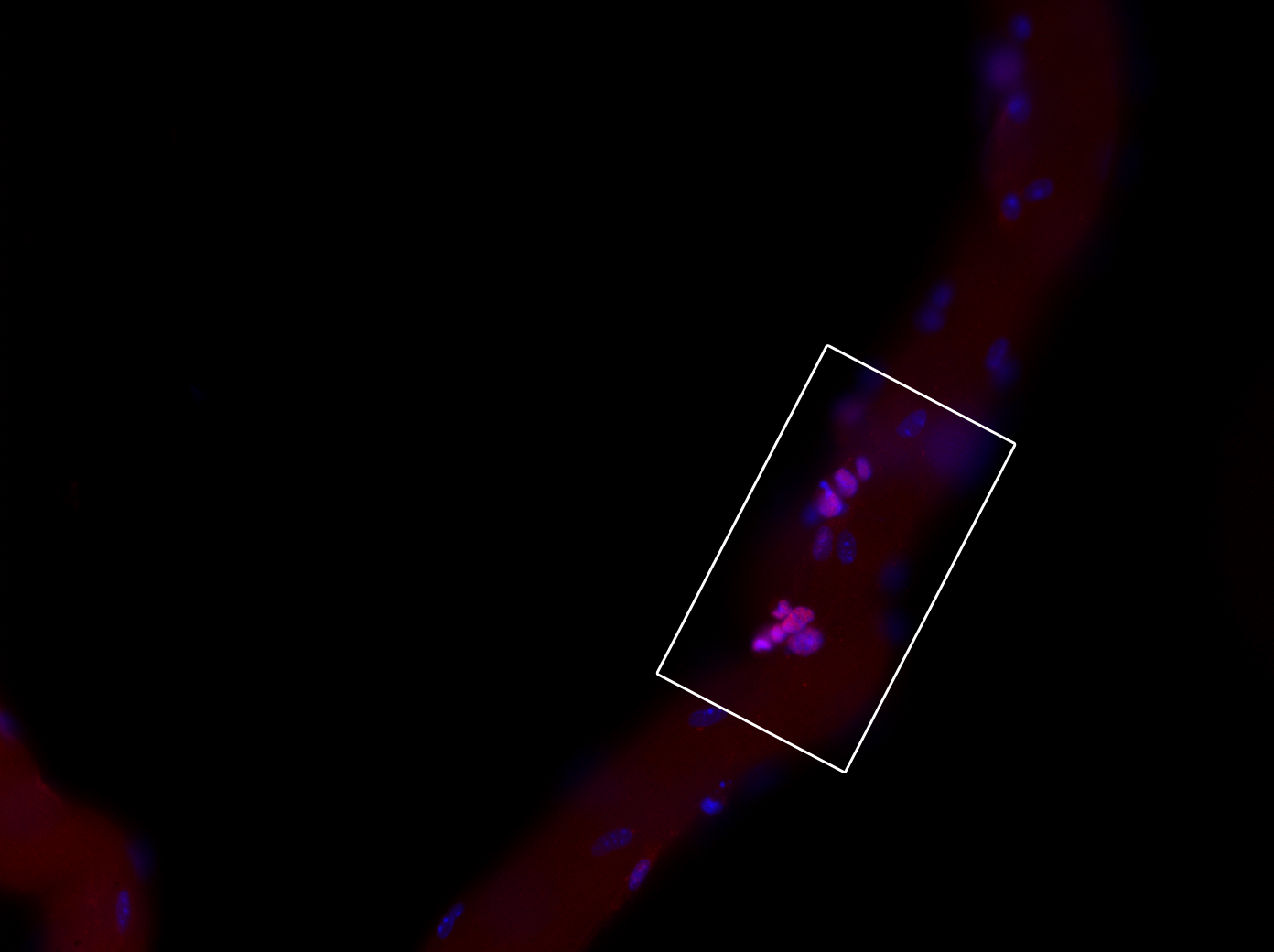

Supplement: Supplementary file 8 — Source data Fig. 6 [file 44318_2025_397_MOESM8_ESM.zip › Figure 6/Figure 6E/IF_Pax7_Fiber 60h_WT+Saline.tif]

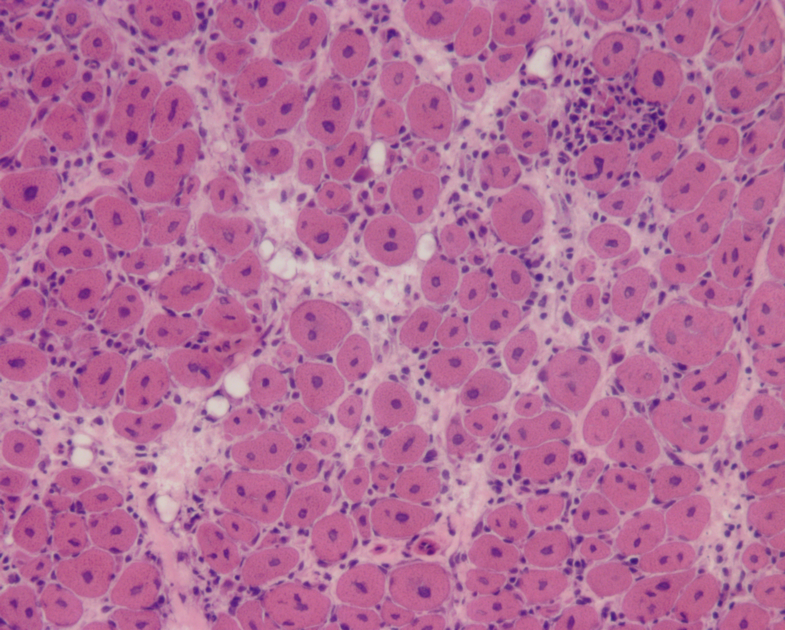

Supplement: Supplementary file 9 — Source data Fig. 7 [file 44318_2025_397_MOESM9_ESM.zip › Figure 7/Figure 7B/HE Staining_D5.5_KO+Acetate_TA.tif]

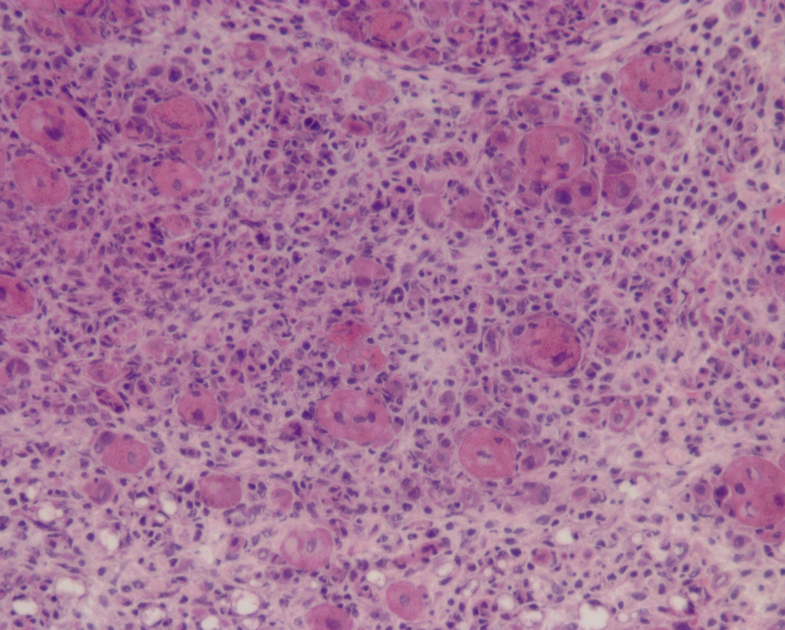

Supplement: Supplementary file 9 — Source data Fig. 7 [file 44318_2025_397_MOESM9_ESM.zip › Figure 7/Figure 7B/HE Staining_D5.5_KO+Saline_TA.tif]

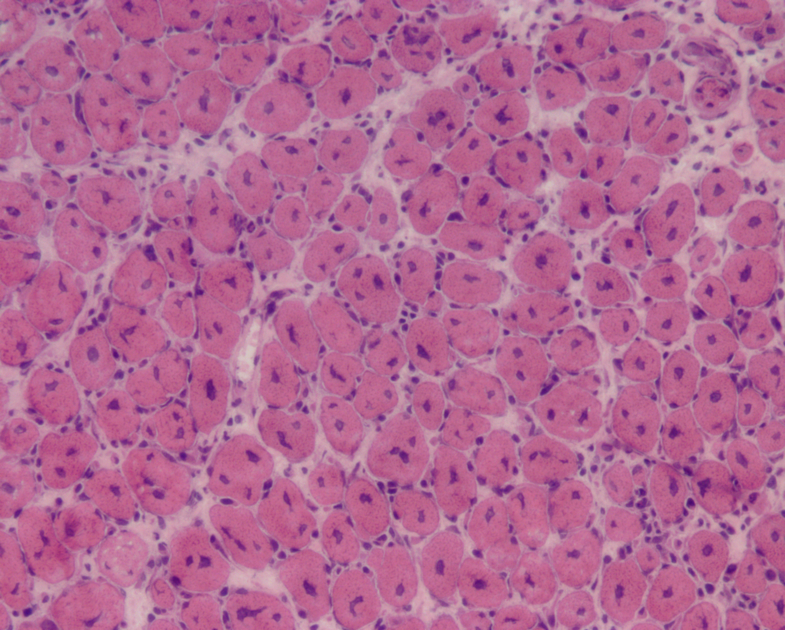

Supplement: Supplementary file 9 — Source data Fig. 7 [file 44318_2025_397_MOESM9_ESM.zip › Figure 7/Figure 7B/HE Staining_D5.5_WT+Acetate_TA.tif]

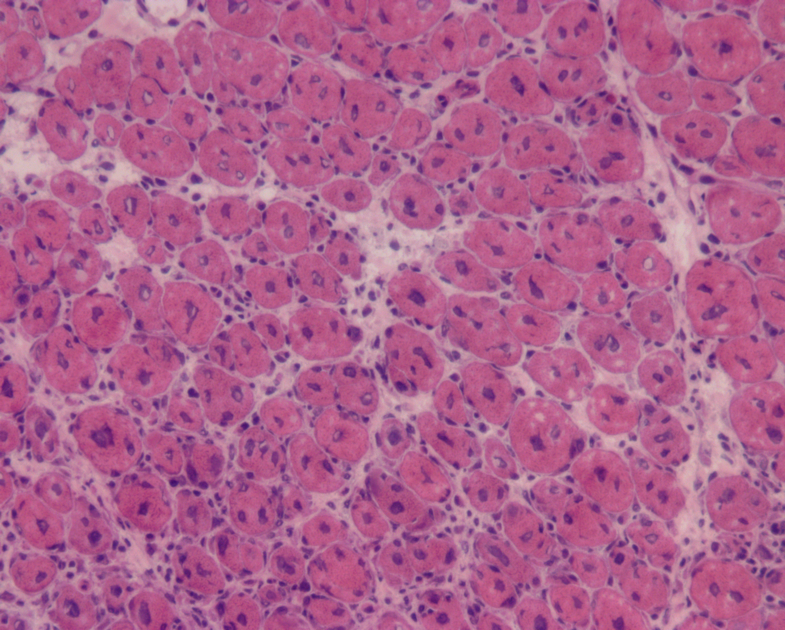

Supplement: Supplementary file 9 — Source data Fig. 7 [file 44318_2025_397_MOESM9_ESM.zip › Figure 7/Figure 7B/HE Staining_D5.5_WT+Saline_TA.tif]

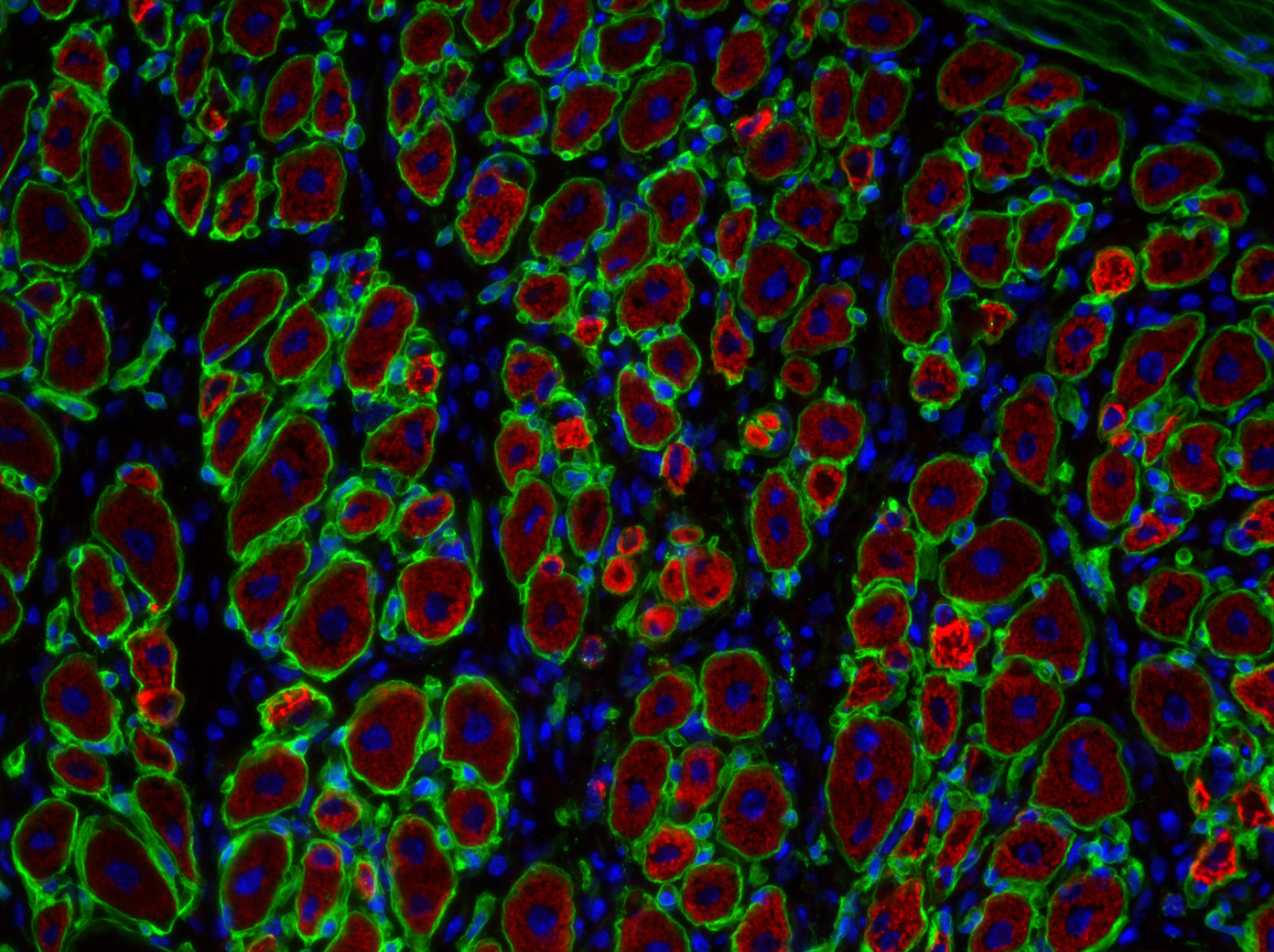

Supplement: Supplementary file 9 — Source data Fig. 7 [file 44318_2025_397_MOESM9_ESM.zip › Figure 7/Figure 7C/IF_eMyHC+Laminin_D5.5_KO+Acetate_TA.tif]

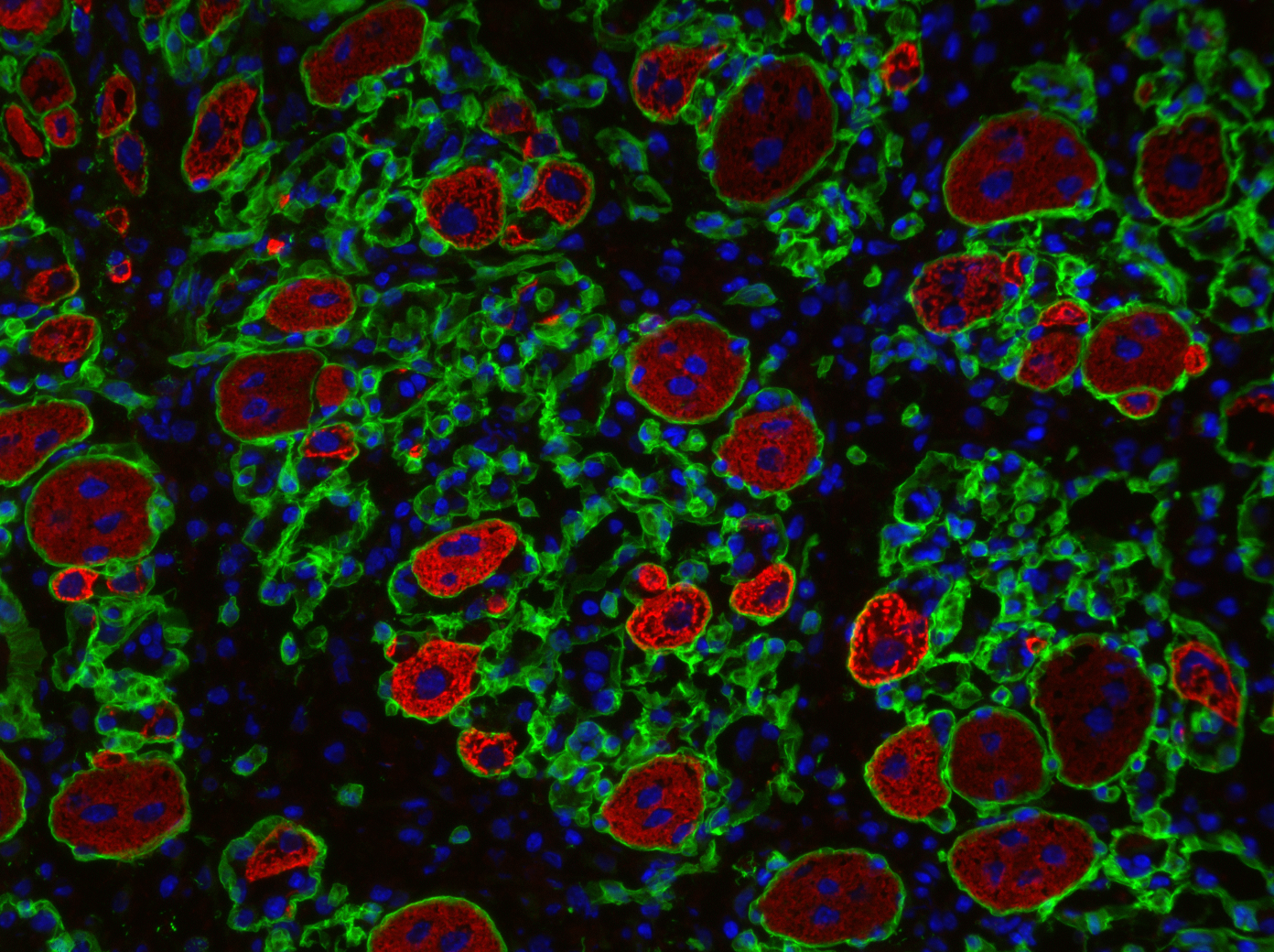

Supplement: Supplementary file 9 — Source data Fig. 7 [file 44318_2025_397_MOESM9_ESM.zip › Figure 7/Figure 7C/IF_eMyHC+Laminin_D5.5_KO+Saline_TA.tif]

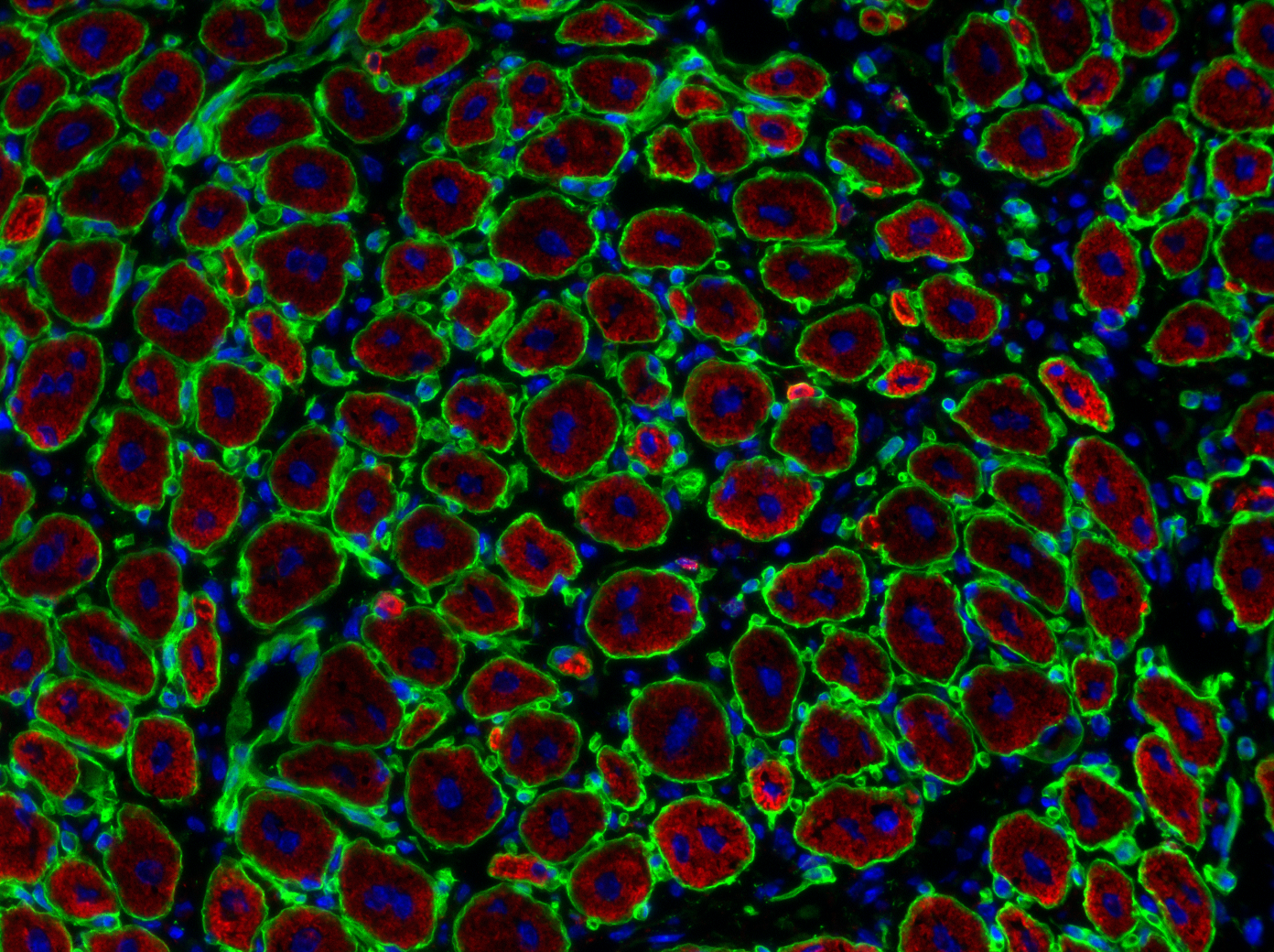

Supplement: Supplementary file 9 — Source data Fig. 7 [file 44318_2025_397_MOESM9_ESM.zip › Figure 7/Figure 7C/IF_eMyHC+Laminin_D5.5_WT+Acetate_TA.tif]

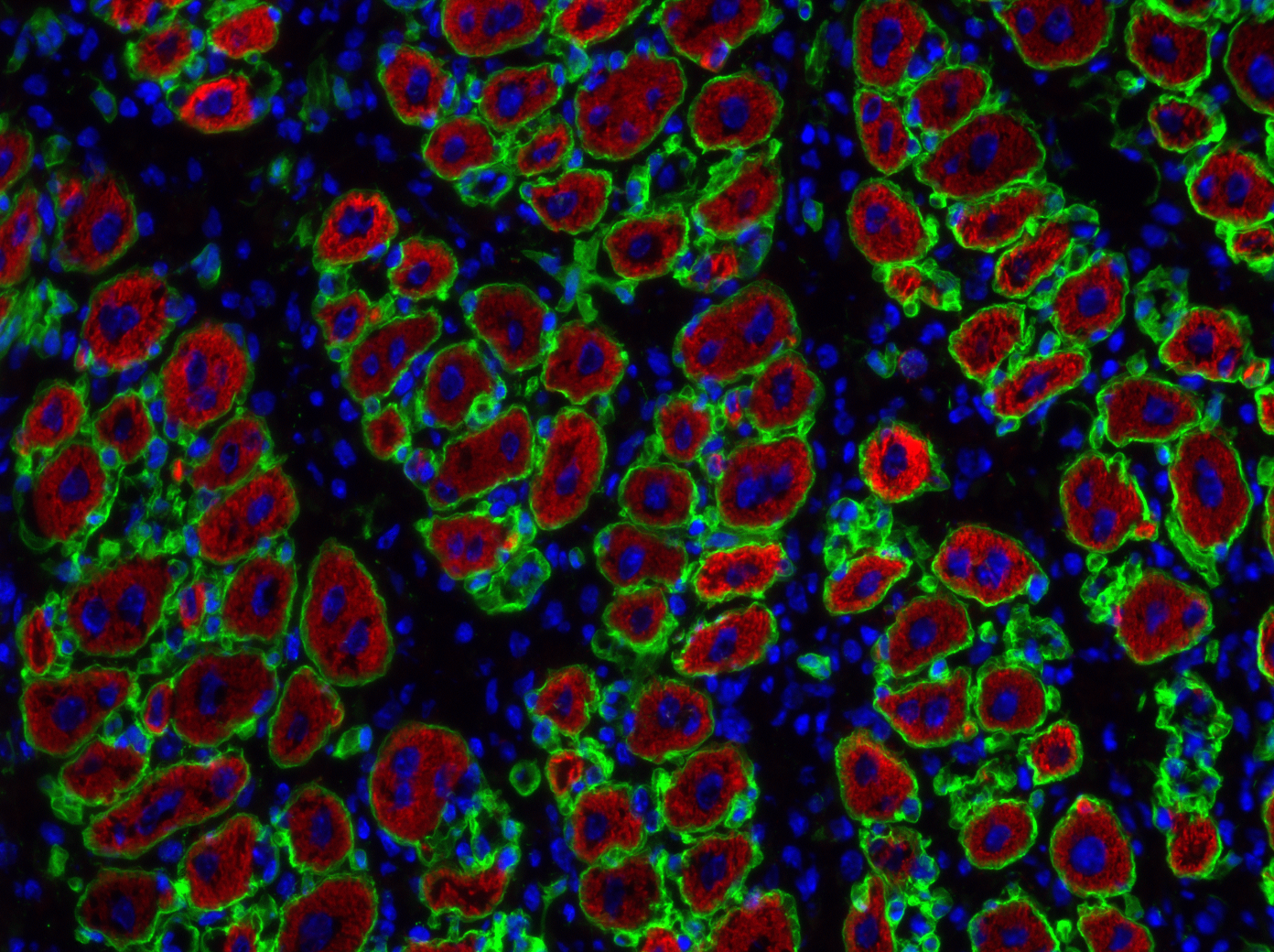

Supplement: Supplementary file 9 — Source data Fig. 7 [file 44318_2025_397_MOESM9_ESM.zip › Figure 7/Figure 7C/IF_eMyHC+Laminin_D5.5_WT+Saline_TA.tif]

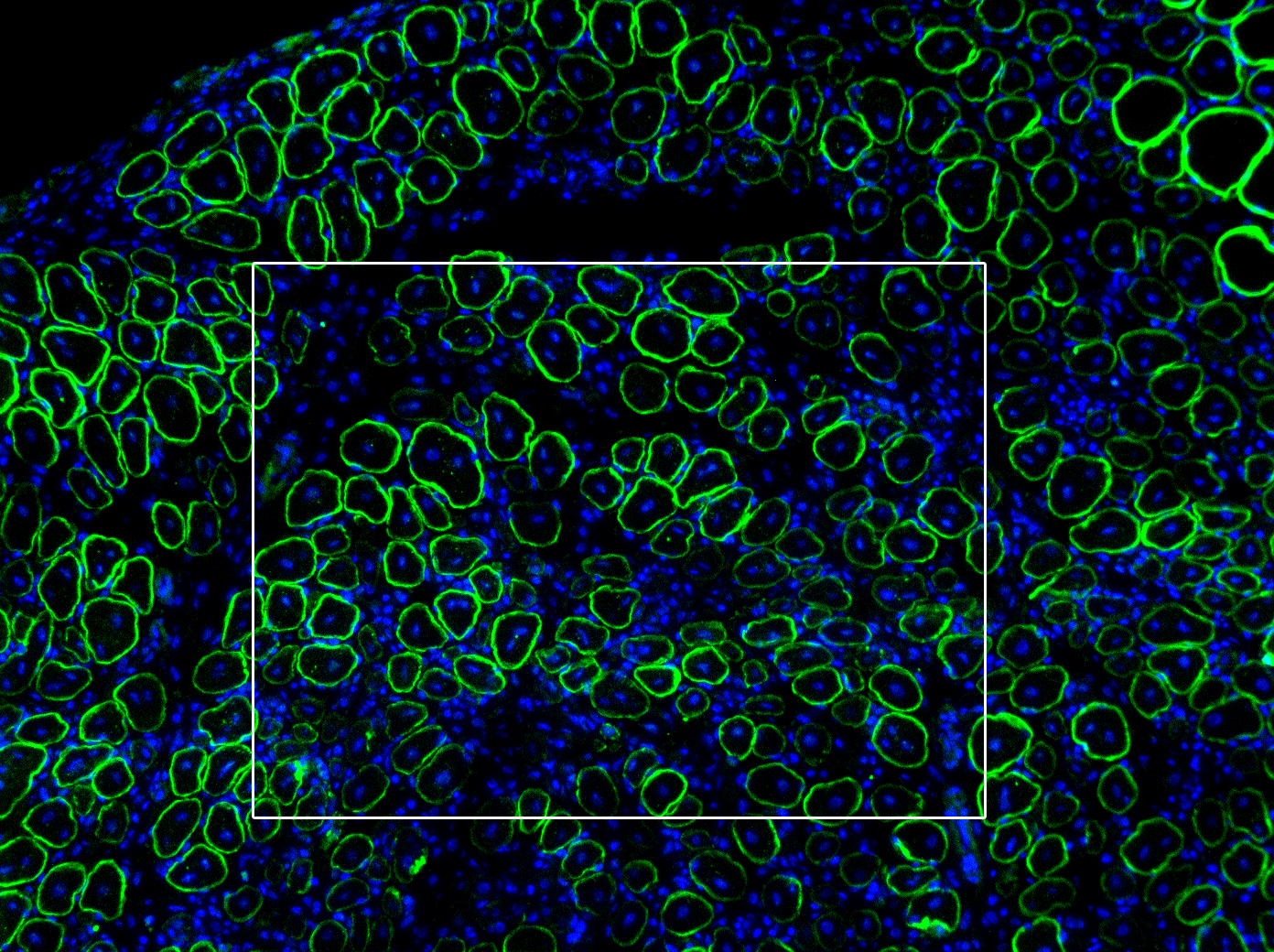

Supplement: Supplementary file 9 — Source data Fig. 7 [file 44318_2025_397_MOESM9_ESM.zip › Figure 7/Figure 7F/IF_Dystrophin_D5.5_KO+Acetate_TA.tif]

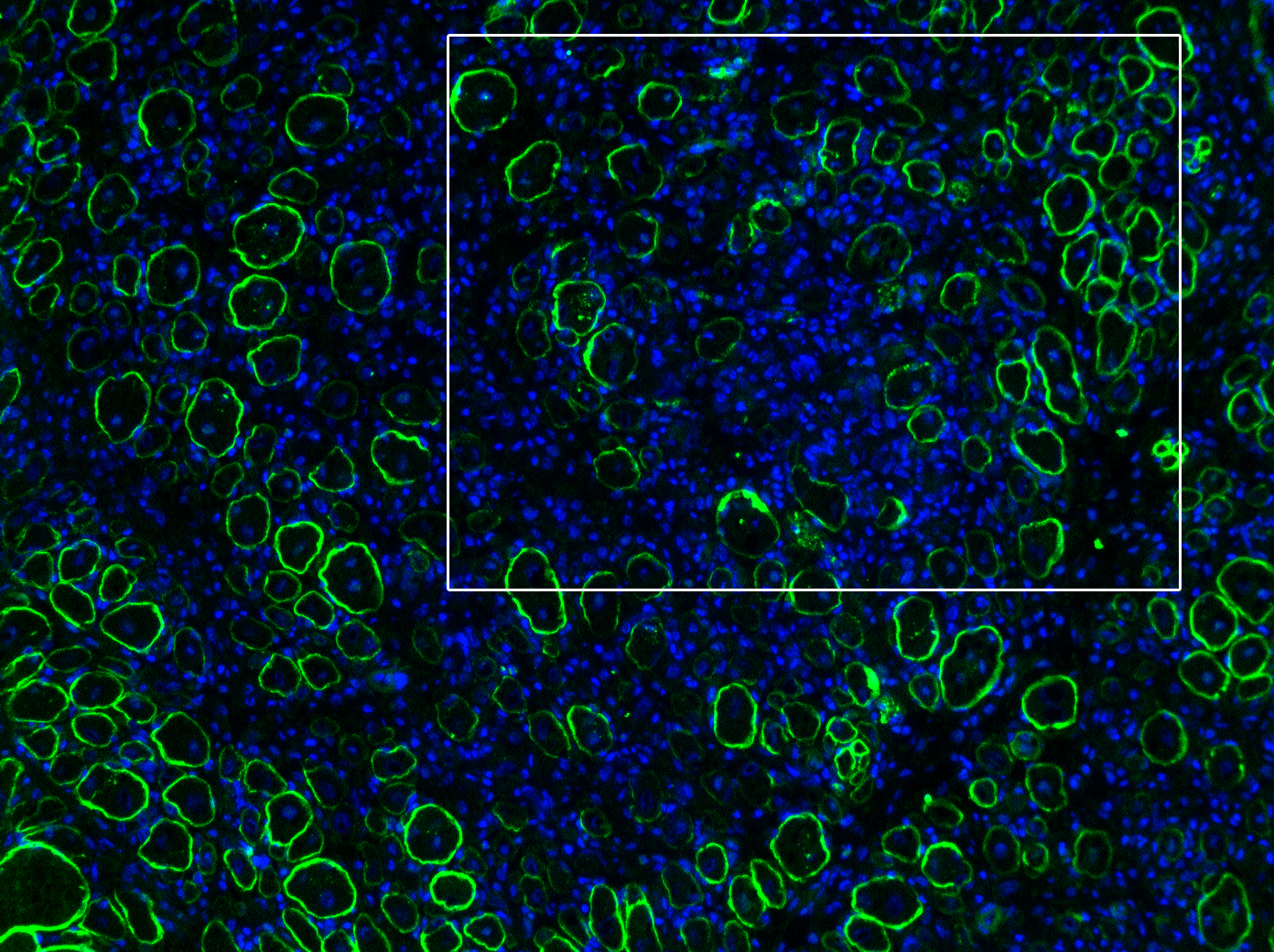

Supplement: Supplementary file 9 — Source data Fig. 7 [file 44318_2025_397_MOESM9_ESM.zip › Figure 7/Figure 7F/IF_Dystrophin_D5.5_KO+Saline_TA.tif]

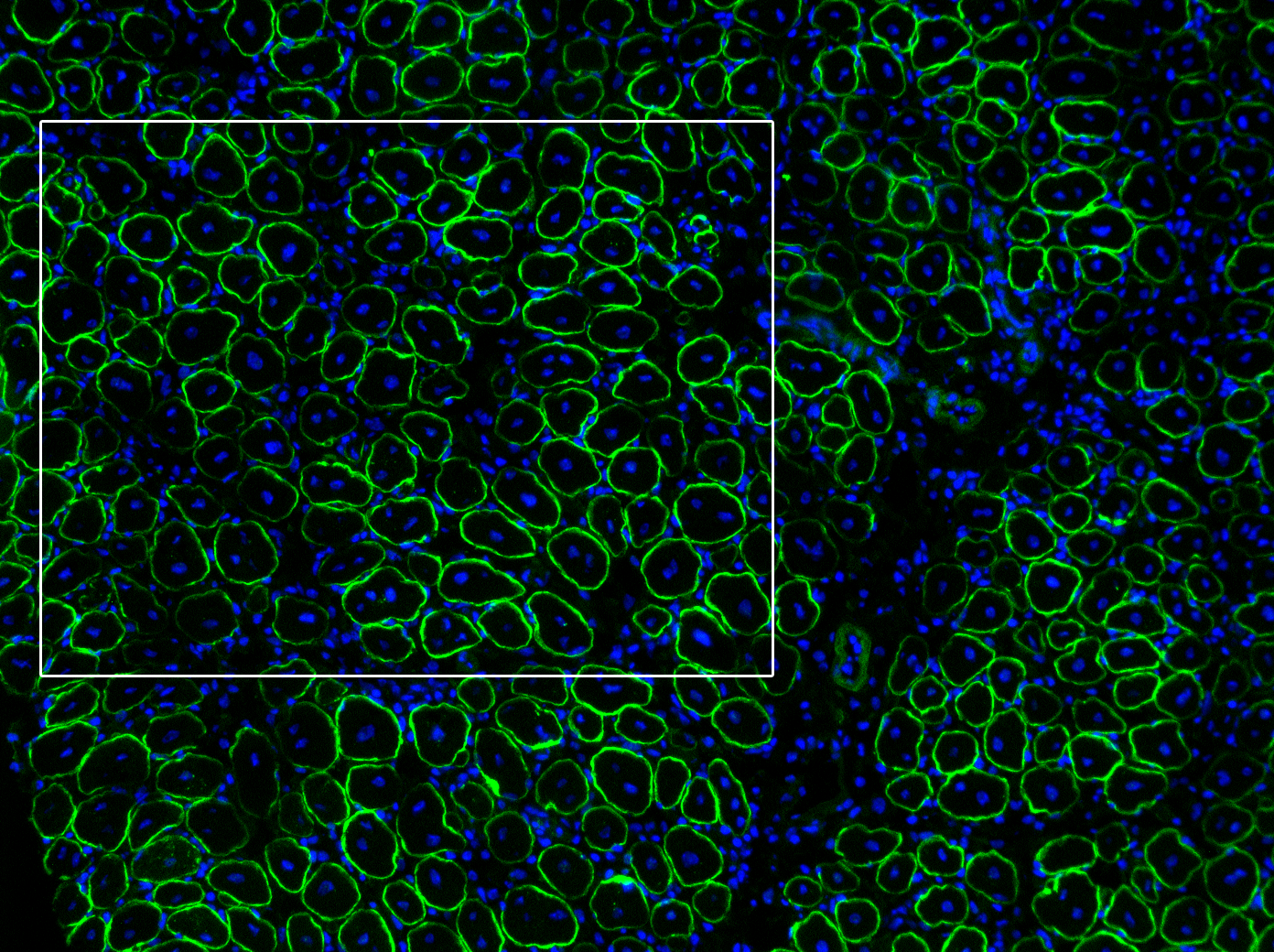

Supplement: Supplementary file 9 — Source data Fig. 7 [file 44318_2025_397_MOESM9_ESM.zip › Figure 7/Figure 7F/IF_Dystrophin_D5.5_WT+Acetate_TA.tif]

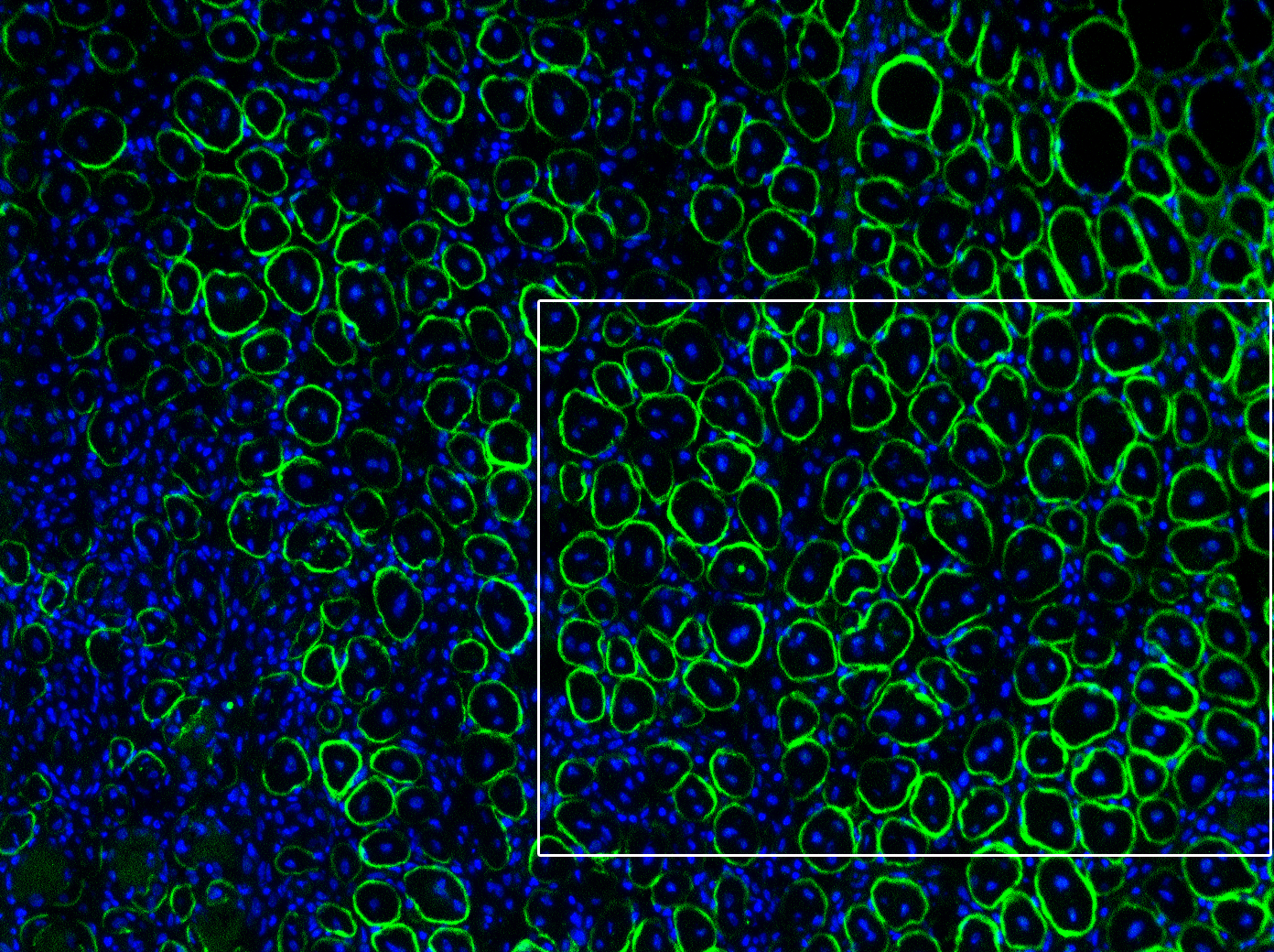

Supplement: Supplementary file 9 — Source data Fig. 7 [file 44318_2025_397_MOESM9_ESM.zip › Figure 7/Figure 7F/IF_Dystrophin_D5.5_WT+Saline_TA.tif]

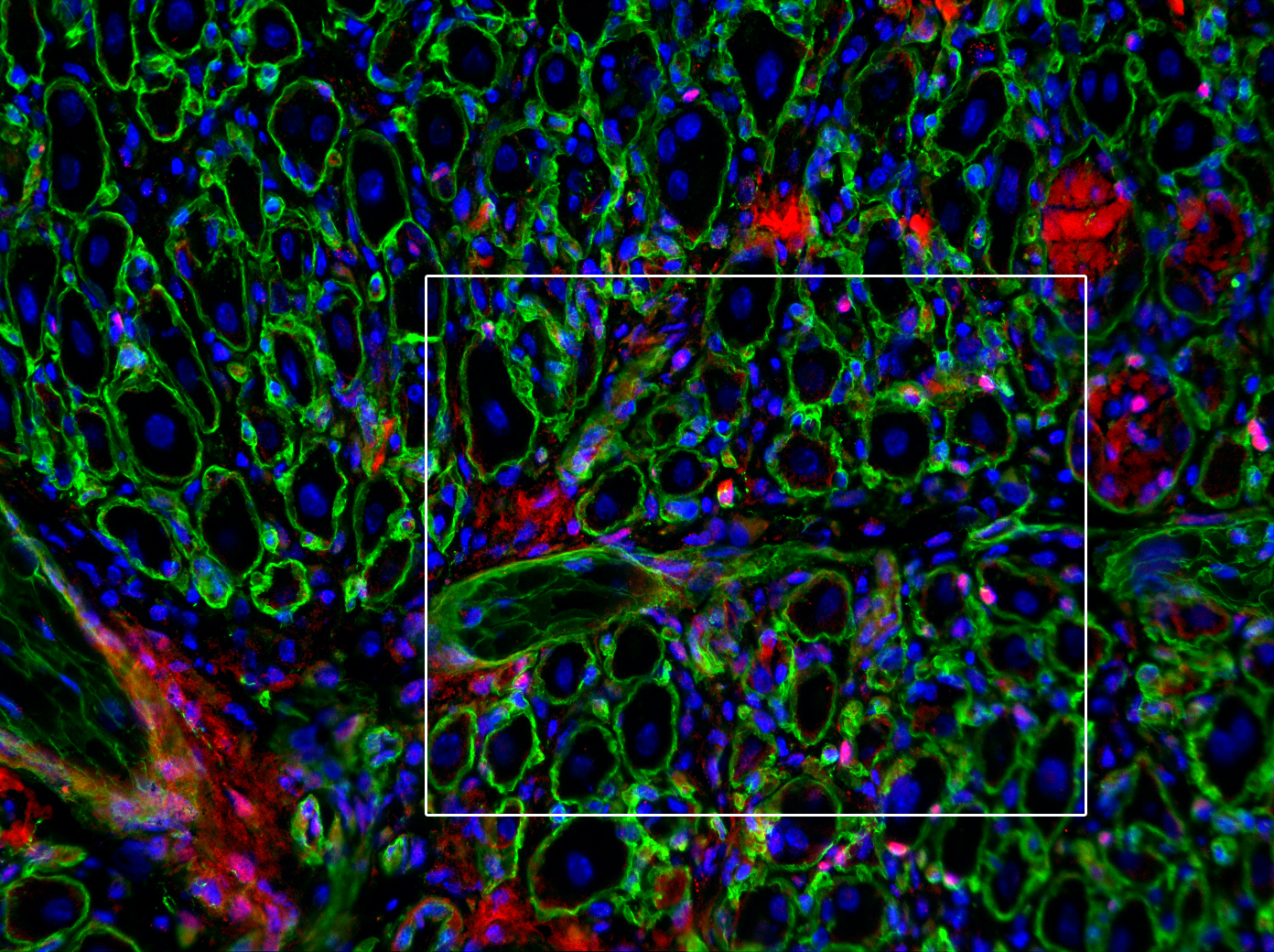

Supplement: Supplementary file 9 — Source data Fig. 7 [file 44318_2025_397_MOESM9_ESM.zip › Figure 7/Figure 7H/IF_Pax7+Laminin_D5.5_KO+Acetate_TA.tif]

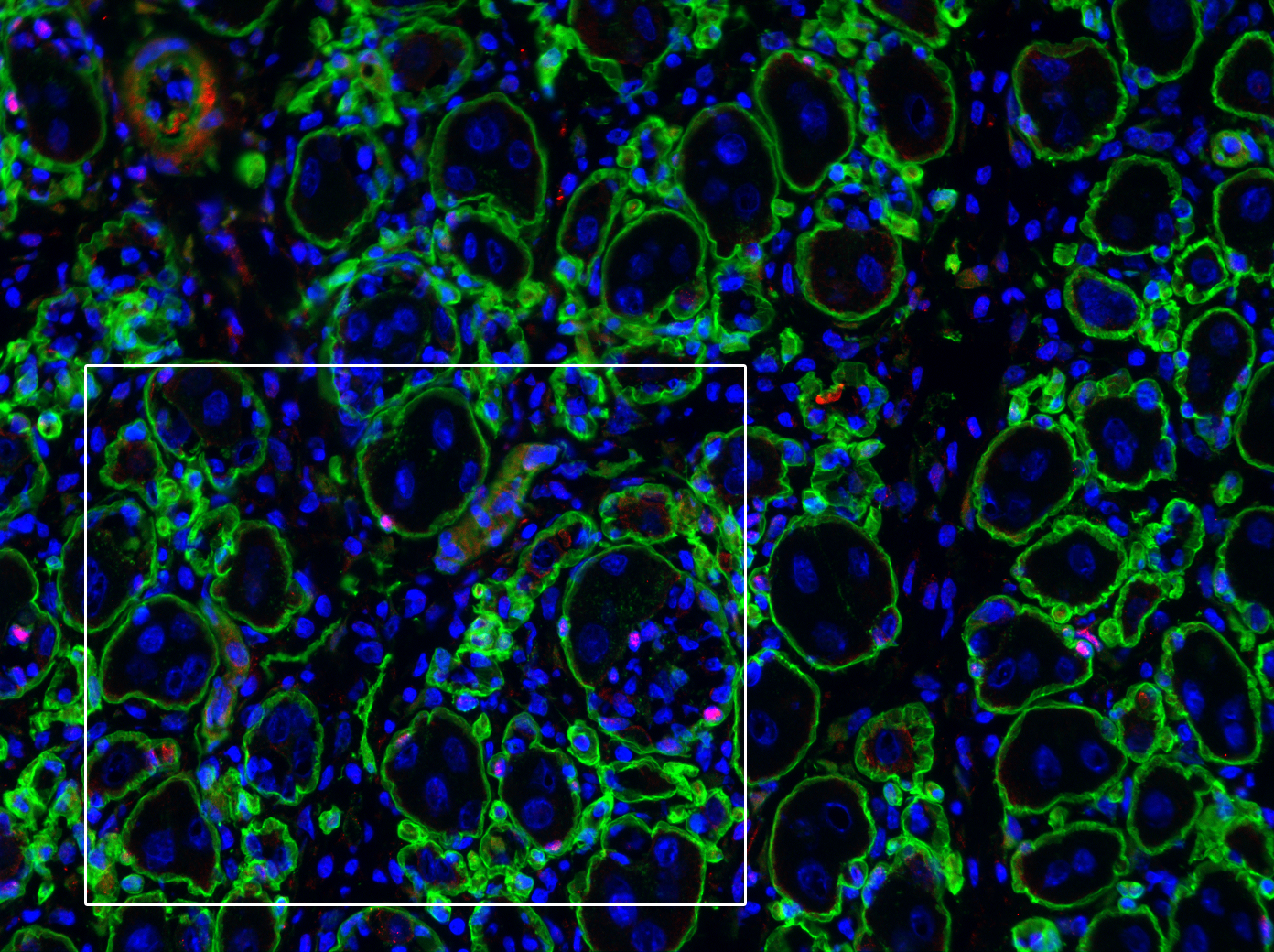

Supplement: Supplementary file 9 — Source data Fig. 7 [file 44318_2025_397_MOESM9_ESM.zip › Figure 7/Figure 7H/IF_Pax7+Laminin_D5.5_KO+Saline_TA.tif]

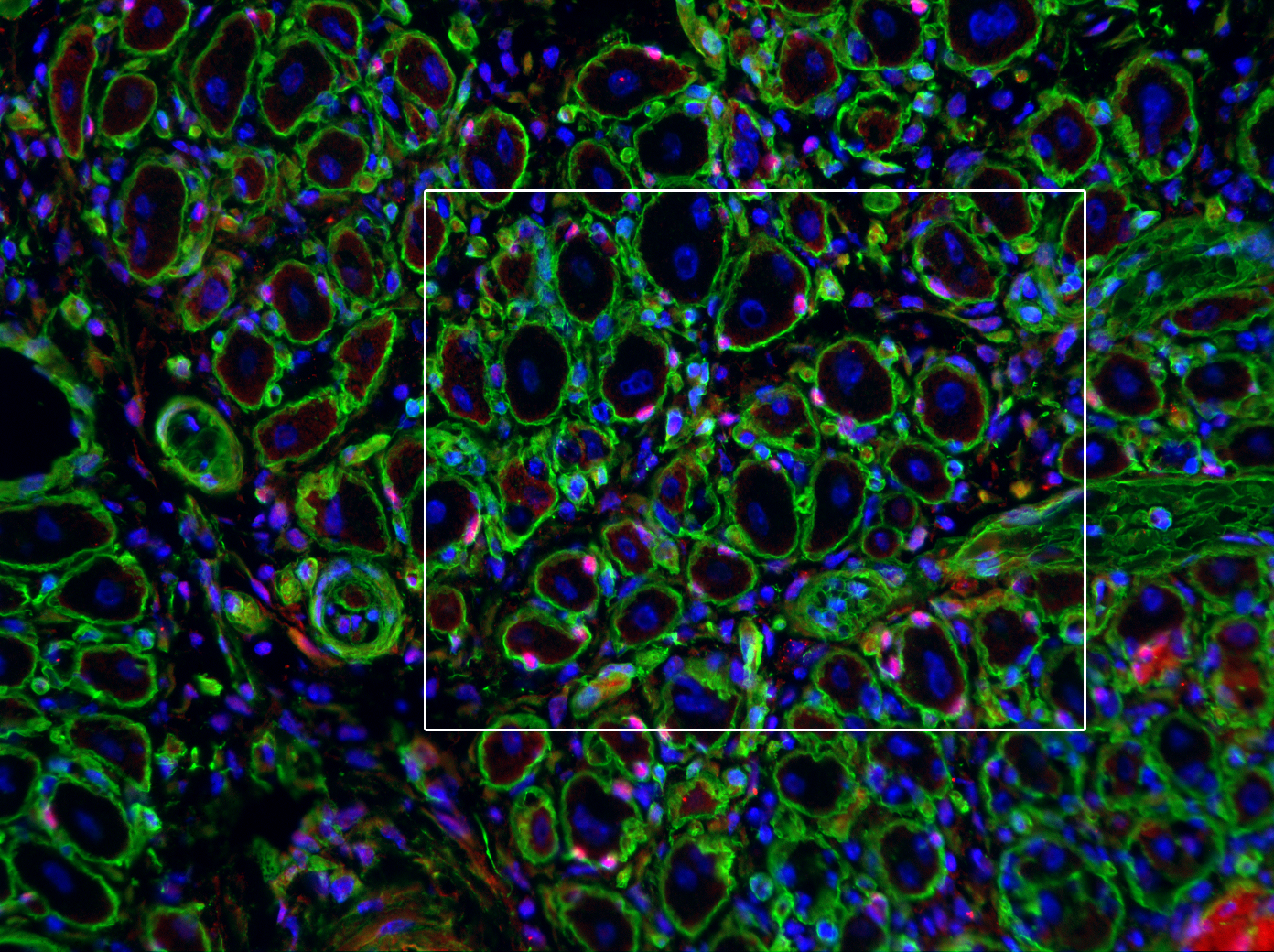

Supplement: Supplementary file 9 — Source data Fig. 7 [file 44318_2025_397_MOESM9_ESM.zip › Figure 7/Figure 7H/IF_Pax7+Laminin_D5.5_WT+Acetate_TA.tif]

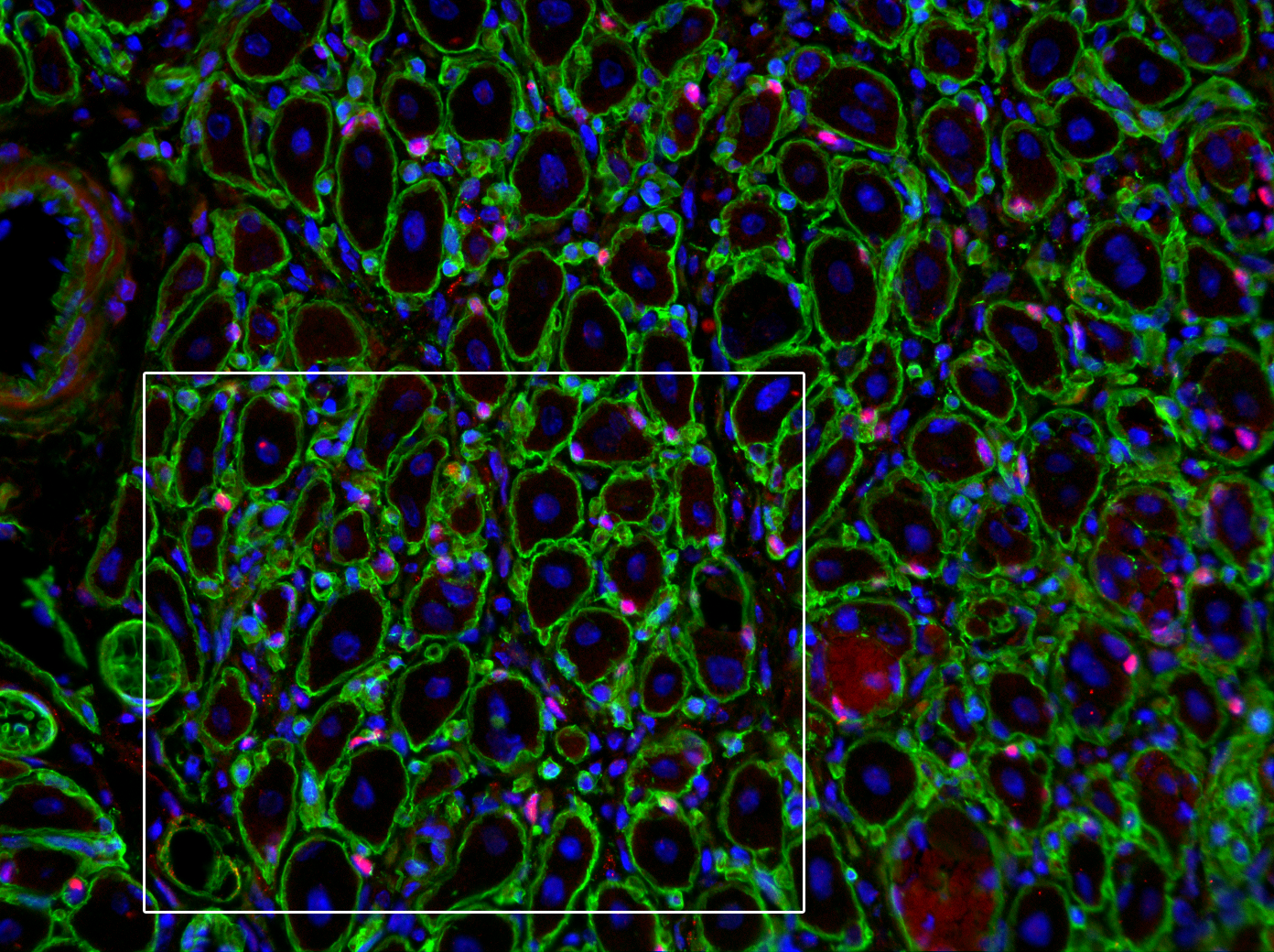

Supplement: Supplementary file 9 — Source data Fig. 7 [file 44318_2025_397_MOESM9_ESM.zip › Figure 7/Figure 7H/IF_Pax7+Laminin_D5.5_WT+Saline_TA.tif]
