# Supplementary material for: Mitochondrial fatty acid oxidation regulates adult muscle stem cell function through modulating metabolic flux and protein acetylation
Source: EMBO J. 2025 Mar 10;44(9):2566–95. doi: 10.1038/s44318-025-00397-1 (PMC12048568; doi:10.1038/s44318-025-00397-1)
Supplement: Supplementary file 10 — Expanded View Figures [file 44318_2025_397_MOESM10_ESM.pdf]

Expanded View Figures

Figure EV1. Meta-cluster analysis with top markers of each SC state.

QSC quiescent SCs, SSC self-renewed SCs, ASC activated SCs, PSC proliferating SCs, CSC committed SCs, DSC differentiated SCs.

© The Author(s)

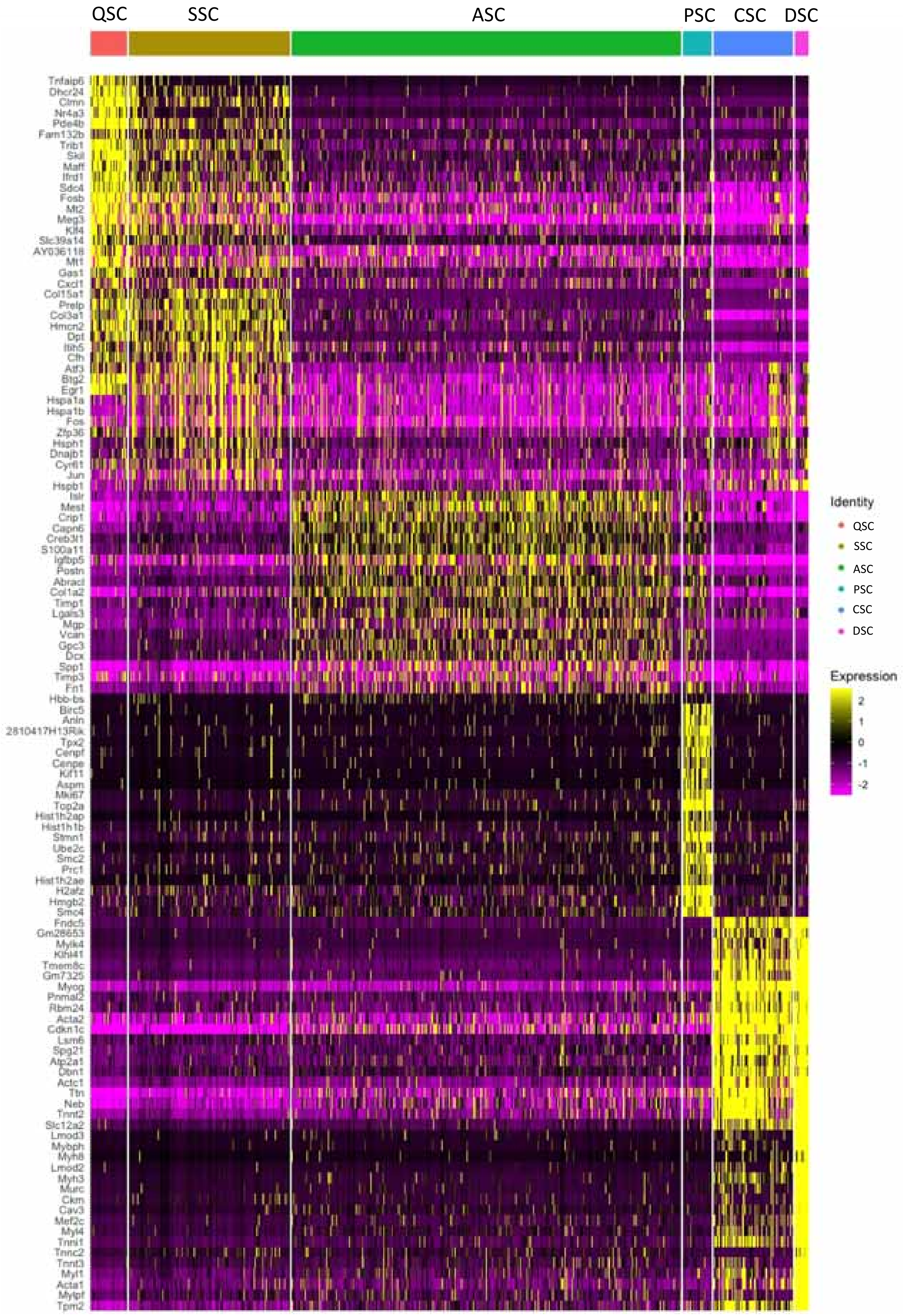
QSC
SSC
ASC
PSC
CSC
DSC
Tnfaip6
Dhcr24
Clmn
Nr4a3
Pde4b
Fam132b
Trib1
Skil
Maff
Ifrd1
Sdc4
Fosb
Mt2
Meg3
Klf4
Slc39a14
AY036118
Mt1
Gas1
Cxcl1
Col15a1
Prelp
Col3a1
Hmcn2
Dpt
Itih5
Cfh
Atf3
Btg2
Egr1
Hspa1a
Hspa1b
Fos
Zfp36
Hsph1
Dnajb1
Cyr61
Jun
Hspb1
Islr
Mest
Crip1
Capn6
Creb3l1
S100a11
Igfbp5
Postn
Abracl
Col1a2
Timp1
Lgals3
Mgp
Vcan
Gpc3
Dcx
Spp1
Timp3
Fn1
Hbb-bs
Birc5
Anln
2810417H13Rik
Tpx2
Cenpf
Cenpe
Kif11
Aspm
Mki67
Top2a
Hist1h2ap
Hist1h1b
Stmn1
Ube2c
Smc2
Prc1
Hist1h2ae
H2afz
Hmgb2
Smc4
Fndc5
Gm28653
Mylk4
Klhl41
Tmem8c
Gm7325
Myog
Pnmal2
Rbm24
Acta2
Cdkn1c
Lsm6
Spg21
Atp2a1
Dbn1
Actc1
Ttn
Neb
Tnnt2
Slc12a2
Lmod3
Mybph
Myh8
Lmod2
Myh3
Murc
Ckm
Cav3
Mef2c
Myl4
Tnni1
Tnnc2
Tnnt3
Myl1
Acta1
Mylpf
Tpm2
Identity
QSC
SSC
ASC
PSC
CSC
DSC
Expression
2
1
0
-1
-2

 © The Author(s)

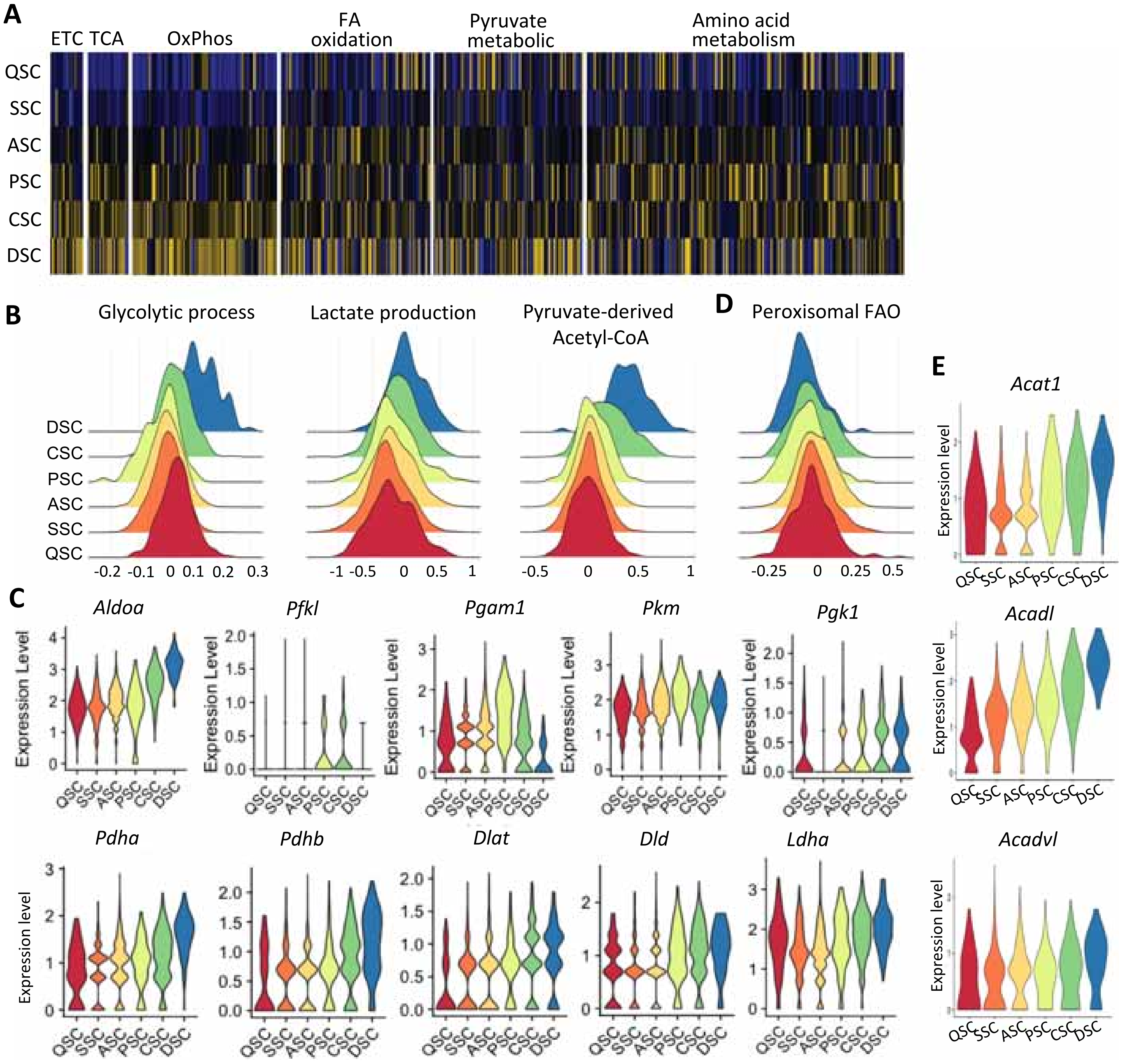

Figure EV2. Dynamics of metabolic processes during fate transitions of satellite cell.

(**A**) Heatmap showing enrichment of genes in various annotated Gene Ontology terms. ETC, GO:0022900; TCA cycle, GO:0006099; OxPhos, GO:0006119; FA oxidation, GO:00019395; pyruvate metabolism, GO: 0006090; amino acid metabolism, GO:0006520. (**B**) Density plot visualizing the enrichment of genes involving in glycolytic processes over different states. Glycolytic process, GO:0006096; lactate catabolism, GO: 0019244; acetyl-CoA biosynthetic process from pyruvate, GO: 0006086; (**C**) Violin plot showing the expression of key genes involved in glycolytic process (GO:0006096, top row) and acetyl-CoA biosynthetic process from pyruvate (GO: 0006086, bottom row). (**D**) Density plot visualizing the enrichment of genes involving in peroxisomal fatty acid oxidation (FAO) over different states. (**E**) Violin plot showing the expression of key genes involved in long-chain FAO among different states.

© The Author(s)

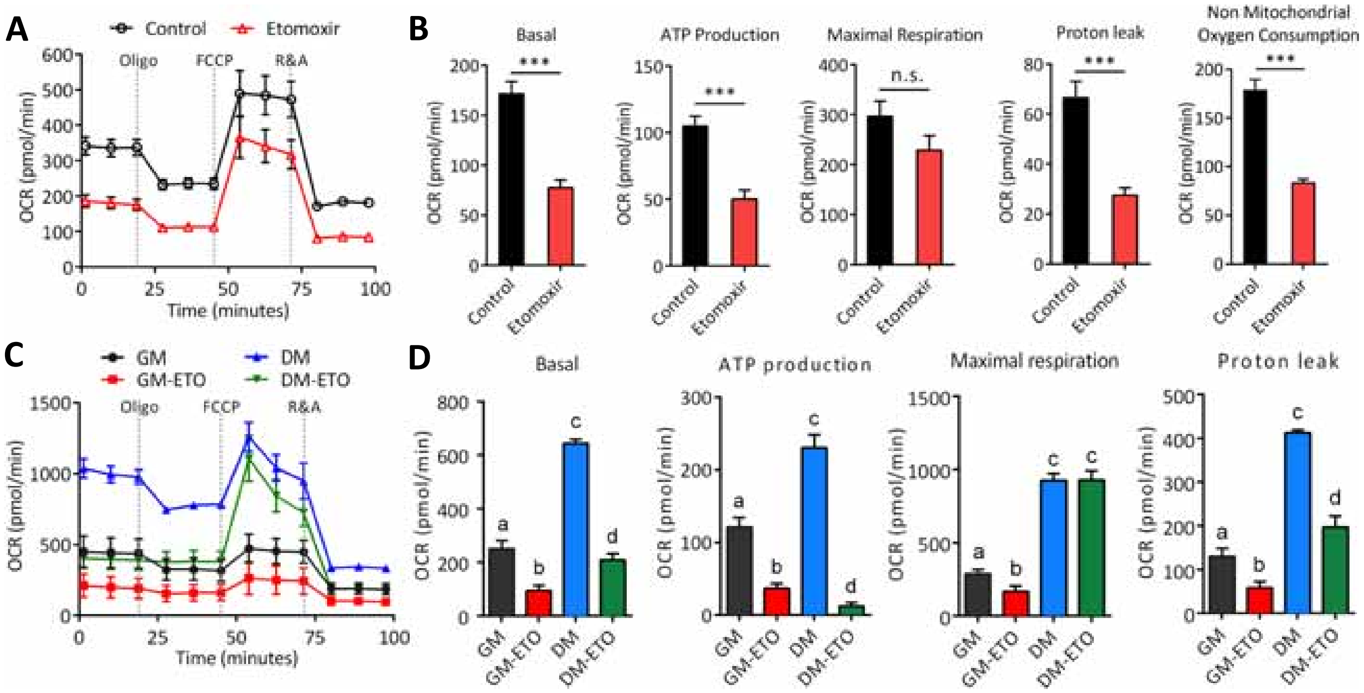

Figure EV3. Pharmacological inhibition of FAO disturbs mitochondrial respiration and inhibits proliferation and differentiation of myoblasts.

(**A**) Seahorse curves showing oxygen consumption rates (OCR) of primary myoblasts treated with vehicle control or 50 μM Etomoxir (a mitochondrial FAO inhibitor). $n = 3$ biological replicates, three technical replicates per run. (**B**) OCR associated with basal and maximal respiration, proton leak, ATP production and non-mitochondrial respiration, calculated based on data shown in (**A**). Error bars represent mean ± s.e.m. with $n = 3$ biological replicates (three technical replicates per run). ***$P < 0.001$; two tailed, unpaired Student's t test (Basal, $P = 7.03 \times 10^{-5}$; ATP production, $P = 9.97 \times 10^{-5}$; maximal respiration, $P = 0.1796$; proton leak, $P = 0.00014$; non-mitochondrial oxygen consumption, $P = 9.7 \times 10^{-5}$). n.s. no significance. (**C**) Seahorse curve showing OCR of undifferentiated myoblasts and differentiated myotubes treated with vehicle control or Etomoxir. (**D**) Quantification of the OCR regarding to basal respiration, proton leak, ATP production and spare respiratory capacity measured from seahorse assay in (**C**). Error bars represent mean ± s.e.m. with $n = 4$ replicates. One-way ANOVA with Tukey's post hoc comparison was used.

© The Author(s)

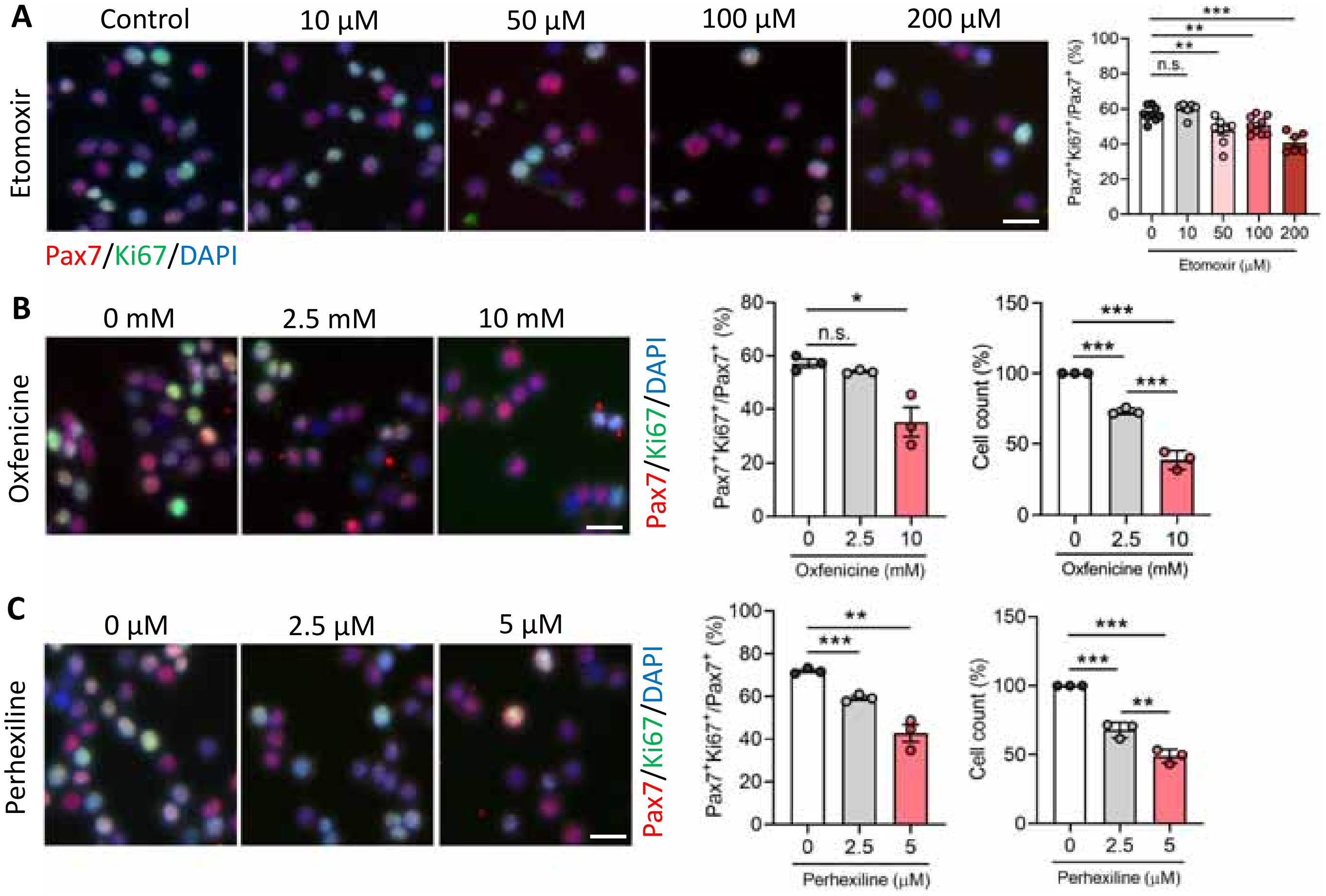

Figure EV4. Pharmacological inhibition of FAO inhibits the proliferation of myoblasts.

(**A**) Ki67 immunofluorescence (green) and quantification of percentages of Ki67+ primary myoblasts treated with vehicle control or Etomoxir. Error bars represent mean ± s.e.m. with $n = 6$–9 replicates. **$P < 0.01$, ***$P < 0.001$; two tailed, unpaired Student's t test (10 μM vs. control, $P = 0.2519$; 50 μM vs. control, $P = 0.0042$; 100 μM vs. control, $P = 0.0087$; 200 μM vs. control, $P = 2.42 \times 10^{-5}$). n.s. no significance. Scale bar, 50 μm. (**B**) Ki67 immunofluorescence (green) and quantification of percentages of Ki67+ primary myoblasts treated with vehicle control or Oxfenicine. Error bars represent mean ± s.e.m. with $n = 3$ replicates. *$P < 0.05$, ***$P < 0.001$; two tailed, unpaired Student's t test. For Ki67+/Pax7+ percentage, 2.5 mM vs. control, $P = 0.13$; 10 mM vs. control, $P = 0.0187$. For cell count (%), 2.5 mM vs. control, $P = 0.00016$; 10 mM vs. control, $P = 7.97 \times 10^{-7}$; 2.5 mM vs. 10 mM, $P = 0.00017$; n.s. no significance. Scale bar, 50 μm. (**C**) Ki67 immunofluorescence (green) and quantification of percentages of Ki67+ primary myoblasts treated with vehicle control or Perhexiline. Error bars represent mean ± s.e.m. with $n = 6$–9 replicates. **$P < 0.01$, ***$P < 0.001$; two tailed, unpaired Student's t-test. For Ki67+/Pax7+ percentage, 2.5 μM vs. control, $P = 0.00049$; 5 μM vs. control, $P = 0.00231$; For cell count (%), 2.5 μM vs. control, $P = 0.00011$; 5 μM vs. control, $P = 7.43 \times 10^{-5}$; 2.5 μM vs. 5 μM, $P = 0.0037$. Scale bar, 50 μm.

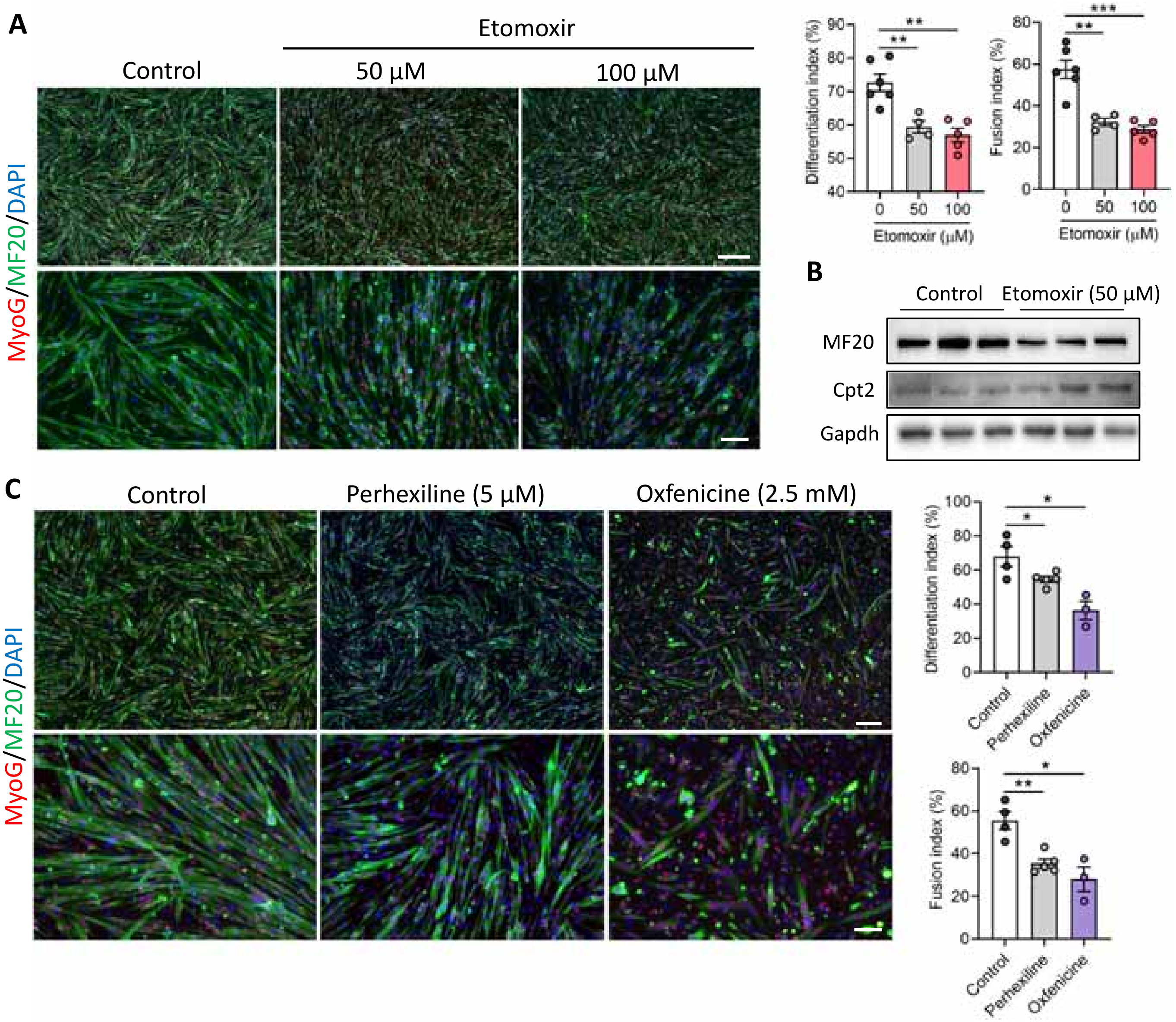

Figure EV5. Pharmacological inhibition of FAO inhibits the differentiation of primary myoblasts.

(**A**) Immunofluorescence of MyoG (red) and MF20 (green) and quantification of differentiation and fusion index in primary myoblasts treated with vehicle control or Etomoxir. Error bars represent mean ± s.e.m. with $n = 4$–6 replicates. $^{**}P < 0.01$, $^{***}P < 0.001$; two tailed, unpaired Student's *t*-test. For differentiation index, 50 µM vs. control, $P = 0.0057$; 100 µM vs. control, $P = 0.0013$. For fusion index, 50 µM vs. control, $P = 0.0021$; 100 µM vs. control, $P = 0.00032$. Scale bar, 500 µm (top); 100 µm (bottom). (**B**) Immunoblots showing relative levels of MF20 and Cpt2 in primary myoblasts treated with vehicle control or Etomoxir (50 µM). (**C**) Immunofluorescence of MyoG (red) and MF20 (green) and quantification of differentiation and fusion index in primary myoblasts treated with vehicle control or Perhexiline (5 µM) and Oxfenicine (2.5 mM). Error bars represent mean ± s.e.m. with $n = 3$-6 replicates. $^{*}P < 0.05$, $^{**}P < 0.01$; two tailed, unpaired Student's *t* test. For differentiation index, Perhexiline vs. control, $P = 0.0472$; Oxfenicine vs. control, $P = 0.012$. For fusion index, Perhexiline vs. control, $P = 0.0025$; Oxfenicine vs. control, $P = 0.01052$. Scale bar, 200 µm (top); 100 µm (bottom).

 © The Author(s)

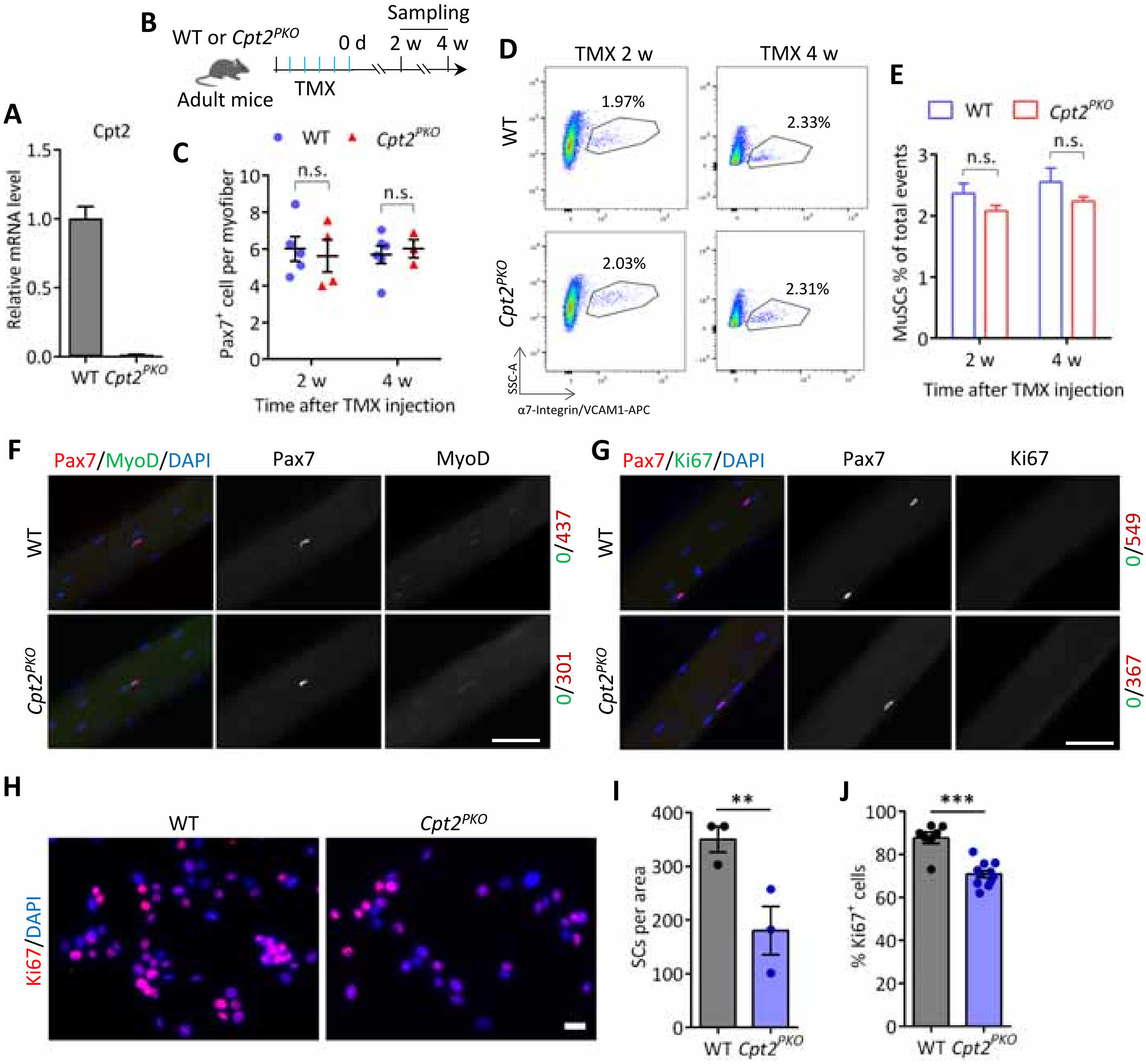

Figure EV6. Loss of *Cpt2* does not affect the quiescent maintenance of satellite cells (SCs) but inhibits their proliferative rate upon activation.

(**A**) qPCR analysis to validate the KO efficiency of CPT2 gene in FACS-sorted SCs at 3.5 dpi. (**B**) Schematic illustration of experimental design. TMX injection was used to induce *Cpt2* knockout in the *Cpt2*^{PKO} mice. (**C**) Numbers of Pax7$^+$ SCs per EDL myofiber at 2 weeks (WT $n = 5$, *Cpt2*^{PKO} $n = 4$) and 4 weeks (WT $n = 6$, *Cpt2*^{PKO} $n = 3$) after the last TMX injection. Error bars represent mean ± s.e.m. with $n = 3$–6 mice. n.s. no significant difference; two tailed, unpaired Student's *t* test (for 2 weeks, WT vs. *Cpt2*^{PKO}, $P = 0.737$; for 4 weeks, WT vs. *Cpt2*^{PKO}, $P = 0.763$). (**D**) Representative flow cytometry results showing gating of SCs (Sca1$^-$CD31$^-$CD45$^-$ITGA7$^+$VCAM$^+$) in mononuclear cells freshly isolated from hindlimb muscles. (**E**) Percentage of SCs in WT and *Cpt2*^{PKO} mice quantified based on flow cytometry analysis as shown in (**D**). Error bars represent mean ± s.e.m. with $n = 2$–4 mice. n.s. no significant difference; two tailed, unpaired Student's *t* test (for 2 weeks, WT vs. *Cpt2*^{PKO}, $P = 0.179$; for 4 weeks, WT vs. *Cpt2*^{PKO}, $P = 0.31$). (**F, G**) Immunostaining of MyoD (**F**) and Ki67 (**G**), along with Pax7, in EDL myofibers freshly isolated from WT and *Cpt2*^{PKO} mice 4 weeks after TMX induction. Numbers were the MyoD or Ki67 positive cells among total cells counted. (**H**) Immunostaining of Ki67 in FACS-isolated WT and *Cpt2*^{PKO} SCs cultured for 72 h. Scale bar: 10 μm. (**I**) Quantification of total cell number. Error bars represent mean ± s.e.m. with $n = 3$ mice. **$P < 0.01$; two tailed, unpaired Student's *t* test ($P = 0.029$). (**J**) Quantification of percentages of Ki67$^+$ cells. Error bars represent mean ± s.e.m. with $n = 3$ mice (total 8 replicates for WT and 11 replicates for *Cpt2*^{PKO}). ***$P < 0.01$; two tailed, unpaired Student's *t* test ($P = 3 \times 10^{-5}$).

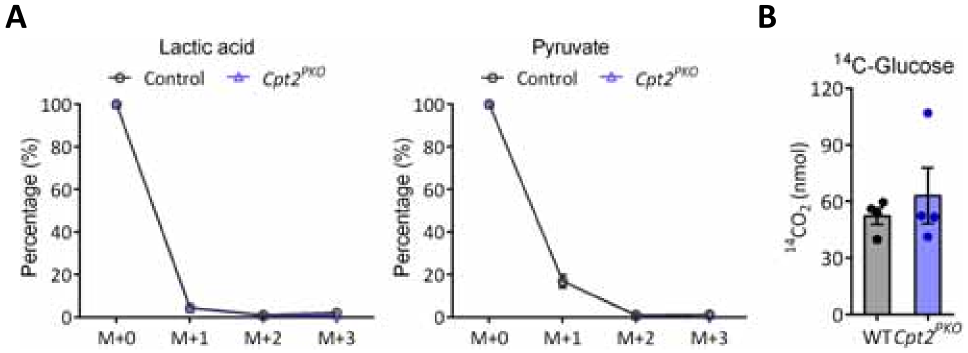

Figure EV7. *Cpt2* deficiency does not affect the glycolytic flux.

(**A**) Targeted metabolite profiling showing no incorporation of ^{13}C derived from ^{13}C-PA in lactate and pyruvate in both WT and *Cpt2*-null myoblasts ($n = 4$, each group). (**B**) Radioactive glycolytic flux measurements using ^{14}C-labeled glucose in WT and *Cpt2*-null satellite cells ($n = 4$, each group).

 © The Author(s)
